# Supplementary material for: Cysteine-rich secreted protein OsCRRSP1 enhances rice defense through dual inhibition of pathogen and host catalases
Source: Plant Commun. 2026 Apr 30;7(9):101878. doi: 10.1016/j.xplc.2026.101878 (PMC13589553; doi:10.1016/j.xplc.2026.101878)
Supplement: Supplementary file 1 [file mmc1.pdf]

Plant Communications, Volume *7*

Supplemental information

Cysteine-rich secreted protein OsCRRSP1 enhances rice defense through dual inhibition of pathogen and host catalases

Zaofa Zhong, Zichao Zheng, Minghui Zhai, Gan Wang, Lijing Zhong, Huakun Zheng, Li Xue, and Haitao Cui

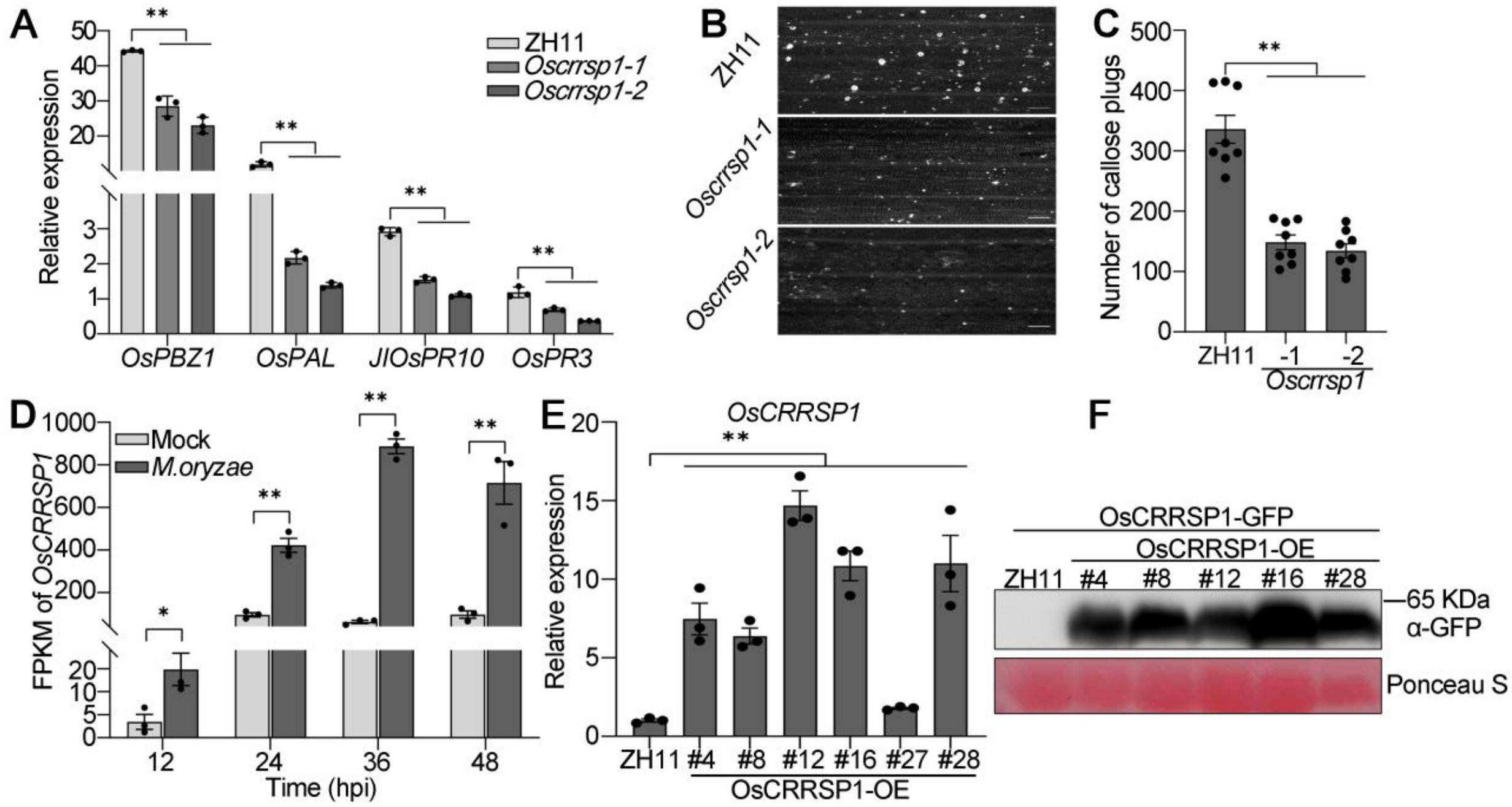

Figure S1. Molecular characterization of *OsCRRSP1* mutants and overexpression lines.

(A) Expression levels of defense-related genes (*OsPBZ1*, *OsPAL*, *JIOsPR10*, *OsPR3*) in ZH11 and *Oscrrsp1* mutants at 48 hpi after inoculation with Guy11, analyzed by qPCR and normalized to *OsUBQ*. Data represent means ± SE ($n = 3$). *$p < 0.05$, **$p < 0.01$ (Student's *t*-test vs. ZH11).

(B) Callose deposition in leaves of ZH11 and *Oscrrsp1* mutants in response to chitin treatment (800 nM), visualized by aniline blue staining under UV fluorescence. Scale bars, 50 μm.

(C) Quantification of callose deposits from (**B**) using ImageJ software. Data represent means ± SE ($n = 8$). *$p < 0.05$, **$p < 0.01$ (Student's *t*-test vs. ZH11).

(D) Transcript levels of *OsCRRSP1* in rice leaves at 12, 24, 36, and 48 hours post-inoculation with *M. oryzae* Guy11 or mock treatment (H_2O), based on RNA-seq data. Data represent mean ± SE ($n = 3$), *$p < 0.05$, **$p < 0.01$ (Student's *t*-test vs. H_2O treatment).

(E) Relative expression of *OsCRRSP1* in leaves of three-week-old ZH11 and OsCRRSP1-OE seedlings, determined by qPCR and normalized to *OsUBQ*. Data represent mean ± SE ($n = 3$); *$p < 0.05$, **$p < 0.01$ (Student's *t*-test vs. ZH11).

(F) Immunoblot analysis of OsCRRSP1-GFP fusion protein in total protein extracts from leaves of four-week-old ZH11 and two independent OsCRRSP1-OE lines, using an anti-GFP antibody. Ponceau S staining of Rubisco large subunit (Rbcl) serves as a loading control.

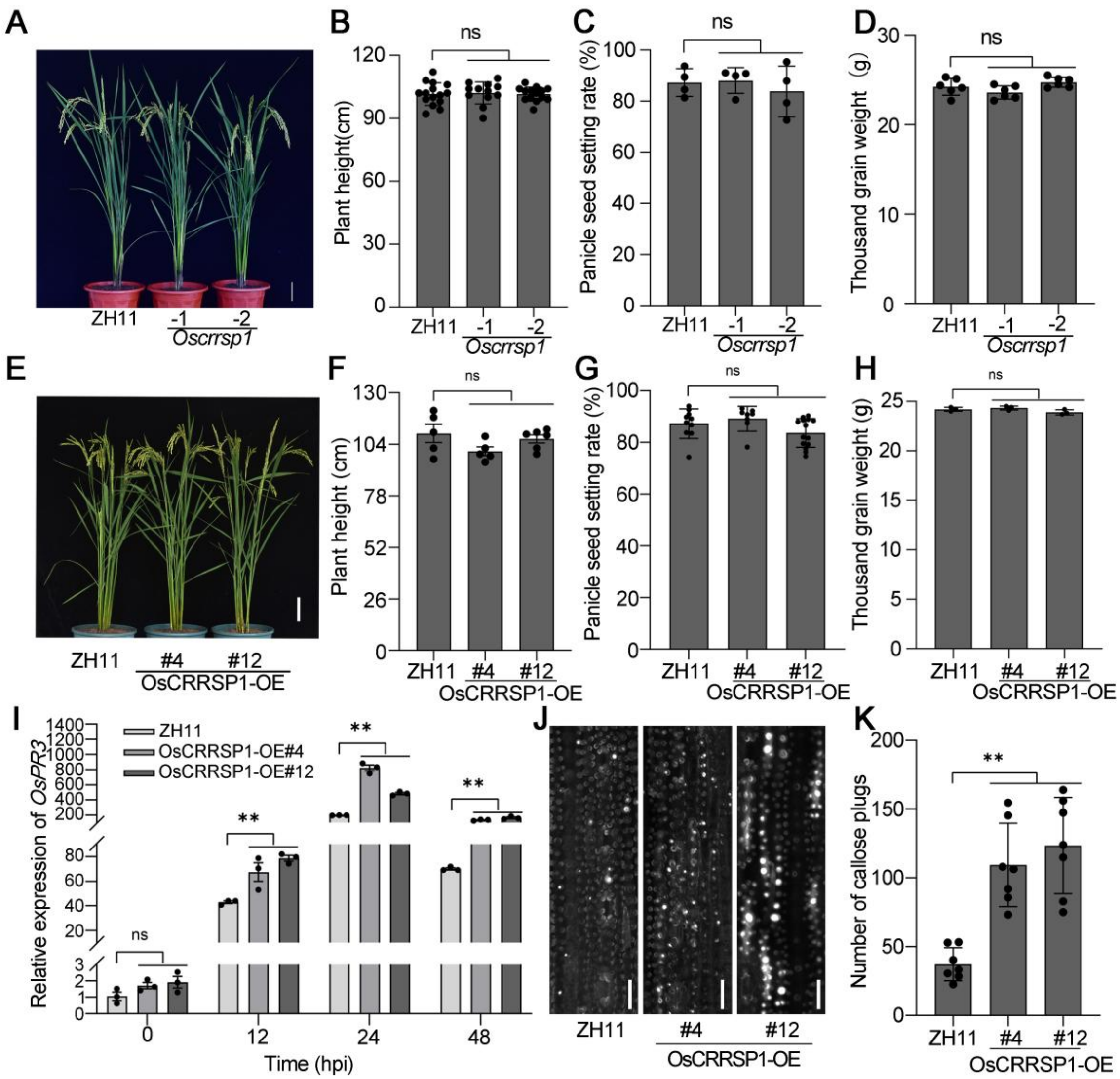

Figure S2. Agronomic traits of OsCRRSP1 mutants and overexpression lines and PTI immune responses in OsCRRSP1-OE plants.

(A) Representative images of ZH11 and *Oscrrsp1* mutant plants at the mature stage under field conditions. Scale bar, 10 cm.

(B) Plant height of ZH11 and *Oscrrsp1* mutants at maturity. Data represent mean ± SE (n = 12); ns, not significant (p > 0.05, Student's t-test).

(C) Panicle seed-setting rate of ZH11 and *Oscrrsp1* mutants. Data represent mean ± SE (n = 4); ns, not significant (p > 0.05, Student's t-test).

(D) Thousand-grain weight of ZH11 and *Oscrrsp1* mutants. Data represent mean ± SE (n = 6), ns, not significant (p > 0.05, Student's t-test).

(E) Field growth phenotypes of ZH11 and OsCRRSP1-OE plants at maturity. Scale bar, 10 cm.

(F) Plant height of ZH11 and OsCRRSP1-OE plants. Data represent mean ± SE ($n = 12$); ns, not significant ($p > 0.05$, Student's *t*-test).

(G) Panicle seed-setting rate of ZH11 and OsCRRSP1-OE plants. Data represent mean ± SE ($n = 4$), ns, not significant ($p > 0.05$, Student's *t*-test).

(H) Thousand-grain weight of ZH11 and OsCRRSP1-OE plants. Data represent mean ± SE ($n = 6$), ns, not significant ($p > 0.05$, Student's *t*-test).

(I) Expression levels of OsPR3 genes in ZH11 and OsCRRSP1-OE plants at indicated time points after *M. oryzae* infection, analyzed by RT–qPCR and normalized to OsUBQ. Data represent means ± SE ($n = 3$). $^{*}p < 0.05$, $^{**}p < 0.01$ (Student’s *t*-test vs. ZH11).

(J) Callose deposition in leaves of ZH11 and OsCRRSP1-OE plants in response to chitin elicitation (800 nM), visualized by aniline blue staining under UV epifluorescence microscopy. Scale bars, 50 μm.

(K) Quantification of callose deposits from (**J**) using ImageJ software. Data represent means ± SE ($n = 7$). $^{*}p < 0.05$, $^{**}p < 0.01$(Student’s *t*-test vs. ZH11).

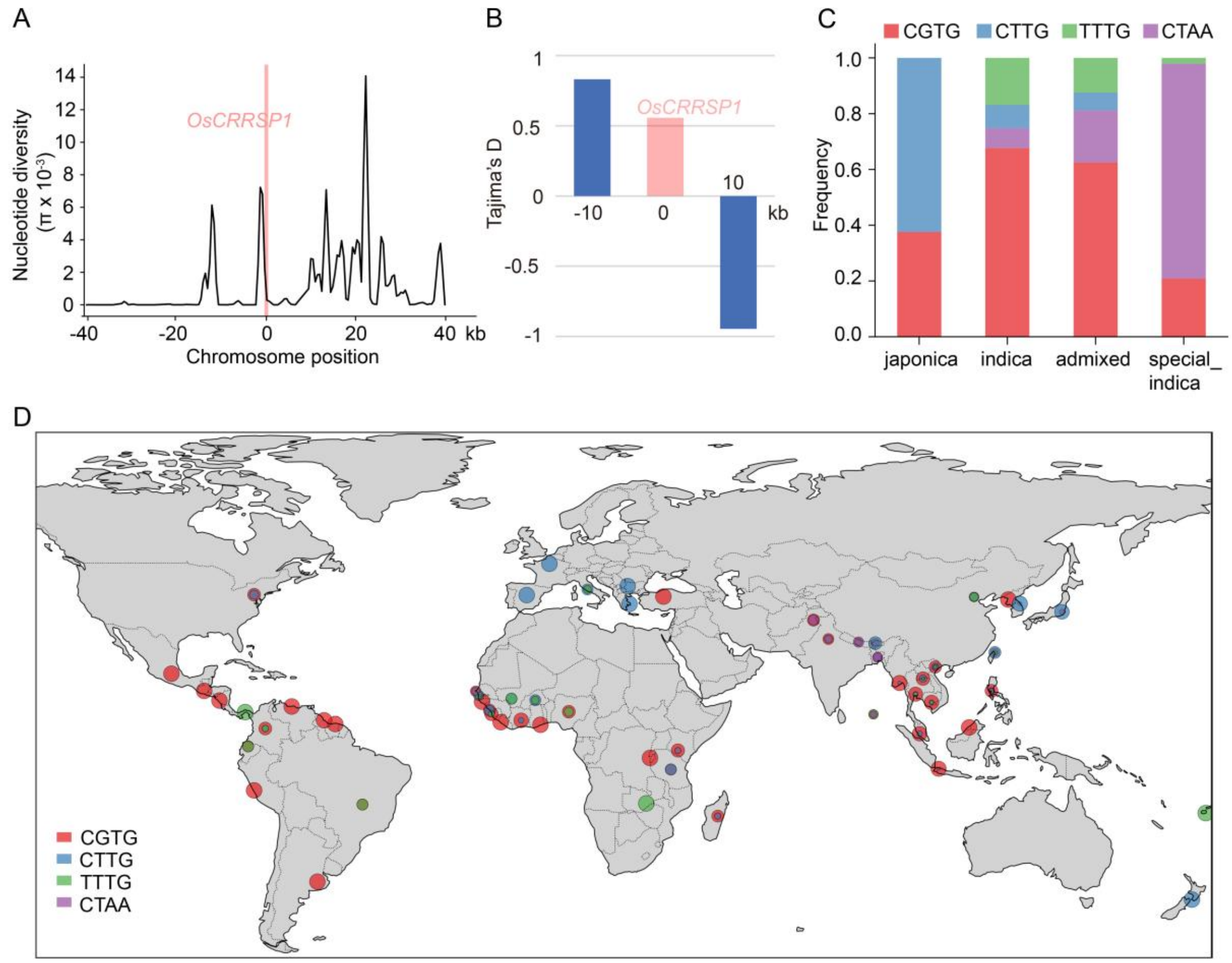

Supplemental Figure 3

Figure S3. Natural variation and geographic distribution of *OsCRRSP1* haplotypes in rice.

(A) Sliding-window analysis of nucleotide diversity (π) across a 40-kb genomic region surrounding *OsCRRSP1* in rice accessions. π was calculated using 1-kb windows with a 500-bp step size. The coding region of *OsCRRSP1* is indicated by a shaded box.

(B) Tajima's D values across *OsCRRSP1* genomic region.

(C) Haplotype composition of *OsCRRSP1* based on four polymorphic sites across rice subpopulations. The frequency of each haplotype (CGTG, CTAA, CTTG, and TTTG) is shown for indica, japonica, and other subgroups.

(D) Geographic distribution of major *OsCRRSP1* haplotypes across global rice accessions. Different colors indicate distinct haplotypes.

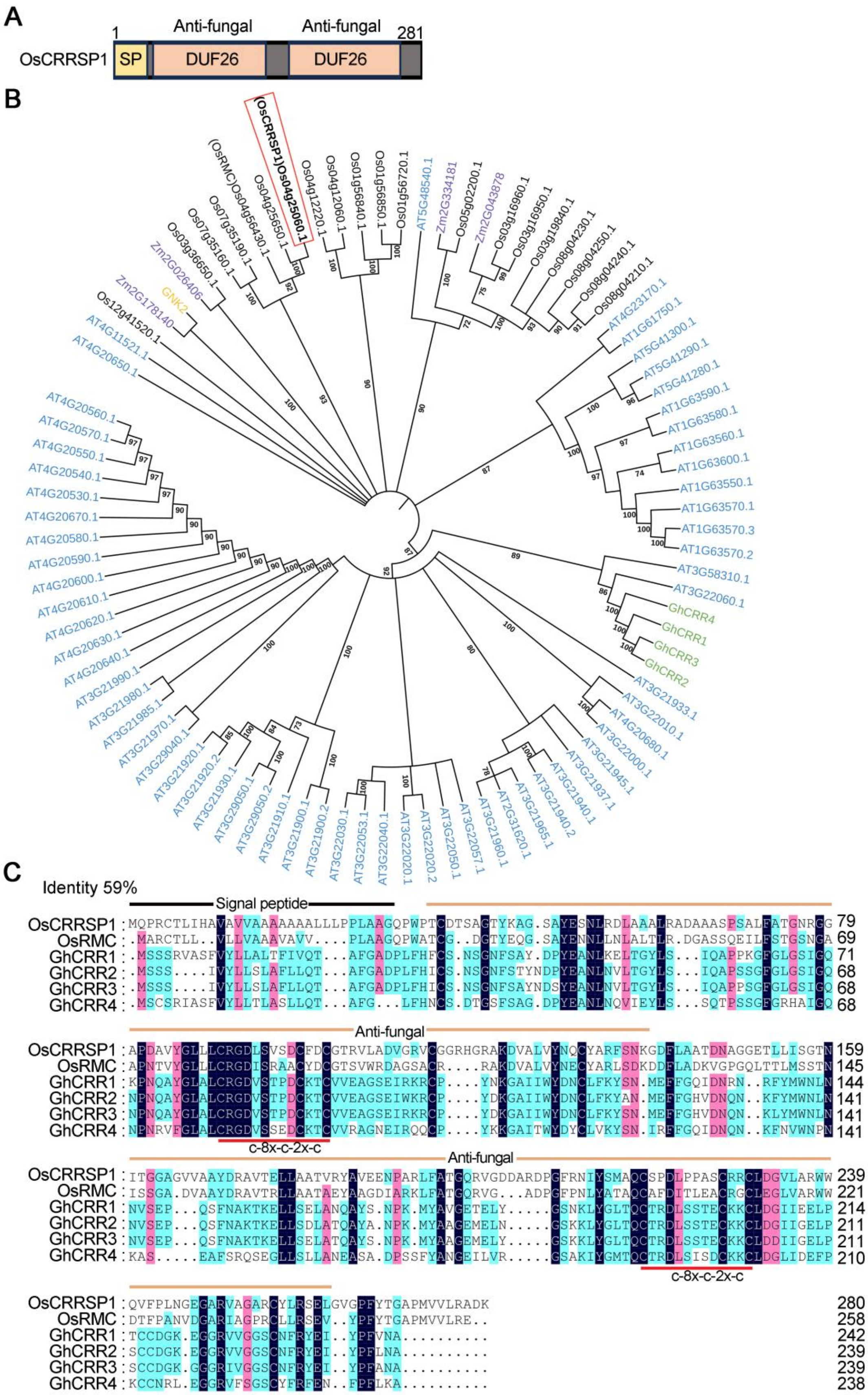
A
OsCRRSP1
1
SP
Anti-fungal
DUF26
Anti-fungal
DUF26
281
B
(OsCRRSP1)Os04g25060.1
(OsRMC)Os04g56430.1
GNK2
C
Identity 59%
Signal peptide
Anti-fungal
Anti-fungal
c-8x-c-2x-c
c-8x-c-2x-c
OsCRRSP1
OsRMC
GhCRR1
GhCRR2
GhCRR3
GhCRR4

Figure S4. Protein domain, evolutionary tree analysis, and Sequence alignment of OsCRRSP1.

(A) Schematic diagram of the OsCRRSP1 protein structure, showing the N-terminal signal peptide (SP) and two domains of unknown function (DUF26). Numbers indicate amino acid (aa) positions.

(B) Phylogenetic tree of CRRSP proteins in rice. The phylogenetic tree was generated using MEGA 7.0. In the phylogenetic tree, CRRSP members from rice are highlighted in black. The gene IDs of CRRSP members from *Arabidopsis thaliana* are marked in blue. Four CRR members from cotton are indicated in green, four CRRSP members from maize are shown in purple, and Gnk2 from *Ginkgo biloba* is labeled in yellow.

(C) Multiple sequence alignment of OsCRRSP1 with its homologs from rice (OsRMC) and cotton (GhCRR1, GhCRR2, GhCRR3, GhCRR4). The conserved cysteine residues forming the characteristic C-8X-C-2X-C motifs in the DUF26 domains are indicated underlined in red. Sequence identities were calculated using Clustal Omega.

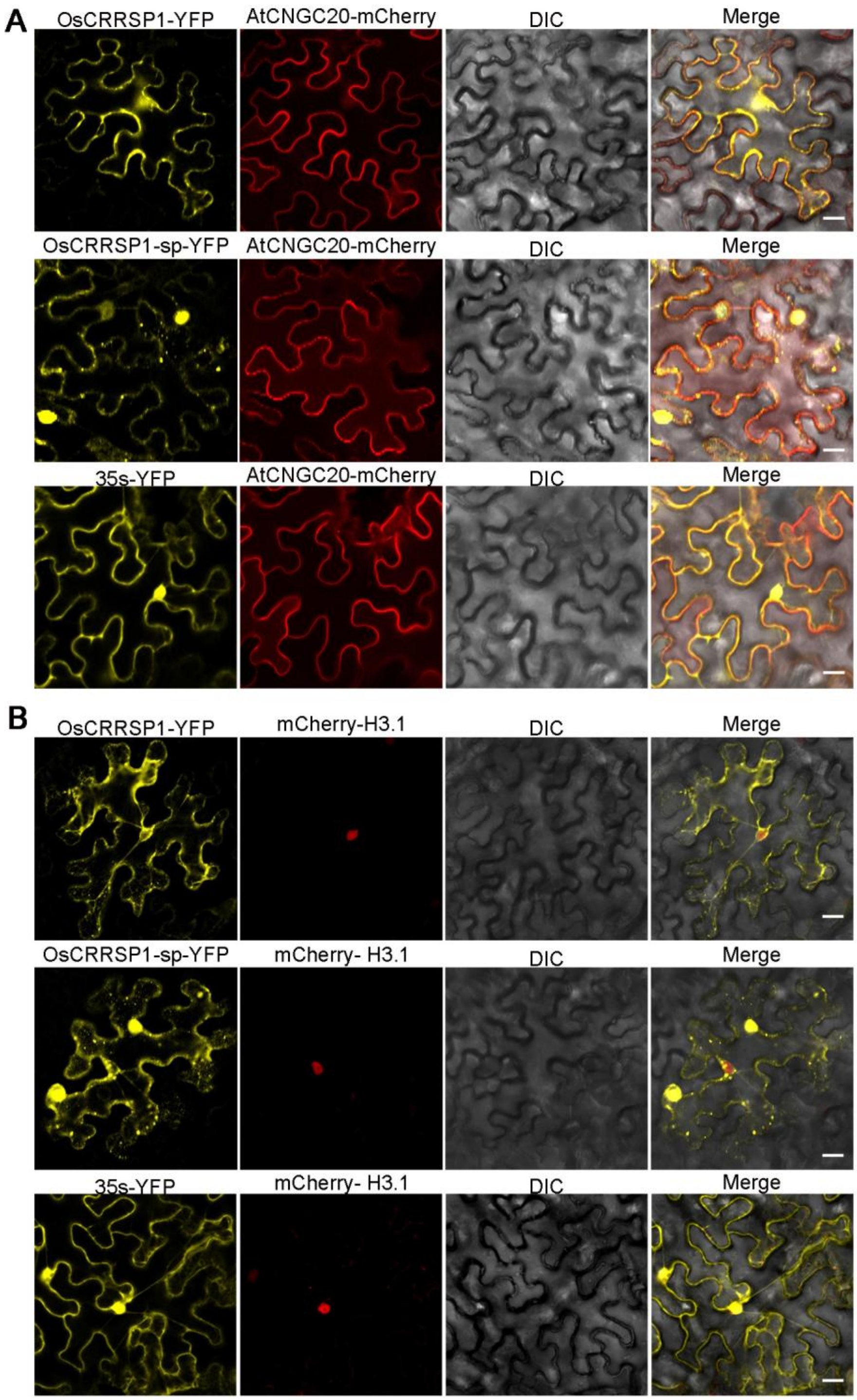

Figure S5 Subcellular localization analysis of OsCRRSP1.

(A) Subcellular localization of full-length OsCRRSP1-YFP and signal peptide-deleted OsCRRSP1$_{\Delta SP}$-YFP (yellow) co-expressed with the plasma membrane marker AtCNGC20-mCherry (red) in *N. benthamiana* epidermal cells. 35S-YFP was used as a cytosolic control. Images were acquired by confocal microscopy 48 hours after agroinfiltration. Scale bar, 20 μm.

(B) Subcellular localization of OsCRRSP1-YFP and $OsCRRSP1_{\Delta SP}$-YFP (yellow) co-expressed with the nuclear marker mCherry-Histone H3.1 (red) in *N. benthamiana* epidermal cells. 35S-YFP was used as a cytosolic control. Images were acquired by confocal microscopy 48 hours after agroinfiltration. Scale bar, 20 μm.

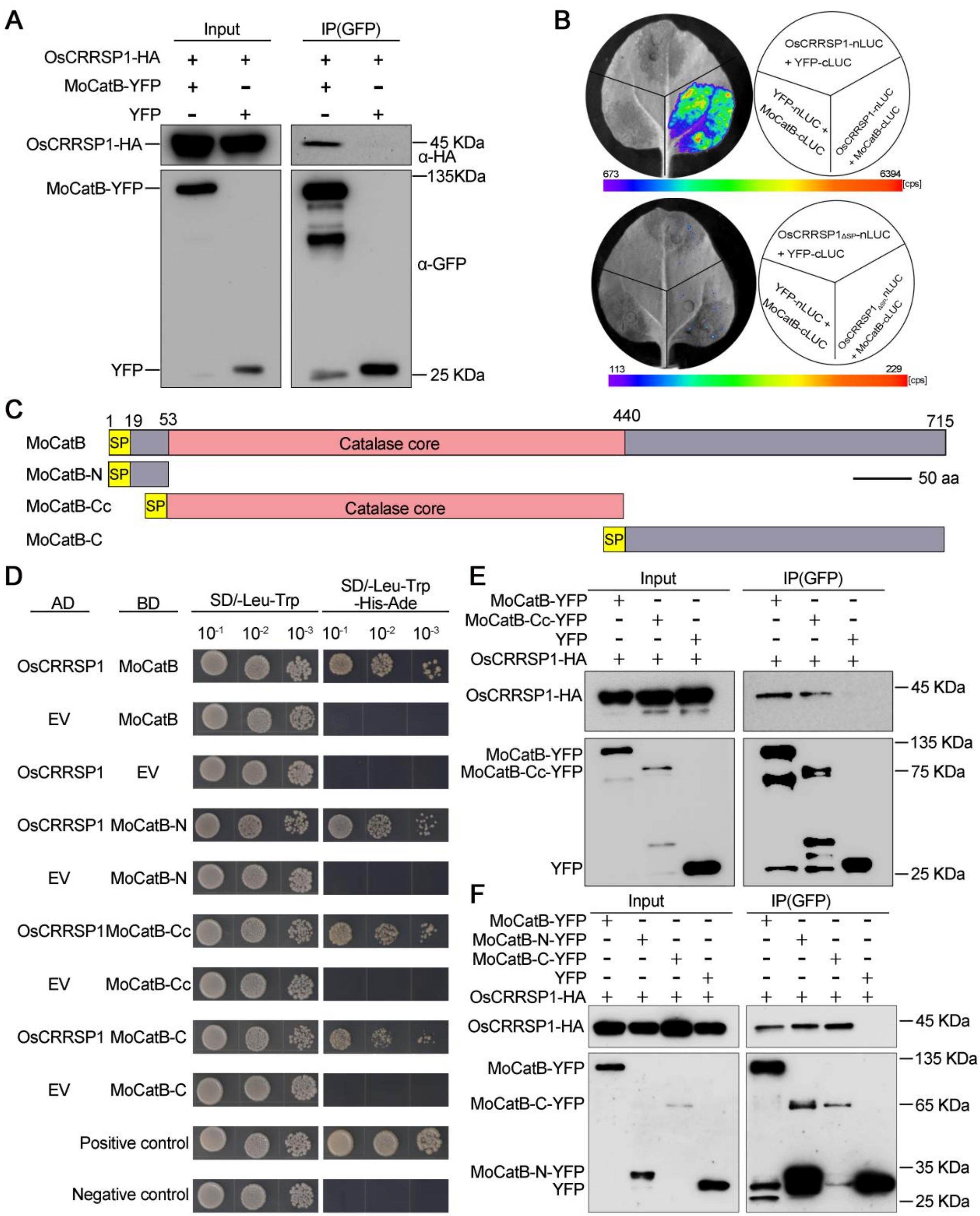

Figure S6. OsCRRSP1 interacts with MoCatB through multiple regions.

(A) Co-immunoprecipitation (co-IP) analysis of the interaction between MoCatB and OsCRRSP1 *in planta*. Total proteins were extracted from *N. benthamiana* leaves co-expressing MoCatB-GFP and OsCRRSP1-HA, or the control (YFP and OsCRRSP1-HA), at 48 hours post-agroinfiltration. Proteins were immunoprecipitated (IP)

using GFP-Trap beads and subsequently immunoblotted with the indicated antibodies. "Input" represents 2.5% of the total protein extract used for the IP. Molecular mass markers are indicated in kilodaltons (kD).

(B) Split-luciferase complementation assay testing the interaction between signal peptide-deleted OsCRRSP1 (OsCRRSP1$_{\Delta SP}$-nLUC) and MoCatB-cLUC in *N. benthamiana* leaves. The combinations OsCRRSP1$_{\Delta SP}$-nLUC + cLUC and nLUC + MoCatB-cLUC served as negative controls. Luciferase activity was imaged 48 hours post-infiltration.

(C) Schematic diagram of the MoCatB protein structure and construction of truncated vector. The yellow box represents the predicted N-terminal signal peptide (SP), the pink box represents the predicted Catalase core (Cc) domain. The SP shown in this diagram were all removed in the Y2H experiment, while they were retained in the Co-IP experiment. Numbers indicate amino acid (aa) positions.

(D) Yeast two-hybrid assays examining interactions of OsCRRSP1 with MoCatB-N, MoCatB-Cc, and MoCatB-C. Yeast strain AH109 co-transformed with the indicated bait (BD) and prey (AD) constructs was grown on synthetic defined medium lacking Leu and Trp (SD/-Leu/-Trp) or selective medium lacking Leu, Trp, His, and Ade (SD/-Ade/-His/-Leu/-Trp). The AD-T + BD-53 pair served as a positive control, while AD + BD, AD + BD-MoCatB-N, AD + BD-MoCatB-Cc, AD + BD-MoCatB-C and AD-OsCRRSP1 + BD were negative controls. Plates were incubated at 30°C for 3 days.

(E) Co-immunoprecipitation (Co-IP) analysis of the interaction between OsCRRSP1 and the catalase core domain of MoCatB (MoCatB-Cc) in planta. Total proteins were extracted from *N. benthamiana* leaves co-expressing OsCRRSP1-HA and MoCatB-Cc -GFP, or the control (YFP and OsCRRSP1-HA), at 48 hours post-agroinfiltration. Proteins were immunoprecipitated (IP) using GFP-Trap beads and subsequently immunoblotted with the indicated antibodies. "Input" represents 2.5% of the total protein extract used for the IP.

(F) Co-immunoprecipitation (Co-IP) analysis of the interactions of MoCatB-N and MoCatB-C with OsCRRSP1 in planta. Total proteins were extracted from rice protoplasts co-expressing MoCatB-GFP and OsCRRSP1-HA, MoCatB-N-GFP and OsCRRSP1-HA, MoCatB-C-GFP and OsCRRSP1-HA or the control (YFP and OsCRRSP1-HA). Proteins were immunoprecipitated (IP) using GFP-Trap beads and subsequently immunoblotted with the indicated antibodies. "Input" represents 2.5% of the total protein extract used for the IP.

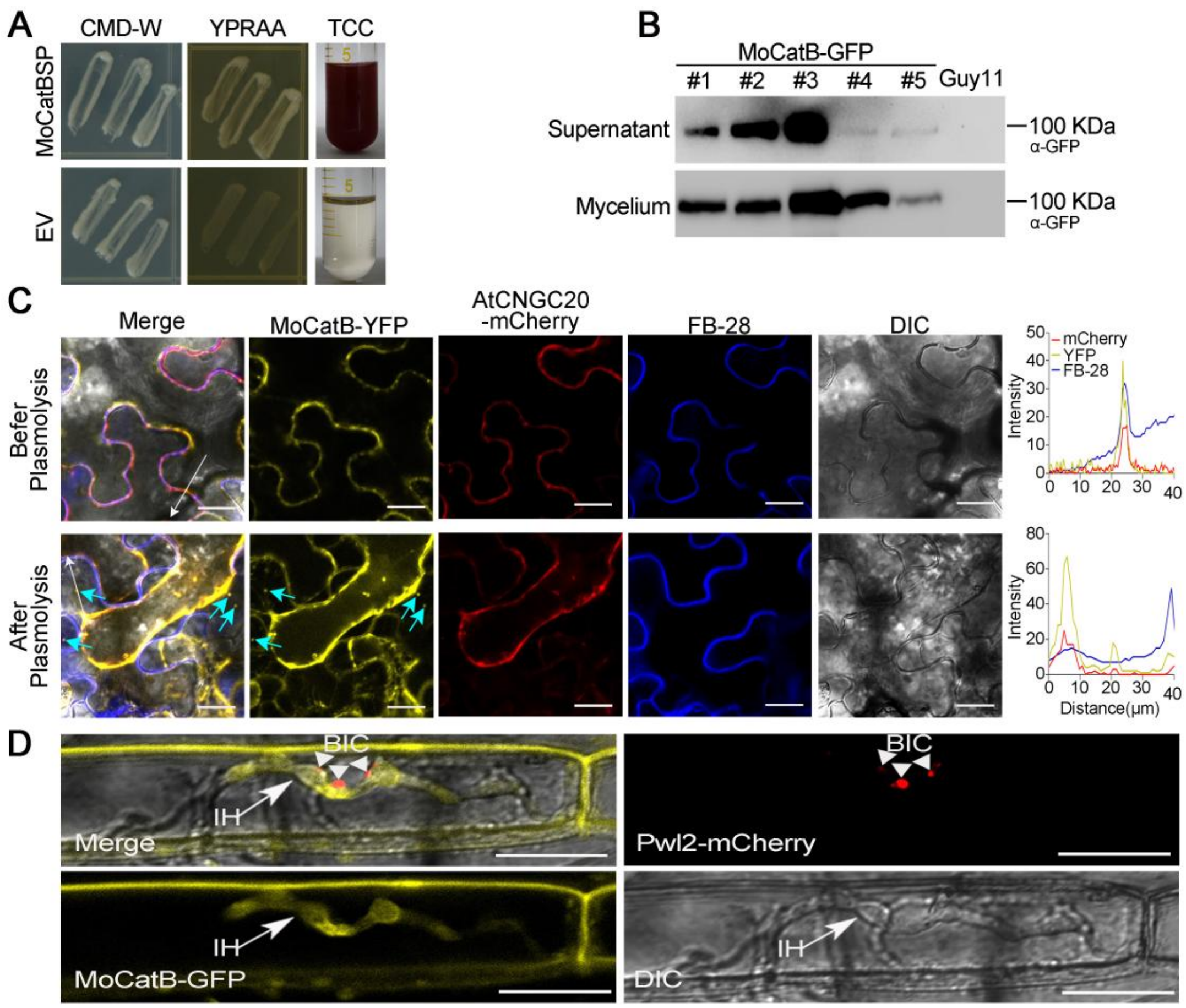

Figure S7 ***M. oryzae*** **MoCatB is a secreted protein.**

(A) Functional analysis of the MoCatB signal peptide (MoCatBSP) using the yeast invertase secretion assay. Yeast strain YTK12 expressing pSUC2-MoCatBSP or the empty pSUC2 vector (negative control) were grown on CMD-W (tryptophan-deficient) or YPRAA (raffinose-based) medium. Invertase secretion was confirmed by the reduction of triphenyltetrazolium chloride (TTC) to red formazan.

(B) Immunoblot analysis detecting MoCatB-GFP secretion in *M. oryzae*. Proteins from mycelial extracts (Mycelium) and culture supernatant (Supernatant) of these MoCatB-GFP strains (#1-5) were probed with an anti-GFP antibody. The wild-type Guy11 strain served as a negative control.

(C) MoCatB is located in the plant apoplast. Subcellular localization of MoCatB-YFP (yellow) co-expressed with the plasma membrane marker AtCNGC20-mCherry (red) in *N. benthamiana* epidermal cells before and after plasmolysis. Cell walls were stained with Fluorescent Brightener 28 (FB-28; blue). Images were captured by confocal microscopy before and after plasmolysis induced by 1.5 M sorbitol. Blue arrows indicate MoCatB-YFP signal in the apoplastic space post-plasmolysis. The fluorescence curves were obtained following the direction of the white arrows. Scale bars, 20 μm.

(D) Confocal microscopy images of rice sheath cells infected with an *M. oryzae* strain co-expressing MoCatB-GFP (green) and the cytoplasmic effector Pwl2-mCherry (red) at 24 hours post-inoculation (hpi). The biotrophic

interfacial complex (BIC) is indicated by a white arrowhead. Scale bars, 20 μm.

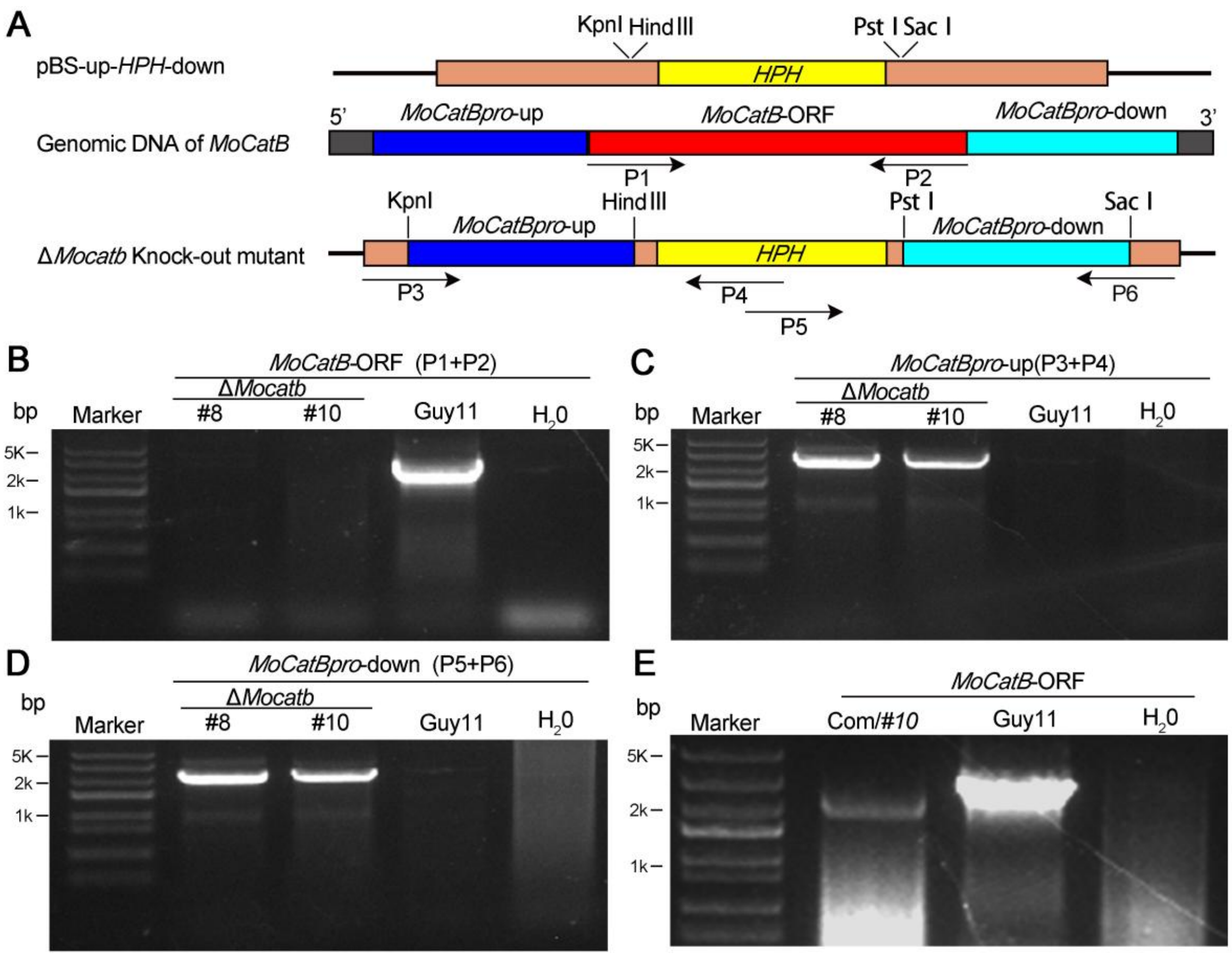

Figure S8. Generation and molecular characterization of *MoCatB* knockout and complementation strains.

(A) Strategy for targeted deletion of *MoCatB* in *M. oryzae* strain Guy11 via homologous recombination. The hygromycin phosphotransferase (*HPH*) cassette replaces the *MoCatB* coding sequence. Arrows indicate primer positions (P1-P4) used for mutant verification. Schematic not drawn to scale.

(B) PCR verification of *ΔMocatb* mutants using primers spanning the full-length *MoCatB* open reading frame (ORF). Genomic DNA from Guy11 produces the expected amplicon, while no product is amplified from the *ΔMocatb* mutants (#8 and #10).

(C) PCR verification of *ΔMocatb* mutants using primers specific to the upstream flanking region of *MoCatB*. The expected fragment is present in both Guy11 and the mutants.

(D) PCR verification of *ΔMocatb* mutants using primers specific to the downstream flanking region of *MoCatB*. The expected fragment is present in both Guy11 and the mutants.

(E) PCR verification of the complemented strain (Com/#10) using primers specific to the *MoCatB* ORF. Guy11 and Com/#10 show the expected amplification, while no product is detected in the *ΔMocatb*#10 mutant. H_2O serves as a no-template control.

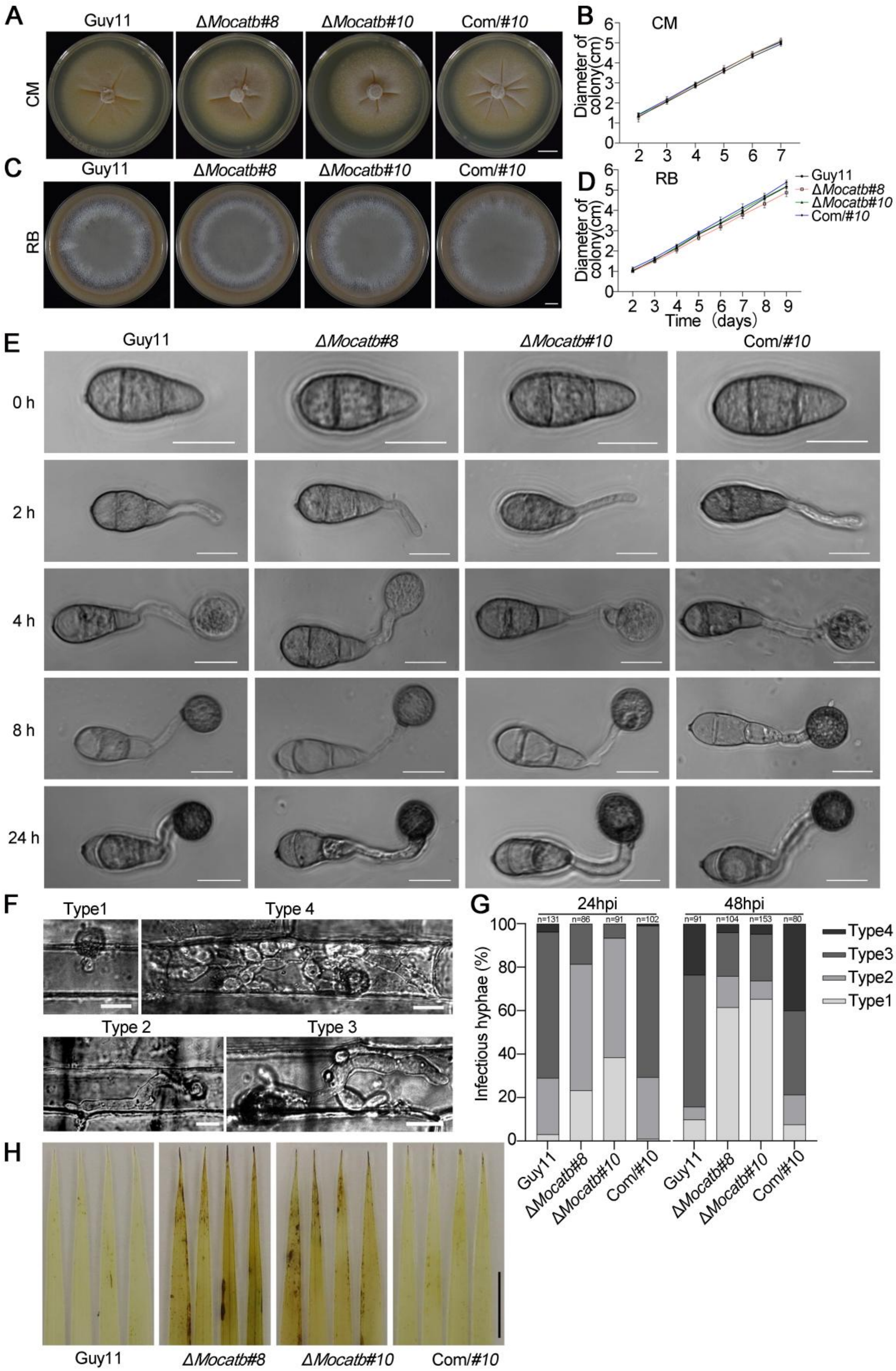

A
Guy11
ΔMocatb#8
ΔMocatb#10
Com/#10
CM
B
CM
Diameter of colony(cm)
C
RB
D
RB
Guy11
ΔMocatb#8
ΔMocatb#10
Com/#10
Time (days)
E
0 h
2 h
4 h
8 h
24 h
F
Type1
Type 4
Type 2
Type 3
G
24hpi
48hpi
n=131
n=86
n=91
n=102
n=91
n=104
n=153
n=80
Infectious hyphae (%)
Type4
Type3
Type2
Type1
H

149

Figure S9. *MoCatB* deletion does not affect *in vitro* growth but impairs invasive hyphal development and host ROS suppression.

(A) Colony morphology of Guy11, *ΔMocatb* mutants (#8, #10), and complemented strain (Com/#10) after 7 days of growth on complete medium (CM) at 28°C.

(B) Mycelial growth rates of strains in (**A**). Data represent mean ± SE (n = 6).

(C) Colony morphology of indicated strains after 9 days of growth on rice bran (RB) medium at 28°C.

(D) Mycelial growth rates of strains in (**C**). Data represent mean ± SE (n = 6).

(E) Conidial germination and appressorium formation of indicated strains on hydrophobic surfaces at 2, 4, and 8 hours post-incubation. Scale bar, 10 µm.

(F) Classification of invasive hyphal (IH) types in rice sheath cells: Type 1, filamentous primary IH; Type 2, single bulbous IH; Type 3, branched bulbous IH; Type 4, IH occupying entire plant cell and penetrating neighboring cells. Scale bar, 10 µm.

(G) Percentage distribution of IH types at 24 and 48 hours post-inoculation (hpi) with indicated strains. At least 80 infection sites were examined per strain per time point across three independent experiments.

(H) DAB staining detecting H_2O_2 accumulation in ZH11 leaves at 48 hpi with Guy11, *ΔMocatb* mutants (#8, #10), or Com/#10. Scale bar, 1 cm.

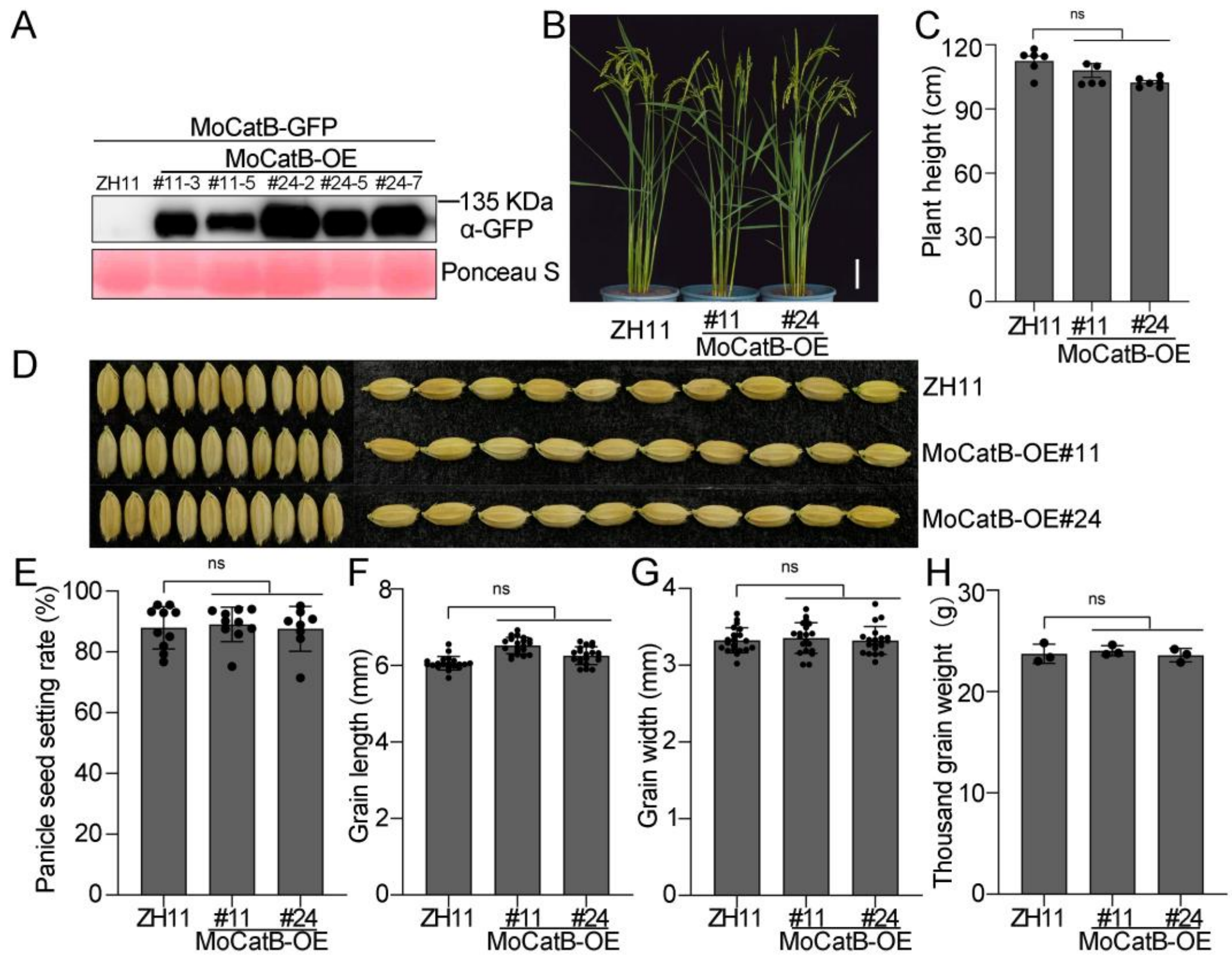

Figure S10. Overexpression of *MoCatB* does not affect major agronomic traits in rice.

(A) Immunoblot analysis of MoCatB-GFP fusion protein in total protein extracts from leaves of three-week-old ZH11 and two independent MoCatB-OE lines using an anti-GFP antibody. Ponceau S staining of Rubisco large subunit (Rbcl) serves as a loading control.

(B) Representative images of ZH11 and MoCatB-OE plants at the mature stage under field conditions. Scale bar, 10 cm.

(C) Plant height of ZH11 and MoCatB-OE plants at maturity. Data represent mean ± SE (n = 12). ns, not significant.

(D) Mature seeds of ZH11 and MoCatB-OE plants. Scale bar, 1 cm.

(E) Panicle seed-setting rate of ZH11 and MoCatB-OE plants. Data represent mean ±SE (n = 4). ns, not significant. *$p < 0.05$ (Student's t -test).

(F) Grain length of ZH11 and MoCatB-OE plants. Data represent mean ± SE (n = 20). ns, not significant. *$p < 0.05$ (Student's t -test)

(G) Grain width of ZH11 and MoCatB-OE plants. Data represent mean ± SE (n = 20). ns, not significant. *$p < 0.05$ (Student's t -test)

(H) Thousand-grain weight of ZH11 and MoCatB-OE plants. Data represent mean ± SE (n = 6). ns, not significant. *$p < 0.05$ (Student's t -test).

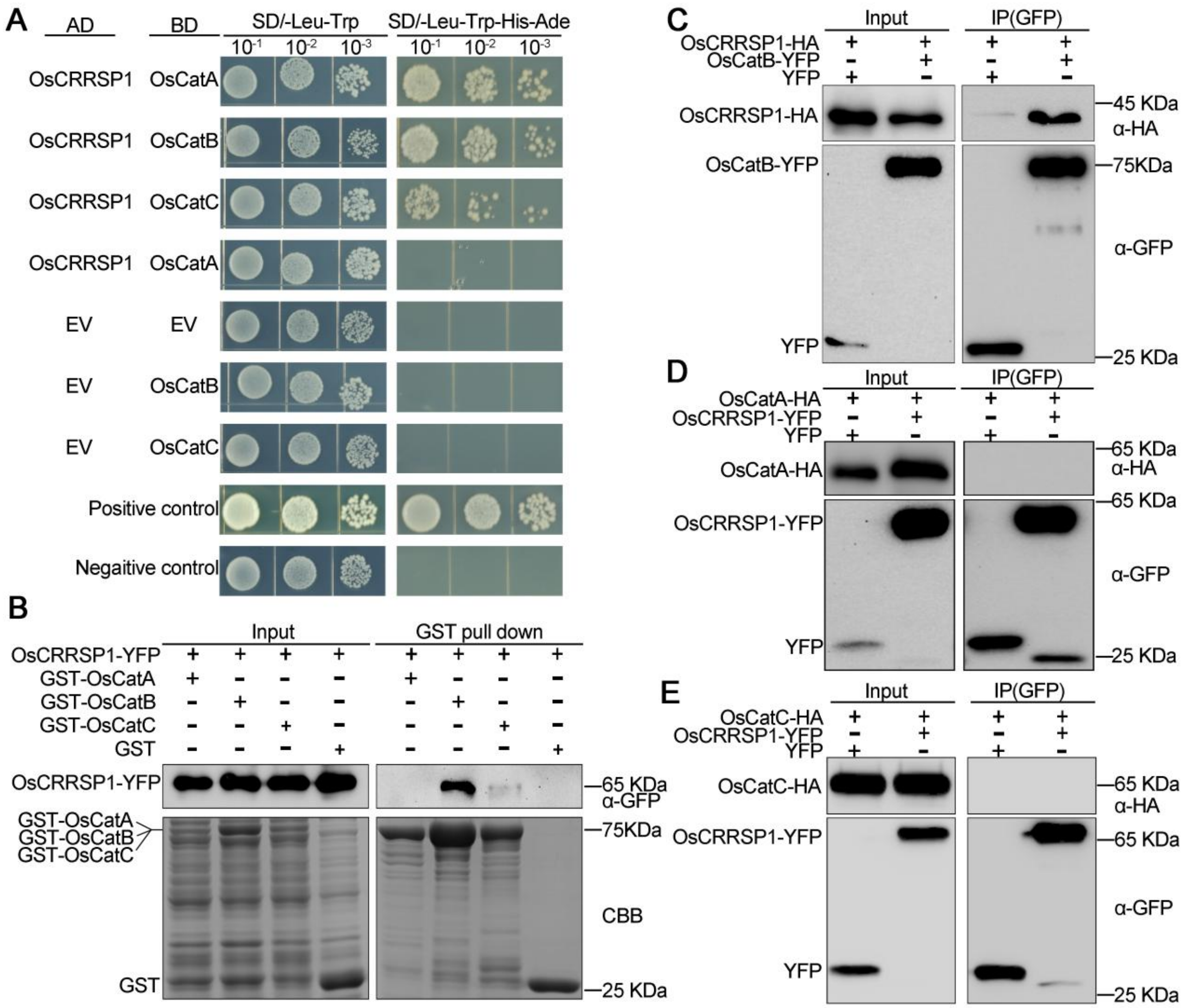

Figure S11. Specificity analysis of OsCRRSP1 interaction with rice catalase family members.

(A) Yeast two-hybrid assay screening the interaction between OsCRRSP1 and three rice catalases (OsCatA, OsCatB, OsCatC). Yeast strain AH109 co-expressing the indicated bait (BD) and prey (AD) constructs was grown on synthetic defined medium lacking Leu and Trp (SD/-Leu/-Trp) or selective medium lacking Leu, Trp, His, and Ade (SD/-Ade/-His/-Leu/-Trp). The AD-T + BD-53 pair served as a positive control, while the combinations AD + BD, AD + BD-OsCatA/B/C, and AD-OsCRRSP1 + BD served as negative controls. Plates were incubated at 30°C for 3 days.

(B) GST pull-down assays assessing direct interactions between OsCRRSP1 and rice catalases. Recombinant GST-OsCatA, GST-OsCatB, GST-OsCatC or GST (control) immobilized on glutathione-sepharose beads were incubated with purified OsCRRSP1-YFP. Bound proteins were analyzed by immunoblotting with an anti-GFP antibody (top panels). The bottom panels show Coomassie Brilliant Blue (CBB) staining of the bait proteins.

(C) Co-IP analysis of the interaction between OsCatB and OsCRRSP1 in planta. Total proteins were extracted from *N. benthamiana* leaves co-expressing OsCatB-YFP and OsCRRSP1-HA, or the control (YFP and

OsCRRSP1-HA), at 48 hours post-agroinfiltration. Proteins were immunoprecipitated (IP) using GFP-Trap beads and subsequently immunoblotted with the indicated antibodies. "Input" represents 2.5% of the total protein extract used for the IP.

(D) Co-IP assay testing the interaction between OsCRRSP1 and OsCatA *in planta*. Total proteins were extracted from *N. benthamiana* leaves co-expressing OsCRRSP1-YFP and OsCatA-HA, or the control (YFP and OsCatA-HA), at 48 hours post-agroinfiltration. Proteins were immunoprecipitated using GFP-Trap beads and immunoblotted with the indicated antibodies. "Input" represents 2.5% of the total protein extract.

(E) Co-IP assay testing the interaction between OsCRRSP1 and OsCatC *in planta*. Experimental conditions and controls are as described in panel (**D**).

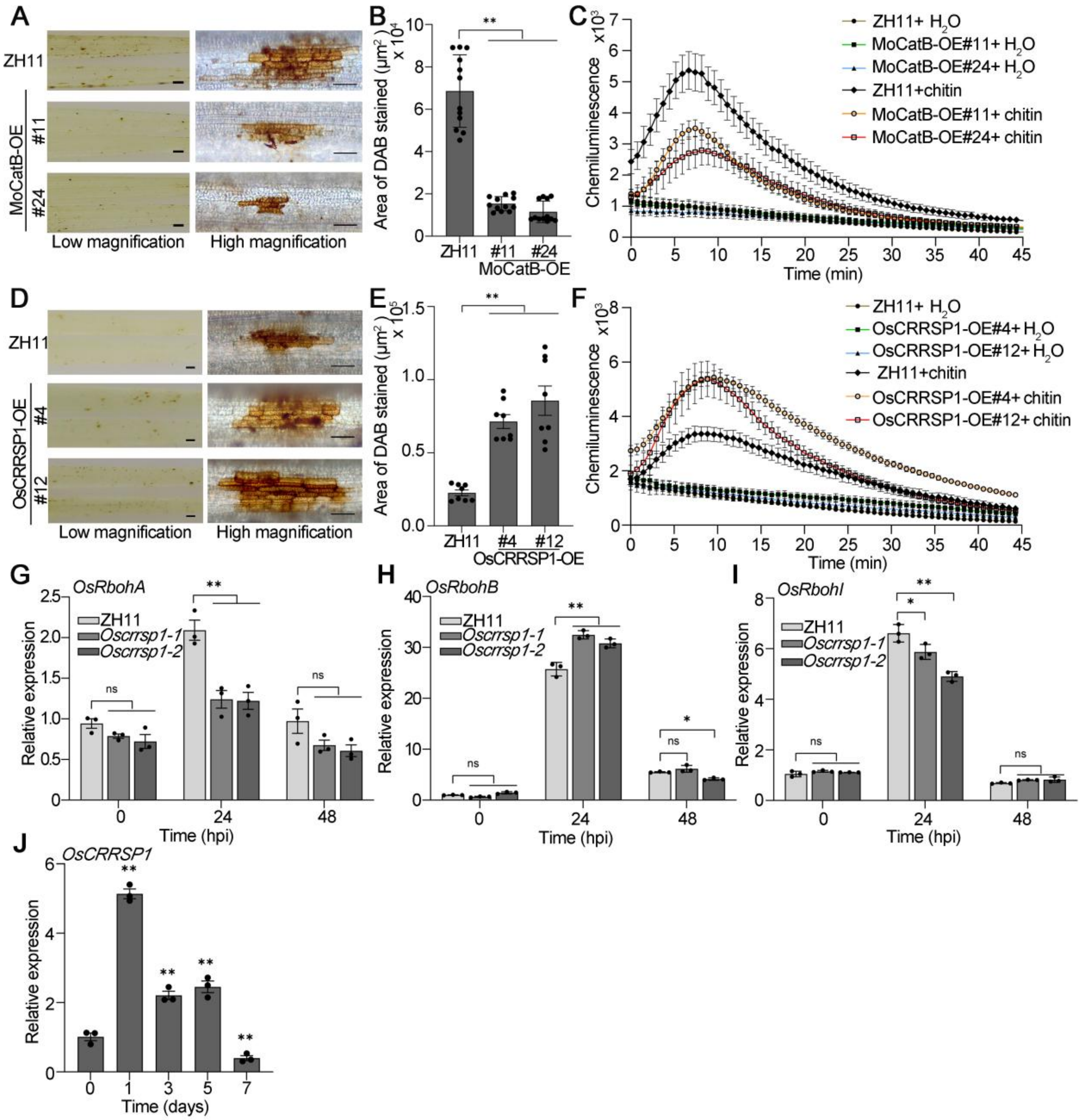

Figure S12 OsCRRSP1 promotes pathogen-induced ROS accumulation.

(A) Detection of H_2O_2 accumulation by 3,3'-diaminobenzidine (DAB) staining in leaves of ZH11 and *MoCatB-OE* plants at 48 hours post-inoculation with *M. oryzae* Guy11. Low-magnification (top) and high-magnification (bottom) images are shown. Scale bars, 1 mm (top) or 100 μm (bottom).

(B) Quantification of DAB-stained area from (**A**) using ImageJ. Data represent mean ± SE (n = 8). *$p < 0.05$, **$p < 0.01$(Student's t-test vs. ZH11).

(C) Chitin-induced ROS burst in leaf discs of ZH11 and *MoCatB-OE* plants treated with 800 nM chitin. Luminescence was measured over 45 minutes. Data represent mean ± SE (n = 12).

(D) DAB staining detecting H_2O_2 accumulation in ZH11 and OsCRRSP1-OE plants at 48 hpi with Guy11. Scale

bars, 1 mm (top) or 100 μm (bottom).

(E) Quantification of DAB-stained area from (**D**) by ImageJ. Data represent mean ± SE ($n = 8$). *$p < 0.05$, **$p < 0.01$ (Student's t-test vs. ZH11).

(F) Chitin-induced ROS burst in ZH11 and OsCRRSP1-OE leaf discs treated with 800 nM chitin. Data represent mean ± SE (n=8).

(G) Expression levels of *OsRbohA* in ZH11 and *Oscrrsp1* mutants after inoculation with Guy11, analyzed by RT-qPCR and normalized to *OsUBQ*. Data represent means ± SE ($n = 3$). *$p < 0.05$, **$p < 0.01$ (Student's t-test vs. ZH11).

(H) Expression levels of *OsRbohB* in ZH11 and *Oscrrsp1* mutants after inoculation with Guy11, analyzed by RT-qPCR and normalized to *OsUBQ*. Data represent means ± SE ($n = 3$). *$p < 0.05$, **$p < 0.01$ (Student's t-test vs. ZH11).

(I) Expression levels of *OsRbohI* in ZH11 and *Oscrrsp1* mutants after inoculation with Guy11, analyzed by RT-qPCR and normalized to *OsUBQ*. Data represent means ± SE ($n = 3$). *$p < 0.05$, **$p < 0.01$ (Student's t-test vs. ZH11).

(J) Expression analysis of *OsCRRSP1* in rice leaves inoculated with *Xanthomonas oryzae* pv. *Oryzae*. Samples were collected at the indicated time points and analyzed by RT-qPCR normalized to *OsUBQ*. The data represent mean ± SE ($n = 3$). **$p < 0.01$ (Student's t-test vs. 0 days).

Table S1. (separate file)

Haplotype composition of *OsCRRSP1* based on four polymorphic sites across rice subpopulations.

Table S2. (separate file)

Putative OsCRRSP1-interacting proteins identified by IP-MS in *M. oryzae*.

Table S3. (separate file)

The candidate proteins revealed by the Y2H screen.

Table S4. (separate file)

Primers used in this study.
