# Supplementary material for: Cysteine-rich secreted protein OsCRRSP1 enhances rice defense through dual inhibition of pathogen and host catalases
Source: Plant Commun. 2026 Apr 30;7(9):101878. doi: 10.1016/j.xplc.2026.101878 (PMC13589553; doi:10.1016/j.xplc.2026.101878)
Supplement: Supplementary file 2 [file mmc6.pdf]

Plant Communications
Research article

CellPress Partner Journal

Cysteine-rich secreted protein OsCRRSP1 enhances rice defense through dual inhibition of pathogen and host catalases

Zaofa Zhong[1,2], Zichao Zheng[3], Minghui Zhai[4], Gan Wang[3], Lijing Zhong[4], Huakun Zheng[5], Li Xue[1,2,*] and Haitao Cui[1,4,*]

[1]State Key Laboratory of Wheat Improvement, Shandong Agricultural University, Tai'an, Shandong, China

[2]College of Life Sciences, Shandong Agricultural University, Tai'an 271000, China

[3]Plant Immunity Center, Fujian Agriculture and Forestry University, Fujian 350000, China

[4]College of Plant Protection, Shandong Agricultural University, Tai'an 271000, China

[5]State Key Laboratory of Ecological Pest Control for Fujian and Taiwan Crops, College of Plant Protection, Fujian Agriculture and Forestry University, Fuzhou 350000, China

***Correspondence: Li Xue (xue.li@sdau.edu.cn), Haitao Cui (cuihaitao@sdau.edu.cn)**

https://doi.org/10.1016/j.xplc.2026.101878

ABSTRACT

Plants utilize reactive oxygen species (ROS) as key defense signals, whereas pathogens have evolved mechanisms to disrupt ROS homeostasis. However, plant factors that directly counteract pathogen-derived ROS-scavenging effectors remain elusive. Here, we identified cysteine-rich receptor-like secreted protein 1 (OsCRRSP1) as a positive regulator of rice resistance to *Magnaporthe oryzae*. *OsCRRSP1* expression is strongly induced upon pathogen infection; genetic analyses showed that knockout mutants are more susceptible, whereas overexpression enhances resistance. OsCRRSP1 directly interacts with both the fungal catalase MoCatB and the rice catalase OsCatB and inhibits their H_2O_2 degrading activities, leading to reduced catalase activity and enhanced ROS accumulation. Consequently, *OsCRRSP1* overexpression elevates H_2O_2 levels, whereas *MoCatB* overexpression suppresses ROS accumulation and promotes disease development. These findings uncover a previously unrecognized OsCRRSP1–MoCatB/OsCatB module that fine-tunes ROS homeostasis to strengthen rice immunity and provides a promising molecular target for resistance breeding.

Key words: rice innate immunity, *Magnaporthe oryzae*, reactive oxygen species, cysteine-rich receptor-like secreted proteins, catalase

Zhong Z., Zheng Z., Zhai M., Wang G., Zhong L., Zheng H., Xue L., and Cui H. (2026). Cysteine-rich secreted protein OsCRRSP1 enhances rice defense through dual inhibition of pathogen and host catalases. Plant Comm. **7**, 101878.

INTRODUCTION

Plants are constantly exposed to diverse microbial pathogens and have evolved multilayered immune systems to detect and counter infection (Jones and Dangl, 2006; Ngou et al., 2022). Among these defense layers, apoplastic immunity, which operates in the extracellular space before pathogen entry, represents the first line of defense (Dietz, 1997; Dora et al., 2022; Del Corpo et al., 2024). In this compartment, plants deploy receptors, inhibitors, and antimicrobial proteins to intercept pathogen effectors. For example, *Phytophthora sojae* secretes the cell wall-degrading enzyme PsXEG1, which is neutralized by the soybean inhibitor GmGIP1 (Ma et al., 2017). Similarly, the *Magnaporthe oryzae* effector MoChia1 sequesters chitin to suppress immunity, whereas rice tetratricopeptide repeat protein 1 (TRP1) counteracts this activity by releasing chitin fragments that reactivate defense signaling (Yang et al., 2019). These examples illustrate how the apoplast serves as a biochemical battlefield where plant and pathogen proteins directly compete to determine infection outcomes.

A central event in apoplastic immunity is the burst of reactive oxygen species (ROS) following pathogen recognition (Apostol et al., 1989; Doehlemann and Hemetsberger, 2013; Qi et al., 2017). ROS not only damage invading pathogens but also function as secondary messengers that amplify immune signaling. Because excessive ROS can be cytotoxic, plants tightly regulate ROS homeostasis through coordinated

 © 2026 The Author(s). Published by Elsevier Inc. on behalf of CAS Center for Excellence in Molecular Plant Sciences, Chinese Academy of Sciences, and Chinese Society for Plant Biology. This is an open access article under the CC BY license (http://creativecommons.org/licenses/by/4.0/).

production and scavenging (Mittler et al., 2022). Among ROS, hydrogen peroxide (H_2O_2) is relatively stable and diffuses across membranes, making it a key signaling molecule in stress responses. Catalases (CATs), which decompose H_2O_2, therefore play dual roles in maintaining redox balance and modulating defense intensity. In *Arabidopsis*, AtCat1–AtCat3 coordinate H_2O_2 scavenging (Mhamdi et al., 2010), whereas in rice, OsCatB and OsCatC regulate immune responses. For instance, the immune suppressor ROD1 activates OsCatB in a Ca^{2+}-dependent manner, promoting H_2O_2 degradation (Gao et al., 2021). Similarly, the uridine diphosphate xylose synthase OsUXS3 and the calcium-dependent protein kinase OsDMI3 enhance OsCatB stability and activity to improve oxidative stress tolerance (Ni et al., 2022). The *M. oryzae* effector AvrPiz-t interacts with OsCatC to enhance its H_2O_2-scavenging activity; the rice E3 ligase OsAPIP6 counteracts this effect by targeting OsCatC for degradation, thereby restoring ROS accumulation (You et al., 2022). Despite these insights, how plants directly neutralize pathogen effectors that manipulate ROS homeostasis remains poorly understood.

Domain of unknown function 26 (DUF26) proteins, which are unique to land plants, have recently emerged as important regulators of extracellular signaling and stress responses (Chen, 2001; Vaattovaara et al., 2019). This family includes cysteine-rich receptor-like secreted proteins (CRRSPs), plasmodesmata-localized proteins (PDLPs), and cysteine-rich receptor-like kinases (CRKs). A defining feature of the extracellular DUF26 domain (Gnk2 or stress-antifungal domain, PF01657) is its conserved cysteine motif, characterized by the pattern C-8X-C-2X-C, which forms disulfide bonds and may mediate redox-dependent protein interactions (Chen, 2001). Several DUF26 proteins participate in immunity. The single-DUF26 domain protein Gnk2, derived from *Ginkgo biloba* nuts, exhibits potent antifungal activity *in vitro* against phytopathogenic fungi (*Fusarium oxysporum* and *Trichoderma reesei*) and the human pathogen *Candida albicans*, likely through carbohydrate-binding specificity resembling that of legume lectins (Sawano et al., 2007). The maize antifungal protein AFP1, which contains two DUF26 domains, increases fungal chitin levels by targeting chitin deacetylases and other glycoproteins (Ma et al., 2018). The cotton apoplastic protein GhCRR1 enhances resistance to *Verticillium dahliae* by stabilizing host chitinase 28 (Han et al., 2019). PDLP1 and PDLP5 promote defense by modulating callose deposition and salicylic acid signaling (Lee et al., 2011; Caillaud et al., 2014). AtCRK2 phosphorylates respiratory burst oxidase homolog D (RBOHD) to regulate ROS bursts, suggesting that DUF26 proteins can link extracellular perception with ROS production (Kimura et al., 2020). Numerous secreted DUF26 proteins have been identified *in silico*; however, the biochemical mechanisms by which CRRSPs modulate immunity remain largely unexplored, particularly in cereal crops.

Proteomic analyses of rice apoplastic secretions upon *M. oryzae* infection identified several DUF26-containing proteins that were also enriched in stress and ROS-related functions (Kim et al., 2013), including OsRMC (Os04g56430), a well-characterized apoplastic CRRSP that binds the conserved CBM1 motif of the fungal xylanase MoCel10A and restricts fungal invasion (Jiang et al., 2007; Zhang et al., 2009; Takeda et al., 2022). Another study demonstrated that OsRMC is essential for cell death mediated by the Nucleotide-binding, Apaf-1, R proteins, and Ced-4 (NB-ARC) class R protein OsRLS1 (rapid leaf senescence 1), as identified through suppressor screening (Wang et al., 2023). Despite these advances, the defense functions of most of the more than 20 CRRSPs in rice remain uncharacterized, leaving a substantial gap in our understanding of extracellular DUF26 protein function in immunity.

Here, we identify OsCRRSP1, a DUF26 domain-containing secreted protein, as a positive regulator of rice resistance to *M. oryzae*. We demonstrate that OsCRRSP1 interacts with both the pathogen-secreted CAT MoCatB and the rice CAT OsCatB, inhibiting their H_2O_2-scavenging activities. Through dual suppression of host and pathogen CATs, OsCRRSP1 sustains ROS accumulation and enhances immune activation. Given the central role of ROS in plant defense, we show that OsCRRSP1 also contributes to resistance against bacterial blight caused by *Xanthomonas oryzae* pv. *oryzae*, suggesting a role in broad-spectrum immunity. We propose that OsCRRSP1 functions as a molecular integrator linking extracellular effector neutralization with intracellular ROS-mediated defense, revealing a previously unrecognized mechanism of apoplastic immunity and providing new targets for engineering durable disease resistance in rice.

RESULTS

OsCRRSP1, a DUF26 domain-containing protein, is a positive regulator of rice blast resistance

In a previous study, we identified a group of "core" genes that were highly induced at 12, 24, 36, and 48 h after *M. oryzae* infection (Yang et al., 2021). To identify novel regulators of rice blast resistance, we prioritized approximately 30 candidate genes from this core set based on their strong and sustained induction and predicted functional relevance to immunity, including receptor-like kinases, receptor-like proteins, secreted proteins, kinases, and E3 ubiquitin ligases. We then performed a CRISPR-Cas9-based reverse genetic screen in the *japonica* rice cultivar Zhonghua 11 (ZH11); the resulting knockout mutants were evaluated for disease resistance. Among these candidates, the knockout mutant of *Os04g25060* (Figure 1A) exhibited significantly enhanced susceptibility, with larger disease lesions and higher fungal biomass than ZH11 after spray inoculation with *M. oryzae* strain Guy11 (Figures 1B and 1C). *Os04g25060* encodes a CRRSP containing two DUF26 domains and was designated *OsCRRSP1*. Punch inoculation assays confirmed the enhanced susceptibility of *Oscrrsp1* mutants to *M. oryzae* (Figures 1D and 1E). Consistent with this phenotype, defense marker genes, including *OsPBZ1*, *OsPAL*, *JIOsPR10*, and *OsPR3*, were substantially downregulated in the mutant (Supplemental Figure 1A); chitin-induced callose deposition was also significantly reduced (Supplemental Figures 1B and 1C). Furthermore, *OsCRRSP1* expression was strongly induced at 24 and 48 h post-inoculation (hpi) with *M. oryzae* (Figure 1F), consistent with its expression pattern in previous RNA sequencing data (Supplemental Figure 1D). Moreover, *OsCRRSP1* was ubiquitously expressed across various rice tissues, with significantly higher transcript levels in leaves than in other tissues (Figure 1G). Taken together, these results

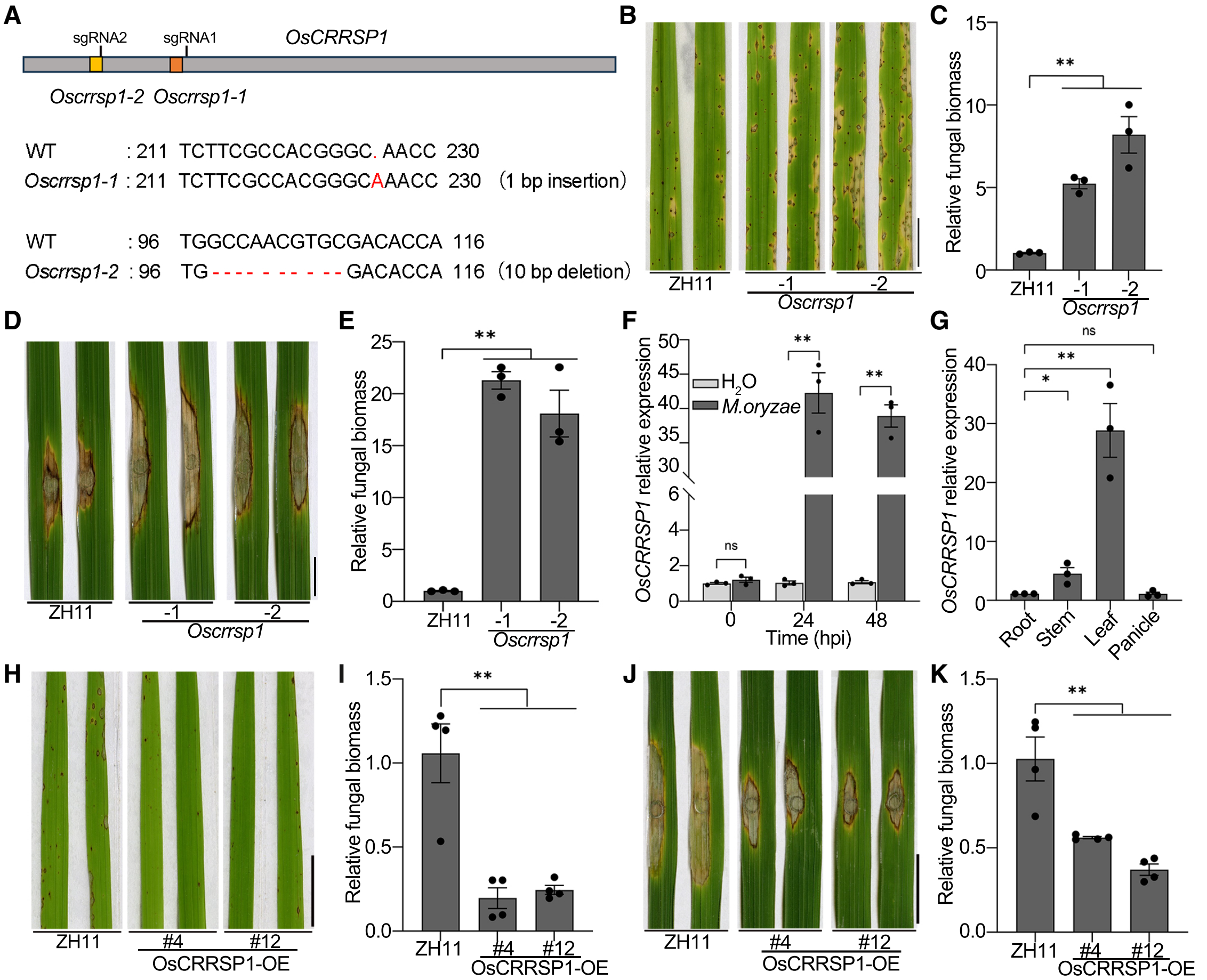

Figure 1. OsCRRSP1 positively regulates rice resistance to *M. oryzae*.

(A) Schematic of mutation sites in the *Oscrrsp1* knockout mutants (*Oscrrsp1*-1 and *Oscrrsp1*-2) generated by CRISPR-Cas9 in the ZH11 background. Inserted bases are highlighted in red, and red dotted lines indicate deleted nucleotides.

(B) Disease symptoms of 3-week-old wild-type ZH11 and *Oscrrsp1* mutant rice seedlings at 5 days after spray inoculation with *M. oryzae* strain Guy11 (2×10^5 conidia ml^{-1}). Scale bar, 1 cm.

(C) Relative fungal biomass in inoculated leaves shown in **(B)**, quantified by qPCR analysis of *M. oryzae Pot2* DNA and normalized to the rice *OsUBQ* reference gene. Data represent mean ± SE ($n = 3$). *$p < 0.05$, **$p < 0.01$ (Student's *t*-test vs. ZH11).

(D) Punch inoculation assay of leaves from 4-week-old ZH11 and *Oscrrsp1* mutant plants inoculated with Guy11 (1×10^5 conidia ml^{-1}). Photographs were taken 7 days post-inoculation. Scale bars, 1 cm.

(E) Relative fungal biomass corresponding to **(D)**, measured by qPCR. Data represent mean ± SE ($n = 3$). *$p < 0.05$, **$p < 0.01$ (Student's *t*-test vs. ZH11).

(F) Expression of *OsCRRSP1* in rice leaves at 24 and 48 h after inoculation with *M. oryzae* Guy11 or mock (H_2O) treatment. Data represent mean ± SE ($n = 3$). *$p < 0.05$, **$p < 0.01$ (Student's *t*-test vs. mock).

(G) Tissue-specific expression profile of *OsCRRSP1* in various rice organs, determined by RT–qPCR. Data represent mean ± SE ($n = 3$). ns, not significant. *$p < 0.05$, **$p < 0.01$ (Student's *t*-test vs. root).

(H) Disease symptoms of 3-week-old ZH11 and *OsCRRSP1*-OE transgenic rice seedlings at 5 days after spray inoculation with Guy11 (2×10^5 conidia ml^{-1}). Scale bars, 1 cm.

(I) Relative fungal biomass in leaves shown in **(H)**, determined by qPCR quantification of *M. oryzae Pot2* DNA relative to the rice *OsUBQ* gene. Data represent mean ± SE ($n = 3$). *$p < 0.05$, **$p < 0.01$ (Student's *t*-test vs. ZH11).

(J) Punch inoculation assay of leaves from 4-week-old ZH11 and *OsCRRSP1*-OE plants inoculated with Guy11 (1×10^5 conidia ml^{-1}). Representative images were taken 7 days post-inoculation. Scale bars, 1 cm.

(K) Relative fungal biomass corresponding to **(J)**. Data represent mean ± SE ($n = 4$). *$p < 0.05$, **$p < 0.01$ (Student's *t*-test vs. ZH11).

establish OsCRRSP1 as a key positive regulator of rice immunity against the rice blast fungus.

Overexpression of *OsCRRSP1* enhances blast resistance without penalizing growth

To further validate its function, we generated transgenic rice lines constitutively overexpressing *OsCRRSP1* fused to GFP (OsCRRSP1-OE) in the ZH11 background. RT–qPCR analysis revealed strongly elevated *OsCRRSP1* expression in these lines compared with the wild type (Supplemental Figure 1E). Western blot analysis using α-GFP antibodies detected the full-length OsCRRSP1-GFP fusion protein, confirming its successful accumulation (Supplemental Figure 1F). Neither the *Oscrrsp1* mutation nor *OsCRRSP1* overexpression affected plant growth or yield traits, including plant height, panicle seed-setting rate,

and thousand-grain weight (Supplemental Figures 2A–2H), suggesting that OsCRRSP1 acts specifically in defense without growth trade-offs.

Upon challenge with *M. oryzae*, *OsCRRSP1*-OE plants showed markedly enhanced resistance. Both spray and punch inoculation assays resulted in fewer and smaller lesions, along with significantly reduced fungal biomass, relative to ZH11 (Figures 1H–1K). This enhanced resistance was accompanied by elevated expression of the defense gene *OsPR3* (Supplemental Figure 2I) and increased chitin-induced callose deposition (Supplemental Figures 2J and 2K). These data demonstrated that *OsCRRSP1* positively regulates rice blast resistance.

Analysis of natural variation in *OsCRRSP1* identifies subspecies- and environment-associated haplotypes

Given the importance of *OsCRRSP1* in rice disease resistance, we investigated its natural variation using publicly available genomic datasets. Sliding-window analysis of nucleotide diversity (π) across a 40-kb region revealed no pronounced reduction within the *OsCRRSP1* coding region. Instead, moderate π values were observed at the *OsCRRSP1* locus, with upstream regions showing higher diversity and downstream regions showing lower diversity (Supplemental Figure 3A). Consistent with these findings, Tajima's D values across the coding region were close to zero or positive (Supplemental Figure 3B), suggesting neutral evolution or balancing selection; the strong negative downstream values indicated a nearby selective sweep rather than direct selection on *OsCRRSP1*.

Despite the absence of a clear selective sweep, *OsCRRSP1* retained multiple haplotypes across rice populations. After filtering, four polymorphic sites (one in the coding sequence [CDS] and three in the 3′ UTR) defined four major haplotypes: CGTG, CTAA, CTTG, and TTTG (Supplemental Table 1). CGTG was the predominant haplotype across subpopulations, particularly in *indica* varieties. In contrast, CTTG was substantially enriched in *japonica* varieties, whereas CTAA was mainly detected in the special *indica* group. TTTG showed moderate frequency in *indica* varieties but was rare in other groups (Supplemental Figure 3C). These patterns indicated clear subpopulation-specific differentiation.

Geographic analysis further revealed distinct spatial distributions of these haplotypes. CGTG was widely distributed in tropical and subtropical regions, whereas the *japonica*-enriched CTTG haplotype was predominantly found at higher latitudes corresponding to temperate regions. The CTAA haplotype exhibited a more restricted distribution (Supplemental Figure 3D). These results suggest that *OsCRRSP1* haplotypes exhibit spatial structuring and may be associated with environmental adaptation.

Although phenotypic associations with blast resistance have not been determined, the observed population structure and geographic patterns indicate that *OsCRRSP1* harbors persistent genetic variation with potential relevance to resistance breeding.

OsCRRSP1 is a secreted protein lacking direct antifungal activity

Bioinformatics analysis indicated that OsCRRSP1 is a 281-amino-acid protein characterized by an N-terminal signal peptide (SP) and two DUF26 domains, but lacks both transmembrane and intracellular kinase domains, consistent with its classification as a CRRSP rather than a receptor-like kinase (Supplemental Figure 4A) (Vaattovaara et al., 2019). Phylogenetic analysis of CRRSP proteins from rice, *Arabidopsis*, cotton, and maize showed that OsCRRSP1 grouped within a distinct clade, suggesting functional diversification within this family (Supplemental Figure 4B). Sequence alignment further indicated that OsCRRSP1 shares 59% identity with OsRMC and cotton GhCRR1/2/3/4; the two canonical DUF26 domains, defined by the C-8X-C-2X-C motif, were highly conserved (Supplemental Figure 4C).

To validate the functionality of the predicted SP of OsCRRSP1, we performed a yeast secretion assay using the YTK12 strain (Xu et al., 2019). The SP sequence was fused to the pSUC2 vector and introduced into yeast cells. Transformants expressing pSUC2-OsCRRSP1SP grew on YPRAA medium (1% yeast extract, 2% peptone, 2% raffinose, 2% agar, and 2 μg/ml antimycin A) containing raffinose as the sole carbon source (Figure 2A). Secretion activity was further confirmed by reduction of 2,3,5-triphenyltetrazolium chloride (TTC) to red triphenylformazan in an enzymatic assay (Figure 2A). These results demonstrated that OsCRRSP1 contains a functional signal peptide that mediates secretion.

To determine the subcellular localization of OsCRRSP1, we transiently co-expressed OsCRRSP1-YFP with either the plasma membrane marker mCherry-tagged *Arabidopsis* AtCNGC20 (Zhao et al., 2021) or the nuclear marker histone 3.1 in *Nicotiana benthamiana*. Prior to plasmolysis, OsCRRSP1 localized to both the plasma membrane and the cytoplasm (Figure 2B and Supplemental Figures 5A and 5B). After plasmolysis, a clear OsCRRSP1-YFP signal was observed in the apoplast between the plasma membrane and the cell wall (stained with FB-28; Figure 2B), confirming its secretion into the apoplast. Upon removal of the signal peptide, plasma membrane-associated fluorescence of OsCRRSP1 was significantly reduced, whereas cytoplasmic fluorescence increased (Supplemental Figures 5A and 5B), indicating that the signal peptide is essential for proper targeting. To further confirm localization in a homologous system, we examined OsCRRSP1-GFP in rice protoplasts derived from transgenic lines, revealing a similar plasma membrane and cytoplasmic localization pattern (Figure 2C). Furthermore, biochemical fractionation followed by immunoblot analysis confirmed that OsCRRSP1 is primarily associated with apoplastic and membrane fractions (Figures 2D and 2E), consistent with the imaging results.

Considering that some DUF26 proteins (e.g., Gnk2 and AFP1) possess direct antifungal activity (Sawano et al., 2007; Ma et al., 2018), we tested whether OsCRRSP1 has a similar function. However, purified glutathione S-transferase (GST)-OsCRRSP1 protein did not inhibit *M. oryzae* growth *in vitro* (Figure 2F), indicating that its defense function is not mediated through direct antifungal activity.

OsCRRSP1 interacts with the fungal catalase MoCatB

Given that OsCRRSP1 localizes to the apoplast but lacks direct antifungal activity, we hypothesized that its immune contribution

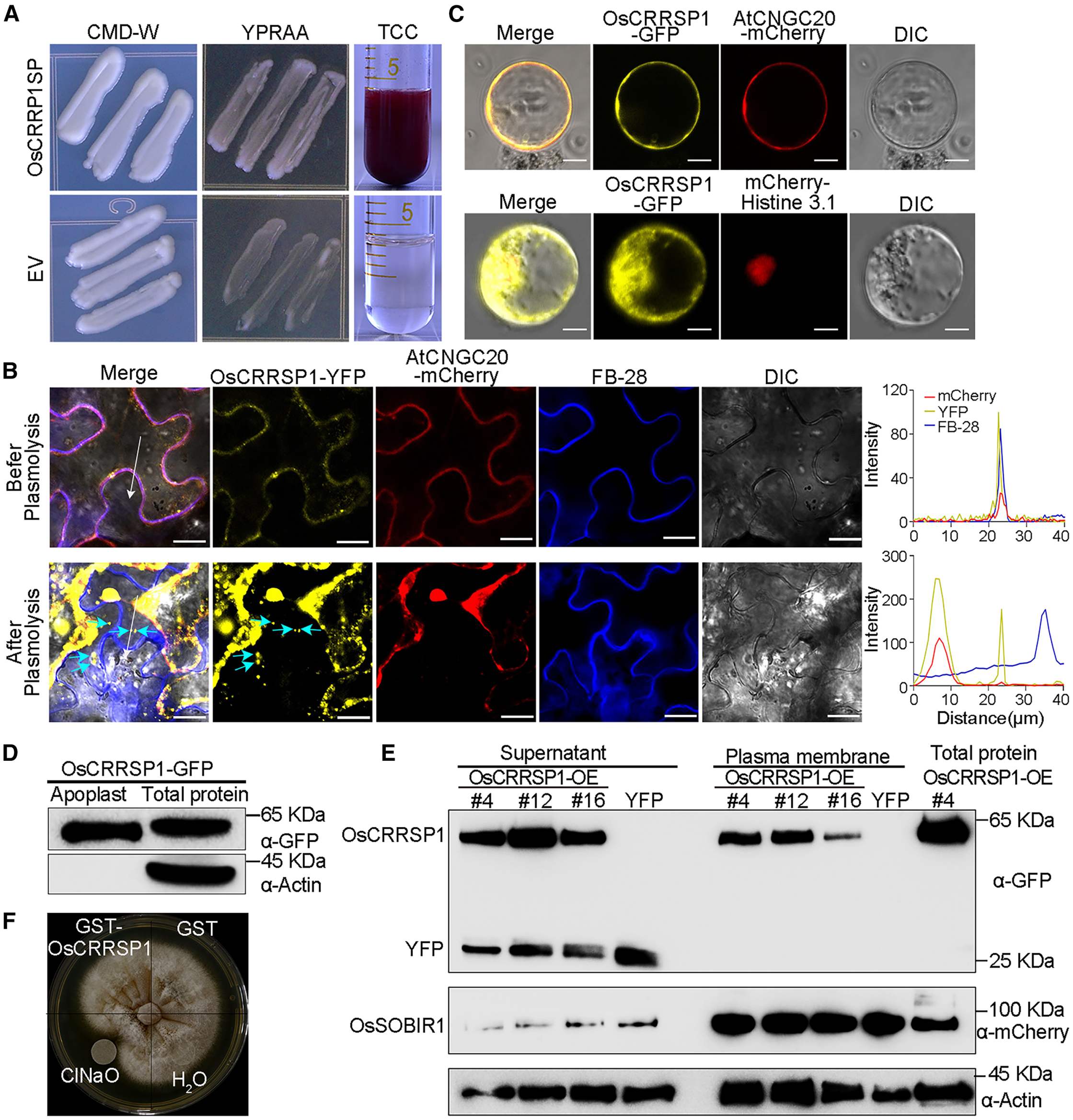

Figure 2. OsCRRSP1 is secreted into the apoplast and lacks direct antifungal activity.

(A) Functional validation of the OsCRRSP1 signal peptide (OsCRRSP1SP) using a yeast invertase secretion assay. *Saccharomyces cerevisiae strain* YTK12 expressing OsCRRSP1SP fused in-frame to invertase or the empty pSUC2 vector (negative control) was grown on CMD-W or YPRAA medium to assess invertase secretion. Secreted invertase activity was visualized by reduction of 2,3,5-triphenyltetrazolium chloride (TTC) to red formazan.

(B) OsCRRSP1 is located in the plant apoplast. Subcellular localization of OsCRRSP1-YFP (yellow) co-expressed with the plasma membrane marker AtCNGC20-mCherry (red) in *N. benthamiana* epidermal cells before and after plasmolysis induced by 1.5 M sorbitol. Images were acquired by confocal microscopy at 48 h post-agroinfiltration. Cell walls were stained with Fluorescent Brightener 28 (FB-28; blue). Blue arrows indicate OsCRRSP1-YFP signals in the apoplastic space after plasmolysis. Fluorescence intensity profiles were generated along the direction indicated by white arrows. Scale bars, 20 μm.

(C) Subcellular localization of OsCRRSP1-GFP in rice protoplasts. The plasma membrane marker AtCNGC20-mCherry (red) or nuclear marker mCherry-histone H3.1 (red) was expressed in OsCRRSP1-GFP-overexpressing protoplasts. Scale bar, 10 μm.

(D) Determination of OsCRRSP1 protein accumulation in the apoplastic space of leaves from T2-generation *OsCRRSP1*-OE seedlings. Rice actin served as a cytoplasmic protein control.

(E) Protein fractionation analysis using rice protoplasts isolated from *OsCRRSP1*-OE transgenic plants to determine the distribution of OsCRRSP1 in total protein, supernatant, and plasma membrane (PM) pellet fractions. The PM protein OsSOBIR1 served as a positive control for PM fractions, whereas YFP served as a negative control. Rice actin served as a loading control.

(F) *In vitro* antifungal activity assay of purified recombinant OsCRRSP1 protein against *M. oryzae*. Mycelial plugs of Guy11 were placed on complete medium (CM) agar plates surrounded by filter discs loaded with 20 μl of the following: purified GST-OsCRRSP1 (1 μg/μl), GST protein (1 μg/μl, EV control), sterile distilled water (blank control), or sodium hypochlorite (NaClO, fungicide control). Plates were incubated at 28°C for 6 days and photographed.

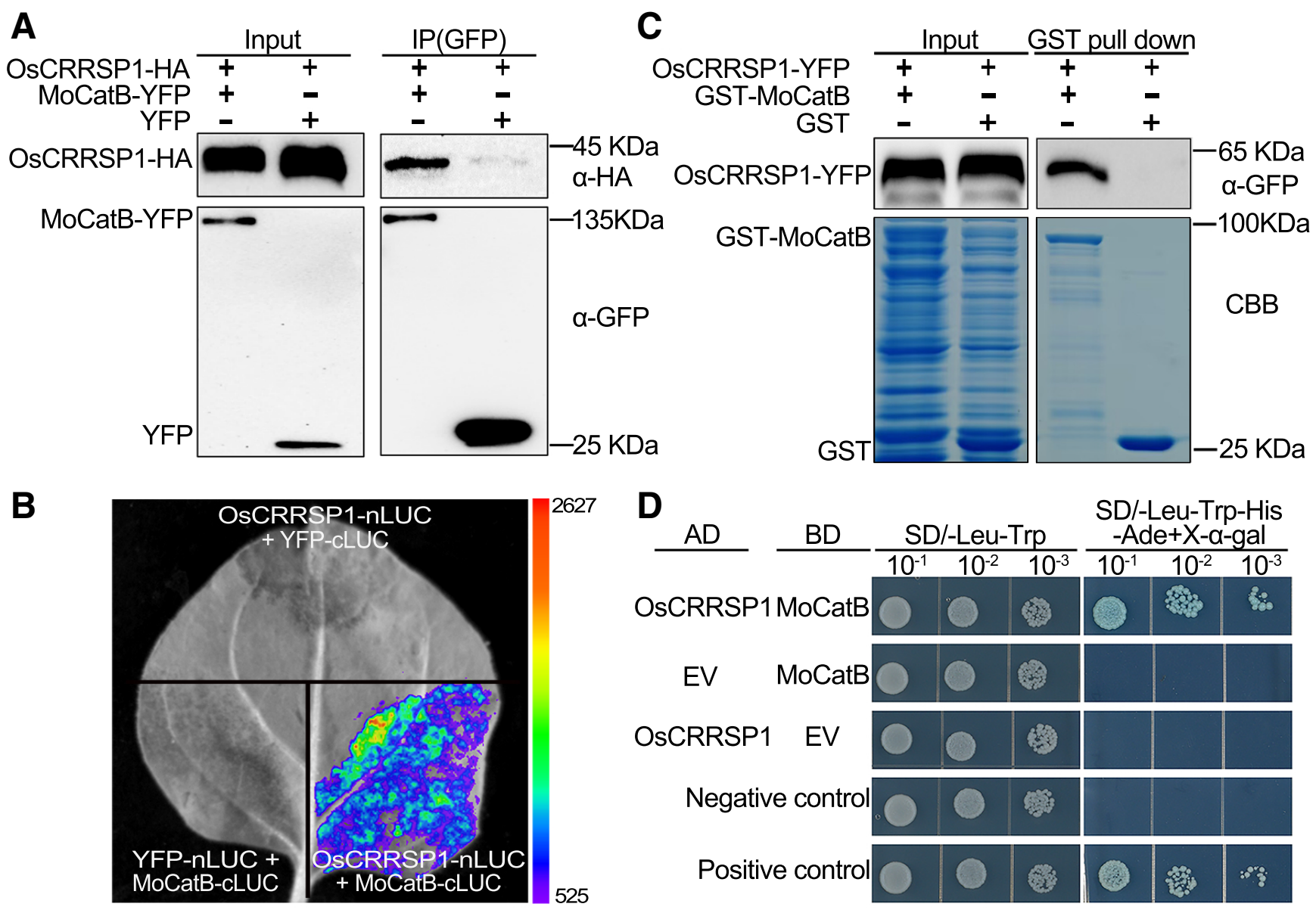

Figure 3. OsCRRSP1 associates with MoCatB.

(A) Co-immunoprecipitation (co-IP) assay testing the interaction between MoCatB and OsCRRSP1 *in planta*. Total proteins were extracted from rice protoplasts co-expressing MoCatB-YFP and OsCRRSP1-HA or control constructs (YFP and OsCRRSP1-HA). Proteins were immunoprecipitated using GFP-Trap beads and subsequently immunoblotted with the indicated antibodies. "Input" represents 2.5% of the total protein extract used for IP.

(B) Split-LUC complementation assay testing the interaction between OsCRRSP1-nLUC and MoCatB-cLUC in *N. benthamiana*. The indicated construct pairs were co-expressed, and LUC signals were imaged and quantified 48 h after infiltration. The OsCRRSP1-nLUC + cLUC and nLUC + MoCatB-cLUC combinations served as negative controls.

(C) GST pull-down assays demonstrating direct physical interaction between OsCRRSP1 and MoCatB. Recombinant GST-MoCatB or GST (control) immobilized on glutathione-Sepharose beads was incubated with purified OsCRRSP1-YFP. Bound proteins were eluted and analyzed by immunoblotting with an anti-GFP antibody (top) or by Coomassie Brilliant Blue (CBB) staining.

(D) Y2H assay confirming the interaction between OsCRRSP1 and MoCatB. Yeast cells expressing the indicated plasmids were grown on SD/−Leu−Trp plates and SD/−Leu−Trp−His−Ade plates (containing 40 mg/l X-α-gal) for 3 days. The AD-T + BD-53 pair served as a positive control; the combinations AD + BD, AD + BD-MoCatB, and AD-OsCRRSP1 + BD were used as negative controls.

involves interaction with fungal virulence factors. To identify *M. oryzae*-secreted proteins that interact with OsCRRSP1, we performed immunoprecipitation (IP) coupled with mass spectrometry (MS) using OsCRRSP1-YFP incubated with culture supernatant from the effector-rich *M. oryzae* mutant *ΔPoKmt6*, which lacks the methyltransferase PoKmt6 that suppresses the expression of secreted proteins through histone H3 lysine 27 trimethylation modification during vegetative growth (Xie et al., 2023). Among the candidate proteins identified (Supplemental Table 2), the CAT MoCatB (MGG_06442) emerged as a compelling candidate due to its known role in scavenging ROS, a central component of plant immunity. Previous research has shown that MoCatB is highly expressed during appressorium maturation and is required for full pathogenicity in *M. oryzae* (Skamnioti et al., 2007).

To validate this interaction, we used multiple independent assays. First, co-IP assays using transiently expressed proteins in rice protoplasts and *N. benthamiana* showed that OsCRRSP1-HA co-purified with MoCatB-YFP on α-GFP beads, demonstrating their association *in planta* (Figure 3A and Supplemental Figure 6A). Second, a split-luciferase (split-LUC) complementation assay confirmed this interaction in living plant cells (Figure 3B). Notably, deletion of the OsCRRSP1 signal peptide abolished this interaction (Supplemental Figure 6B), indicating that the interaction occurs specifically in the apoplast. To test for direct binding, we performed an *in vitro* pull-down assay using recombinant proteins expressed in *Escherichia coli*. OsCRRSP1-YFP was specifically pulled down by GST-MoCatB, but not by GST alone (Figure 3C). Finally, a yeast two-hybrid (Y2H) assay showed that yeast co-expressing OsCRRSP1 and MoCatB grew on synthetic dropout (SD) medium (SD/−Trp/−Leu/−His/−Ade) (Figure 3D), confirming direct protein–protein interaction. To further determine the interaction interface, MoCatB was divided into three fragments based on its CAT core (Cc) domain: MoCatB-N (1–53), MoCatB-Cc (54–440), and MoCatB-C (441–715) (Supplemental Figure 6C). Y2H and co-IP assays were then used to examine whether OsCRRSP1 interacted with these three truncated MoCatB fragments. Both methods showed that OsCRRSP1 associates with MoCatB through multiple regions, including the Cc domain and both the N- and C-terminal regions (Supplemental Figures 6D–6F). Taken together, these results demonstrate that OsCRRSP1 directly interacts with MoCatB.

MoCatB is a secreted apoplastic protein that is required for full pathogenicity of *M. oryzae*

For the OsCRRSP1–MoCatB interaction to occur during infection, MoCatB must be secreted into host tissues. Bioinformatic analysis predicted an N-terminal signal peptide (SP) in MoCatB. A similar yeast secretion assay confirmed the functionality of this SP (Supplemental Figure 7A). To validate secretion in *M. oryzae*, we generated a strain expressing MoCatB-GFP and detected the protein in both mycelia and culture supernatants, confirming active secretion (Supplemental Figure 7B). When transiently expressed in *N. benthamiana*, MoCatB-YFP was observed in the apoplastic space between the plasma membrane and cell wall after plasmolysis (Supplemental Figure 7C). To determine its localization during infection, we generated a Guy11 strain co-expressing MoCatB-GFP and the cytoplasmic effector Pwl2-mCherry (Giraldo et al., 2013). During infection, MoCatB localized to the periphery of invasive hyphae (IHs), exhibiting a localization pattern distinct from that of the cytoplasmic effector Pwl2, which accumulates at the

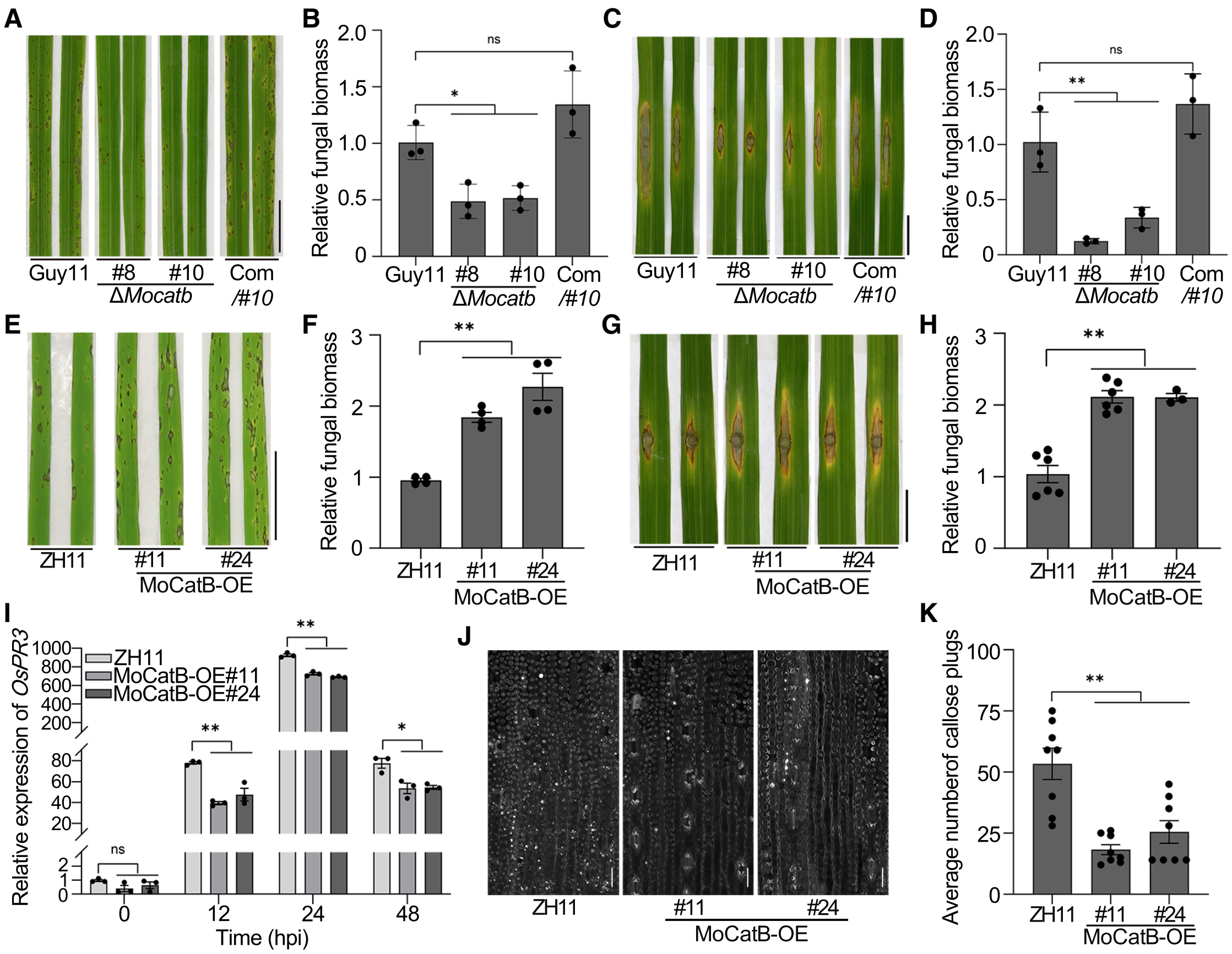

Figure 4. MoCatB is required for *M. oryzae* virulence, and its ectopic expression in rice suppresses immunity.
(A) Disease symptoms of 3-week-old rice seedlings at 5 days after spray inoculation with wild-type Guy11, *ΔMocatb* mutants (#8, #10), or the complemented strain (Com/#10). Scale bars, 1 cm.
(B) Relative fungal biomass in leaves from **(A)**, quantified by qPCR analysis of the *M. oryzae Pot2* gene normalized to rice *OsUBQ*. Data represent mean ± SE ($n = 3$). *$p < 0.05$ (Student's *t*-test vs. Guy11).
(C) Punch inoculation assay of leaves from 4-week-old plants inoculated with wild-type Guy11, Δ*Mocatb* mutants (#8, #10), or the complemented strain (Com/#10). Lesions were photographed at 7 days post-inoculation. Scale bars, 1 cm.
(D) Relative fungal biomass corresponding to **(C)**. Data represent mean ± SE ($n = 3$). **$p < 0.01$ (Student's *t*-test vs. Guy11).
(E) Disease symptoms of 3-week-old wild-type (ZH11) and *MoCatB*-OE transgenic seedlings at 5 days post-inoculation with Guy11. Scale bars, 1 cm.
(F) Relative fungal biomass in leaves from **(E)**. Data represent mean ± SE ($n = 4$). **$p < 0.01$ (Student's *t*-test vs. ZH11).
(G) Punch inoculation assay of leaves from 4-week-old ZH11 and *MoCatB*-OE plants inoculated with Guy11. Representative images were taken 7 days post-inoculation. Scale bars, 1 cm.
(H) Relative fungal biomass corresponding to **(G)**. Data represent mean ± SE ($n = 6$). **$p < 0.01$ (Student's *t*-test vs. ZH11).
(I) Expression of the *OsPR3* gene in ZH11 and *MoCatB*-OE plants before and after *M. oryzae* infection, analyzed by RT−qPCR and normalized to *OsUBQ*. Data represent mean ± SE ($n = 3$). *$p < 0.05$, **$p < 0.01$ (Student's *t*-test vs. ZH11).
(J) Callose deposition in leaves of ZH11 and *MoCatB*-OE plants after chitin elicitation (800 nM), visualized by aniline blue staining. Scale bars, 50 μm.
(K) Quantification of callose deposits shown in **(J)** using ImageJ software. Data represent mean ± SE ($n = 6$). *$p < 0.05$, **$p < 0.01$ (Student's *t*-test vs. ZH11).

biotrophic interfacial complex (Supplemental Figure 7D). These results suggest that MoCatB functions as a secreted apoplastic effector.

To assess the role of MoCatB in fungal virulence, we generated *MoCatB* knockout mutants (Δ*Mocatb* #8 and #10) in the Guy11 background (Supplemental Figures 8A–8D) and a complemented strain (Com/#10) expressing MoCatB-GFP (Supplemental Figure 8E). The Δ*Mocatb* mutants exhibited no defects in growth rate or morphology on complete medium (CM) or rice bran medium (Supplemental Figures 9A–9D). Additionally, no abnormalities were observed in conidial germination or appressorium formation (Supplemental Figure 9E), indicating that MoCatB is dispensable for normal fungal development. Pathogenicity assays, however, revealed that both Δ*Mocatb* mutants exhibited markedly attenuated virulence, as indicated by reduced lesion size and fungal biomass in both spray- and punch-inoculated rice plants. Virulence was fully restored in the complemented strain Com/#10 (Figures 4A–4D).

We next analyzed IH development in rice sheath cells. Based on morphological progression, IHs were classified into 4 types: type 1, filamentous primary IHs; type 2, a single bulbous IH; type 3, branched bulbous IHs; and type 4, IHs that fully occupy a plant

cell and penetrate neighboring cells (Supplemental Figure 9F). At 24 and 48 hpi, Δ*Mocatb* mutants were largely restricted to type 1 (filamentous) and type 2 (bulbous) IHs, whereas Guy11 and Com/#10 frequently developed type 3 (branched) and type 4 (neighbor-penetrating) IHs (Supplemental Figure 9G), indicating that MoCatB is required for IH development. Consistent with its role as a CAT, 3,3′-diaminobenzidine (DAB) staining revealed stronger H_2O_2 accumulation in plants infected with Δ*Mocatb* mutants than in those infected with Guy11 or Com/#10 (Supplemental Figure 9H). Taken together, these results demonstrate that MoCatB functions as a secreted virulence factor that disrupts host ROS homeostasis to promote infection.

Ectopic expression of MoCatB attenuates rice resistance to *M. oryzae*

MoCatB is a fungal homolog of the rice CAT OsCatB, which negatively regulates rice immunity by degrading H_2O_2 (Gao et al., 2021). To test whether MoCatB similarly suppresses host defense, we generated transgenic rice lines constitutively expressing *MoCatB-GFP* under the 35S promoter (*MoCatB*-OE) (Supplemental Figure 10A). These lines developed normally under greenhouse conditions (Supplemental Figures 10B–10H). However, after spray inoculation with *M. oryzae*, *MoCatB*-OE plants exhibited significantly more and larger lesions than ZH11 controls (Figure 4E); they also supported greater fungal biomass (Figure 4F). Punch inoculation assays similarly showed expanded lesions and increased fungal proliferation in *MoCatB*-OE plants (Figures 4G and 4H). Consistent with enhanced susceptibility, expression of the defense marker gene *OsPR3* was significantly reduced (Figure 4I), and chitin-induced callose deposition was considerably impaired in *MoCatB*-OE lines (Figures 4J and 4K). These results demonstrate that heterologous expression of *MoCatB* in rice compromises blast resistance, consistent with its function as an H_2O_2-degrading enzyme.

OsCRRSP1 interacts with the rice endogenous catalase OsCatB

To identify endogenous interactors of OsCRRSP1, we performed a Y2H screen of a rice cDNA library using OsCRRSP1 as bait, in parallel with our search for fungal targets. Intriguingly, this screen identified OsCatB and OsCatC as potential interactors (Supplemental Table 3). Because the rice genome encodes three CATs (OsCatA, OsCatB, and OsCatC), we evaluated each for interaction with OsCRRSP1 using Y2H assays. Yeast cells co-expressing OsCRRSP1 with any of these three OsCat proteins grew on selective medium (Figure 5A and Supplemental Figure 11A), suggesting OsCRRSP-OsCat interactions in yeast.

To verify these interactions, we performed an *in vitro* pull-down assay with recombinant proteins. OsCRRSP1-YFP was specifically co-purified with GST-OsCatB, but not with GST-OsCatA or GST-OsCatC (Figure 5B and Supplemental Figure 11B), indicating a direct and specific interaction with OsCatB. This specificity was confirmed *in planta* by co-IP assays using rice protoplasts and *N. benthamiana*; OsCRRSP1-HA co-purified with OsCatB-YFP, but not with OsCatA or OsCatC (Figure 5C and Supplemental Figures 11C–11E). A split-LUC assay confirmed the association between OsCRRSP1 and OsCatB *in vivo* (Figure 5D). Importantly, OsCRRSP1 and OsCatB colocalized at the plasma membrane and in the cytoplasm (Figure 5E). Furthermore, *OsCatB* expression was significantly induced at 24 and 48 hpi with *M. oryzae* (Figure 5F), displaying an induction pattern similar to that of *OsCRRSP1* (Figure 1F). These results demonstrate that OsCRRSP1 specifically interacts with OsCatB both *in vitro* and *in vivo*, suggesting that this interaction is a key regulatory node in rice immune signaling during fungal infection.

OsCRRSP1 promotes pathogen-induced ROS accumulation by inhibiting catalase activity

Given our findings that MoCatB promotes *M. oryzae* virulence and that OsCRRSP1 interacts with both MoCatB and OsCatB, we hypothesized that OsCRRSP1 enhances immunity by suppressing the H_2O_2-degrading activity of these CATs. To determine whether MoCatB suppresses host ROS accumulation, we measured H_2O_2 levels in *MoCatB*-OE plants after infection. DAB staining revealed significantly lower H_2O_2 accumulation in *MoCatB*-OE plants than in ZH11 following inoculation with *M. oryzae* (Supplemental Figures 12A and 12B). Consistent with this finding, *MoCatB*-OE plants also exhibited an attenuated ROS burst in response to chitin elicitation (Supplemental Figure 12C), confirming that MoCatB suppresses ROS-dependent defense in rice. Conversely, *OsCRRSP1*-OE plants accumulated higher levels of H_2O_2 (Supplemental Figures 12D and 12E) and exhibited an enhanced chitin-induced ROS burst relative to ZH11 (Supplemental Figure 12F).

In contrast, both pathogen-induced H_2O_2 accumulation and elicitor-induced ROS production were significantly reduced in *Oscrrsp1* mutants (Figures 6A–6C), indicating that OsCRRSP1 is required for full ROS induction. To determine whether this reduction results from impaired ROS production, we analyzed the expression of key nicotinamide adenine dinucleotide phosphate hydrogen (*NADPH*) *oxidase* genes (*OsRBOHA*, *OsRBOHB*, and *OsRBOHI*), which are involved in immune-associated ROS generation (Li et al., 2022; Zhao et al., 2024; Zhu et al., 2024). Under noninfected conditions, no significant differences in gene expression were observed between *Oscrrsp1* mutants and wild-type plants (Supplemental Figures 12G–12I, 0 hpi). After *M. oryzae* inoculation, moderate and gene-specific changes were detected at 24 hpi; *OsRBOHB* showed increased expression, whereas *OsRBOHA* and *OsRBOHI* exhibited reduced induction in the mutant (Supplemental Figures 12G–12I). However, these differences were not sustained over time; by 48 hpi, expression levels largely converged (Supplemental Figures 12G–12I).

Importantly, the chitin-induced ROS bursts analyzed here occur within minutes (Figure 6C), a timeframe unlikely to involve substantial transcriptional regulation of *RBOH* genes. Therefore, the reduced ROS accumulation observed in *Oscrrsp1* mutants is unlikely to result from altered ROS production at the transcriptional level. Instead, these findings suggest that OsCRRSP1 primarily regulates ROS homeostasis by modulating ROS scavenging.

Given that OsCRRSP1 interacts with both OsCatB and MoCatB, we next examined whether it directly affects their enzymatic activity. Using a transit expression system in *Oscrrsp1* mutant rice protoplasts, we measured CAT activity. Compared with YFP-HA, both OsCatB-YFP and MoCatB-YFP co-expressed with OsCRRSP1-YFP exhibited significantly reduced

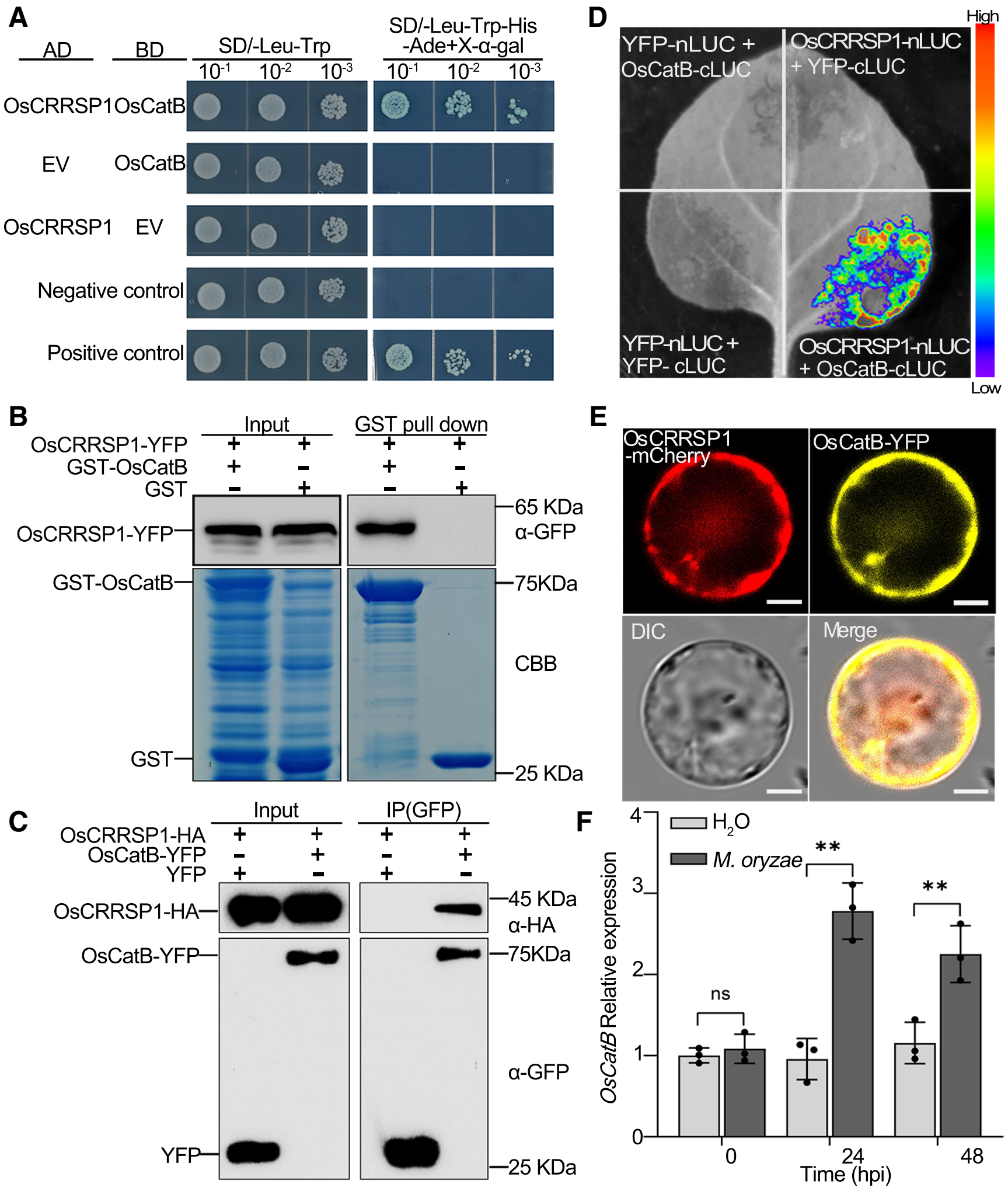

Figure 5. OsCRRSP1 interacts with the rice catalase OsCatB both *in vivo* and *in vitro*.

(A) Y2H assay showing the interaction between OsCRRSP1 and OsCatB. Yeast cells co-expressing the indicated bait (BD) and prey (AD) constructs were grown on SD/−Leu−Trp plates or on SD/−Leu−Trp−His−Ade plates (containing 40 mg/l X-α-gal). The AD-T + BD-53 pair served as a positive control; AD + BD, AD + BD-OsCatB, and AD-OsCRRSP1 + BD were used as negative controls. Plates were incubated at 30°C for 3 days.

(B) GST pull-down assay demonstrating direct interaction between OsCRRSP1 and OsCatB. Recombinant GST-OsCatB or GST (control) immobilized on glutathione-Sepharose beads was incubated with purified OsCRRSP1-YFP. Bound proteins were analyzed by immunoblotting using an anti-GFP antibody (top). The bottom panel shows CBB staining of the proteins.

(C) Co-IP assay confirming the interaction between OsCRRSP1 and OsCatB in rice protoplasts. Protoplasts isolated from rice seedlings were co-transfected with constructs expressing OsCRRSP1-HA and OsCatB-YFP or the control construct YFP. Proteins were immunoprecipitated using GFP-Trap beads and immunoblotted with the indicated antibodies. Input represents 2.5% of the total protein extract.

(D) Split-LUC complementation assay in *N. benthamiana* leaves. OsCRRSP1 was fused to nLUC, and OsCatB was fused to cLUC. The indicated construct pairs were co-expressed, and luciferase activity was imaged at 48 h post-infiltration. The YFP-nLUC + OsCatB-cLUC, OsCRRSP1-nLUC + YFP-cLUC, and YFP-nLUC + YFP-cLUC combinations served as negative controls.

(E) Colocalization of OsCRRSP1-YFP and OsCatB-mCherry in rice protoplasts. OsCRRSP1-YFP (yellow) and OsCatB-mCherry (red) showed overlapping localization at the cell periphery and in the cytoplasm. 35S-YFP served as a cytosolic and nuclear marker control. Images were acquired by confocal microscopy. Scale bars, 5 μm.

(F) Expression of *OsCatB* in rice leaves inoculated with *M. oryzae* Guy11 or mock treated with H_2O. Samples were collected at the indicated time points and analyzed by RT–qPCR normalized to *OsUBQ*. Data represent mean ± SE ($n = 3$). $^{**}p < 0.01$ (Student's t-test vs. H_2O).

H_2O_2 degradation activity (Figures 6D and 6E), indicating that OsCRRSP1 suppresses their CAT activity. Additionally, total CAT activity was approximately 2-fold higher in infected leaves of *Oscrrsp1* mutants than in ZH11 (Figure 6F). Overexpression of *MoCatB* increased total CAT activity by approximately 28% (Figure 6G), whereas *OsCRRSP1*-OE plants showed more than a 53% reduction in CAT activity compared with the wild type (Figure 6H). These results demonstrate that OsCRRSP1 enhances the defense-related ROS burst by directly inhibiting the H_2O_2-scavenging activities of both MoCatB and OsCatB.

OsCRRSP1 contributes to rice resistance against bacterial blight

Previous studies have established that several DUF26-containing proteins, such as OsRMC, OsCRK6, and OsCRK10, contribute to defense against the bacterium *X. oryzae pv. oryzae* (Chern et al., 2016; Wang et al., 2023). Given the central role of ROS signaling in plant immunity and the function of OsCRRSP1 in regulating ROS homeostasis, we hypothesized that OsCRRSP1 contributes to resistance against other pathogens, such as *X. oryzae pv. oryzae*. To test this hypothesis, we first examined the ROS burst induced by flg22 (a conserved peptide derived from bacterial flagellin) and found that *Oscrrsp1* mutants exhibited a significantly reduced oxidative burst relative to ZH11 (Figure 7A). Furthermore, the expression pattern of *OsCRRSP1* during *X. oryzae pv. oryzae* infection mirrored its induction in response to the blast fungus *M. oryzae*. Notably, *OsCRRSP1* was rapidly induced during the early stage of infection, and then gradually declined during disease progression (Supplemental Figure 12J), suggesting a role for OsCRRSP1 in rice defense against bacterial pathogens. We then evaluated resistance to *X. oryzae pv. oryzae* through field inoculation assays. The *Oscrrsp1* mutants developed significantly longer lesions, approximately 74% greater than those of ZH11 (Figures 7B and 7C), whereas *OsCRRSP1*-OE plants developed lesions that were 21.8% shorter than those of the wild type (Figures 7D and 7E). Moreover, *MoCatB*-OE plants displayed enhanced susceptibility to *X. oryzae pv. oryzae* (Figures 7F and 7G), consistent with its role in H_2O_2 degradation. Furthermore,

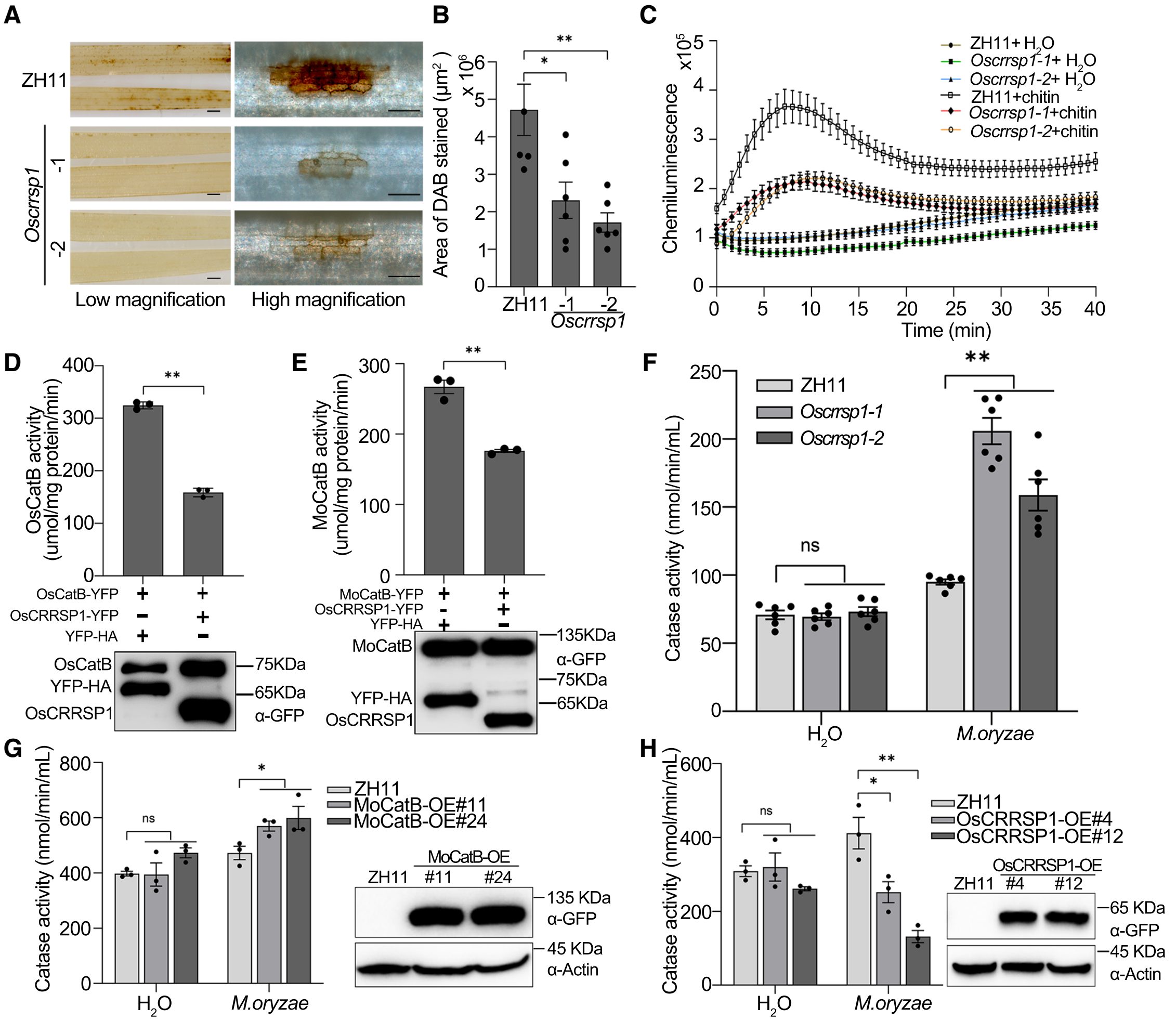

Figure 6. OsCRRSP1 promotes pathogen-induced ROS accumulation by inhibiting catalase activity.
(A) DAB staining detecting H_2O_2 accumulation in ZH11 and *Oscrrsp1* mutants at 48 h post-inoculation with Guy11. Scale bars, 1 cm (top) and 500 μm (bottom).
(B) Quantification of DAB-stained areas shown in **(A)**. Data represent mean ± SE ($n = 6$). *$p < 0.05$, **$p < 0.01$ (Student's *t*-test vs. ZH11).
(C) Chitin-induced ROS burst in leaf discs from ZH11 and *Oscrrsp1 plants* treated with 800 nM chitin. Data represent mean ± SE ($n = 10$).
(D) OsCRRSP1 inhibits the H_2O_2-degrading activity of OsCatB. OsCatB-YFP fusion proteins were extracted from *Oscrrsp1* mutant protoplasts. OsCatB-YFP and OsCRRSP1-YFP fusion proteins, along with YFP-HA as a control, were purified by IP using an anti-GFP antibody. Data represent mean ± SE ($n = 3$). **$p < 0.01$ (Student's *t*-test).
(E) OsCRRSP1 inhibits the H_2O_2-degrading activity of MoCatB. MoCatB-YFP fusion proteins were extracted from *Oscrrsp1* mutant protoplasts. MoCatB-YFP and OsCRRSP1-YFP fusion proteins, along with YFP-HA as a control, were purified by IP using an anti-GFP antibody. Data represent mean ± SE ($n = 3$). **$p < 0.01$ (Student's *t*-test).
(F) Total CAT activity in protein extracts from ZH11 and *Oscrrsp1* plants with or without Guy11 inoculation. Data represent mean ± SE ($n = 6$). *$p < 0.05$, **$p < 0.01$ (Student's *t*-test vs. ZH11).
(G) Total CAT activity in protein extracts from ZH11 and *MoCatB*-OE plants with or without Guy11 inoculation. Western blot analysis of MoCatB-GFP expression in *MoCatB*-OE and wild-type plants using an anti-GFP antibody. Actin served as a loading control. Data represent mean ± SE ($n = 3$). *$p < 0.05$, **$p < 0.01$ (Student's *t*-test vs. ZH11).
(H) Total CAT activity in protein extracts from ZH11 and *OsCRRSP1*-OE plants with or without Guy11 inoculation. Western blot detection of OsCRRSP1-GFP expression in *OsCRRSP1*-OE and wild-type plants using an anti-GFP antibody. Actin served as a loading control. Data represent mean ± SE ($n = 3$). *$p < 0.05$, **$p < 0.01$ (Student's *t*-test vs. ZH11).

the flg22-induced ROS accumulation patterns observed in *OsCRRSP1*-OE and *MoCatB*-OE plants were positively correlated with their resistance phenotypes after *X. oryzae pv. oryzae* infection (Figures 7H and 7I). This finding demonstrates that disease resistance in these transgenic plants is closely associated with ROS levels. These results indicate that OsCRRSP1 positively regulates resistance to bacterial blight. Collectively, our findings identify OsCRRSP1 as an important regulator of broad-spectrum rice resistance against both fungal and bacterial pathogens.

DISCUSSION

The evolutionary arms race between plants and pathogens has driven the development of sophisticated defense and

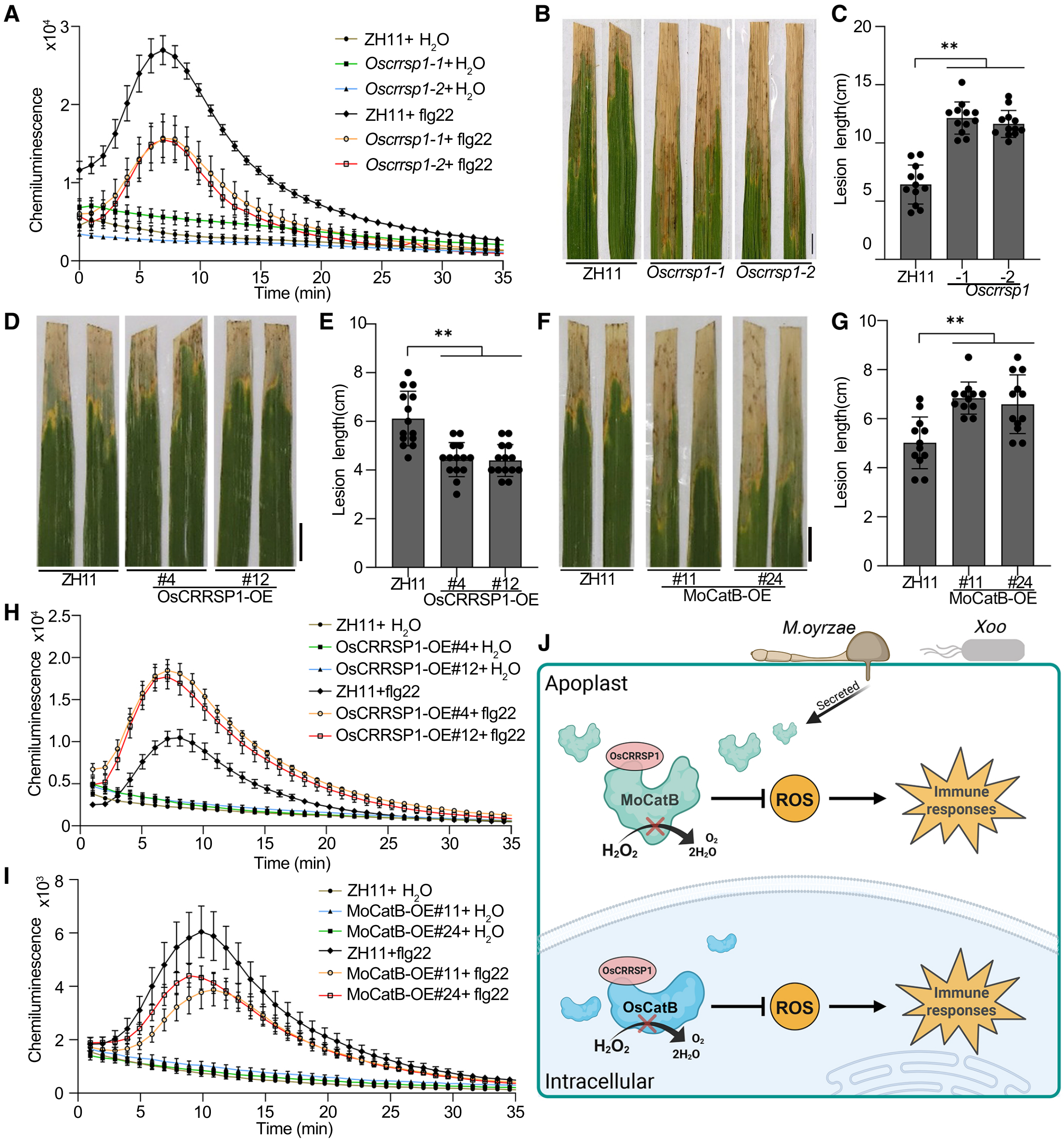

Figure 7. OsCRRSP1 enhances resistance to bacterial blight in rice.

(A) flg22-induced ROS burst in leaf discs from ZH11 and *Oscrrsp1* mutants treated with 500 nM flg22. Luminescence was measured over 35 min. Data represent means ± SE (n = 10).

(B) Bacterial blight symptoms on leaves of ZH11 and *Oscrrsp1* mutants at 14 days after clip inoculation with *X. oryzae* pv. *oryzae* strain PXO99.

(C) Quantification of lesion lengths from **(B)**. Data represent means ± SE (n = 10). *$p < 0.05$, **$p < 0.01$ (Student's t-test vs. ZH11).

(D) Bacterial blight symptoms on leaves of ZH11 and *OsCRRSP1*-OE plants at 14 days after clip inoculation with the *X. oryzae pv. oryzae* strain PXO99.

(E) Quantification of lesion lengths from **(D)**. Data represent means ± SE (n = 14). *$p < 0.05$, **$p < 0.01$ (Student's t-test vs. ZH11).

(F) Bacterial blight symptoms on leaves of ZH11 and *MoCatB*-OE plants at 14 days after clop inoculation with *X. oryzae pv. oryzae* strain PXO99.

(G) Quantification of lesion lengths from **(F)**. Data represent means ± SE (n = 12). *$p < 0.05$, **$p < 0.01$ (Student's t-test vs. ZH11).

(H) flg22-induced ROS burst in leaf discs from ZH11 and *OsCRRSP1*-OE plants treated with 500 nM flg22. Data represent means ± SE (n = 8).

(I) flg22-induced ROS burst in leaf discs from ZH11 and *MoCatB*-OE plants treated with 500 nM flg22. Data represent means ± SE (n = 8).

(J) Proposed working model illustrating OsCRRSP1-mediated inhibition of MoCatB and OsCatB CAT activity to enhance rice resistance.

In wild-type rice, *M*. *oryzae* infection induces *OsCRRSP1* expression, and the secreted OsCRRSP1 protein localizes to the apoplast, where it directly interacts with and inhibits the CAT activity of the fungal virulence factor MoCatB, thereby preventing apoplastic ROS scavenging. Concurrently, OsCRRSP1 interacts with and suppresses the activity of the host intracellular CAT OsCatB. This dual inhibition elevates ROS levels in both the apoplast and cytoplasm, potentiating rice immune responses and enhancing blast resistance. In *Oscrrsp1* knockout lines, failure to suppress MoCatB or OsCatB activity permits efficient ROS scavenging and increases disease susceptibility. Conversely, overexpression of *OsCRRSP1* strengthens inhibition of both CATs, leading to robust ROS accumulation and substantially enhanced blast resistance compared with wild-type plants.

counter-defense strategies (Wang et al., 2022). Although numerous resistance genes against rice blast have been identified, most have been rapidly overcome through pathogen evolution (Dean et al., 2012; Li et al., 2020), highlighting the need for novel resistance mechanisms with greater durability. Here, we unveil a previously unrecognized regulatory module centered on OsCRRSP1, a DUF26-containing secreted protein that utilizes a dual-targeting strategy to coordinate ROS-mediated immunity. OsCRRSP1 directly interacts with and suppresses the H_2O_2-degrading activities of both the fungal CAT MoCatB and the rice CAT OsCatB, thereby enhancing ROS accumulation across cellular compartments and reinforcing immune responses (Figure 7J).

Our work expands the known functional spectrum of DUF26 domain-containing proteins. Although CRRSPs have been implicated in fungal defense, their mechanisms vary considerably. For example, cotton GhCRR1 enhances resistance by protecting host chitinases from pathogen-derived proteases, thus stabilizing key defense enzymes (Han et al., 2019). In contrast, rice OsRMC (Takeda et al., 2022) and maize ZmAFP1 (Ma et al., 2018) directly target and inhibit fungal cell wall-degrading enzymes. Whereas these strategies primarily rely on protein stabilization, inhibition of pathogen hydrolases, or direct antimicrobial activity, OsCRRSP1 adopts a distinct dual-targeting mechanism by simultaneously interacting with both pathogen-derived (MoCatB) and host-derived (OsCatB) CATs. Rather than directly attacking the pathogen or protecting host proteins, OsCRRSP1 modulates the redox environment by suppressing ROS-scavenging activity and thus enhancing immune-associated ROS accumulation. This mechanism reflects a conceptual shift from direct antimicrobial or protective functions toward the regulation of host–pathogen metabolic balance, highlighting the evolutionary flexibility of the DUF26 domain as a protein interaction module adapted to counter diverse virulence strategies.

Notably, there is increasing recognition of the involvement of DUF26 proteins in ROS regulation across species. *Arabidopsis* AtCRK2 regulates ROS production through RBOHD phosphorylation (Kimura et al., 2020), whereas AtCRK6/7 provide protection during oxidative stress (Idänheimo et al., 2014). However, OsCRRSP1 is the first DUF26 protein shown to directly inhibit CAT activity, suggesting convergent evolution toward ROS modulation through distinct molecular mechanisms. The conserved cysteine motifs characteristic of DUF26 domains may facilitate redox-sensitive interactions (Chen, 2001; Vaattovaara et al., 2019), potentially explaining their recurrent involvement in oxidative stress responses.

Our findings reveal a sophisticated two-tiered defense strategy operating across cellular compartments. In the apoplast, OsCRRSP1 neutralizes MoCatB, a dedicated virulence effector that represents the pathogen's countermeasure against host-derived ROS. MoCatB's localization to the apoplast (Supplemental Figure 7), its essential role in pathogenicity (Figure 4), and its ability to suppress host ROS (Figure 6 and Supplemental Figure 12) collectively establish it as a key virulence determinant. This finding supports the growing recognition that pathogens actively manipulate host redox environments. For example, the *M. oryzae* effector AvrPiz-t activates rice OsCatC to enhance H_2O_2 removal and promote infection (You et al., 2022). Similarly, the wheat stripe rust pathogen *Puccinia striiformis* f.sp. *tritici*-secreted effector Pst9653 targets wheat TaCAT3, increasing its cytoplasmic abundance to inhibit ROS accumulation and weaken host resistance (Wei et al., 2025).

More importantly, OsCRRSP1 concurrently targets the host's intrinsic ROS regulator, OsCatB (Figure 5; Supplemental Figure 11). This dual inhibition creates a powerful amplification system—by suppressing both the pathogen's ROS detoxification machinery and the host's primary negative regulator, OsCRRSP1 ensures robust ROS accumulation across multiple cellular compartments. This coordinated regulation may account for the particularly strong resistance phenotype observed in the overexpression lines and differs substantially from previously reported regulatory mechanisms, such as ROD1-mediated activation of OsCatB (Gao et al., 2021).

The conservation of ROS as a defense signal across pathogen groups may explain OsCRRSP1-mediated resistance against both fungal and bacterial pathogens. Our demonstration that OsCRRSP1 enhances resistance to *M. oryzae* and *X. oryzae pv. oryzae* without yield penalties addresses a major challenge in resistance breeding. The correlation between flg22-induced ROS bursts and disease resistance across transgenic lines provides functional support for this mechanism and suggests that ROS potentiation is a common defense strategy against diverse pathogens.

Supporting its translational potential, our analysis of natural variation revealed that *OsCRRSP1* exhibits multiple haplotypes across rice populations, with clear subpopulation differentiation and distinct geographic distributions (Supplemental Figure 3). Notably, a *japonica*-enriched haplotype is predominantly found in higher-latitude regions, suggesting an association with environmental adaptation. Although direct associations with blast resistance have not been established, these patterns indicate that *OsCRRSP1* harbors persistent genetic variation that can be exploited for resistance breeding.

From a practical perspective, OsCRRSP1 represents a promising candidate for engineering durable resistance. Its lack of yield penalties, broad effectiveness against multiple pathogens, and protein-based mode of action may limit opportunities for pathogen adaptation. In addition, stacking *OsCRRSP1* with other resistance genes could provide robust, multilayered protection while reducing selection pressure on pathogen populations.

Although our results demonstrate that OsCRRSP1 inhibits CAT activity through direct interaction, the precise molecular mechanism warrants further investigation. Notably, our interaction mapping indicates that OsCRRSP1 associates with multiple regions of CAT proteins, including the Cc domain, as well as the N- and C-terminal regions. This multi-site interaction pattern suggests that OsCRRSP1 does not function as a simple competitive inhibitor but may instead interfere with CAT activity through a more complex mechanism. One possibility is that binding of OsCRRSP1 to the Cc domain directly hinders substrate access to the active site. Alternatively, simultaneous interactions with multiple regions may impose conformational constraints on CATs, reducing their enzymatic efficiency. Such a mechanism

would be consistent with the substantial reduction in CAT activity observed *in vivo*. Additionally, given the cysteine-rich nature of DUF26 domains, OsCRRSP1 may modulate CAT function through redox-sensitive interactions, potentially affecting the structural integrity or oligomerization state of the enzyme. Although structural prediction did not identify high-confidence interaction interfaces, future structural and biochemical analyses (e.g., mutational dissection of interaction interfaces or *in vitro* kinetic assays) will be required to distinguish among these possible inhibitory mechanisms.

Several important questions remain regarding the specificity and functional properties of OsCRRSP1. For example, the molecular determinants underlying its selective interaction with OsCatB require further structural characterization. In addition, future studies should determine whether conserved cysteine residues within DUF26 domains mediate CAT interactions and whether OsCRRSP1 functions as a redox-responsive regulator.

In summary, we identify OsCRRSP1 as a central regulator that coordinates rice immune responses through the simultaneous inhibition of pathogen and host CATs (Figure 7J). This dual-targeting strategy represents a sophisticated evolutionary solution for optimizing defense by effectively amplifying ROS signals across cellular compartments while minimizing fitness costs. Our findings not only expand the functional repertoire of DUF26 proteins but also provide a promising genetic resource for breeding durable disease resistance.

METHODS

Plant materials and *M. oryzae* growth conditions

Rice (*Oryza sativa* L.) varieties used in this study included wild-type ZH11 (*japonica*), *Oscrrsp1* mutants, *OsCRRSP1*-OE lines, and *MoCatB*-OE lines (all in the ZH11 background). Plants were cultivated in a greenhouse under a 14 h light/10 h dark photoperiod at 26°C ± 2°C and 70% relative humidity. *M. oryzae* strains included wild-type Guy11, the Δ*Mocatb* mutant, the Δ*Mocatb* complemented strain, and the GFP-tagged strain MoCatB-GFP. All fungal strains were cultured on CM (yeast extract [6 g/l], sucrose [10 g/l], casein hydrolysate [10 g/l], and agar [20 g/l] or rice bran medium (rice bran [40 g/l], and agar [20 g/l]) at 28°C. Conidiation was induced under continuous light for 2–3 days, and conidia were harvested in sterile water for inoculation assays. *N. benthamiana* plants were grown at 22°C under a 9 h light/15 h dark photoperiod with a light intensity of 7000–8000 lux.

Plasmid construction and rice transformation

Oscrrsp1 knockout mutants were generated in the ZH11 background via CRISPR-Cas9-mediated gene editing (Biogle Biotechnology, Hangzhou, China). The single guide RNA sequence is shown in Figure 1A. Mutants were identified by PCR amplification of genomic DNA using primers flanking the CRISPR/Cas9 target site (Supplemental Table 4), followed by sequencing. Two independent homozygous lines, *Oscrrsp1-1* and *Oscrrsp1-2*, were obtained and validated (Figure 1A).

For overexpression constructs, the full-length coding sequences of *OsCRRSP1* and *MoCatB* were amplified and fused in-frame to *GFP* to generate OsCRRSP1-GFP and MoCatB-GFP, respectively. The fusion constructs were cloned into the pCAMBIA1300 vector under the control of the *CaMV 35S* promoter and introduced into ZH11 rice plants via *Agrobacterium tumefaciens* strain EHA105-mediated transformation. Positive transformants were selected on medium containing geneticin (G418). Transgene expression was confirmed at both the transcript and protein levels by RT−qPCR and immunoblot analysis, respectively. The primers used for RT–qPCR are listed in Supplemental Table 4. Independent T2 lines with confirmed transgene expression were used for subsequent experiments.

Generation of *MoCatB* knockout and complemented strains

MoCatB deletion mutants were generated using a one-step gene replacement strategy with the pBS-*NEO* vector (Tang et al., 2015). A 1.014 kb fragment upstream of the *MoCatB coding region* and a 1.021 kb fragment downstream of the *MoCatB* coding region were amplified using primers listed in Supplemental Table 4 and then inserted into pBS-*NEO* to generate the *MoCatB*-pBS-UP-HYG-DW construct. The construct was transformed into Guy11 using a previously described method (Li et al., 2012). Transformants were selected with hygromycin and verified by PCR and sequencing (Supplemental Table 4).

For complementation, the full-length *MoCatB* open reading frame, excluding the stop codon, was amplified using the primer pair MoCatB-pKNTG-F/R (Supplemental Table 4) and cloned into the pKNTG vector to generate a C-terminal GFP fusion driven by the native *MoCatB* promoter (Zheng et al., 2016). The resulting plasmid was transformed into protoplasts of the Δ*Mocatb*#10 mutant. Complemented strains were confirmed via PCR, sequencing, and GFP fluorescence. Strains expressing MoCatB-GFP and MoCatB-GFP/Pwl2-mCherry in the Guy11 background were generated using a similar strategy.

Antifungal activity assay

The antifungal activity of recombinant OsCRRSP1 was assessed as previously described (Han et al., 2019) with minor modifications. *M. oryzae* Guy11 was cultured on CM plates at 28°C. A 5 mm mycelial plug was placed at the center of a fresh CM plate, and 4 sterile filter paper discs (6 mm diameter) were positioned equidistantly around it. The following solutions (20 μl each) were applied to the discs: purified GST-OsCRRSP1 (1 μg/μl), GST protein (1 μg/μl, negative control), sterile distilled water (blank control), and sodium hypochlorite (NaClO, fungicide, positive control). Plates were incubated at 28°C for 6 days without inversion. Inhibition zones were documented photographically.

Rice blast inoculation and disease evaluation

Spray and punch inoculations were performed as previously described (Yang et al., 2019). For spray inoculation, 3-week-old rice seedlings were sprayed with an *M. oryzae* conidial suspension (2×10^5 conidia ml^{-1} in 0.02% Tween 20). For punch inoculation, 10 μl of conidial suspension (1×10^5 conidia ml^{-1}) was applied to wounded leaves of 4-week-old plants. Inoculated plants were placed in a dew chamber at 26°C in darkness for 24 h, then transferred to a growth chamber (26°C, 12 h/12 h light/dark photoperiod, 90% relative humidity) for 4–7 days. Disease symptoms were imaged; fungal biomass was quantified by RT−qPCR using primers specific for the *Pot2* transposon and normalized to the rice ubiquitin gene *OsUBQ* (LOC_Os03g13170). PCR primers are listed in Supplemental Table 4.

Sheath inoculation assays using *M. oryzae* strains Guy11, Δ*Mocatb*, and Com/#10 were conducted as previously described (Khang et al., 2010) with slight modifications. Rice leaf sheaths were injected with conidial suspensions (1×10^5 conidia ml^{-1}) and incubated at 28°C in darkness. IH growth was observed at 24 and 48 hpi using a confocal microscope (LSM 880, Carl Zeiss).

All infection assays were independently repeated at least three times with similar results. Representative data from one independent experiment are shown.

Bacterial blight (*X. oryzae pv. oryzae*) inoculation

X. oryzae pv. oryzae strain PXO99 was kindly provided by Prof. Mo Wang (Yunnan Agricultural University, China). *X. oryzae pv. oryzae* inoculations

were performed in the experimental field as previously described (Tian et al., 2020). Bacterial cultures were grown in liquid medium (sucrose [20 g/l], casein tryptone [5 g/l], $FeSO_4 \cdot 7H_2O$ [0.5 g/l], $Ca(NO_3)_2 \cdot 4H_2O$ [0.5 g/l], and Na_2HPO_4 [2 g/l]) with shaking at 220 rpm for 48 h at 28°C. Cells were then resuspended in H_2O_2 to an optical density at 600 nm of 0.5 for inoculation. Leaves of rice plants at the tillering stage were inoculated using the leaf-clipping method with scissors dipped in the bacterial suspension. For each rice genotype, 5–10 plants and 3–5 fully expanded leaves per plant were inoculated. Lesion lengths on infected leaves were measured 2 weeks after inoculation. All infection assays were independently repeated at least three times with similar results. Representative data from one independent experiment are shown.

Haplotype analysis of *OsCRRSP1*

SNP data for *OsCRRSP1* and its surrounding regions (50 kb flanking sequence on each side) were obtained from the Rice SNP-seek Database (https://snpseekv3.duckdns.org). Variants within the target region were extracted using PLINK (v1.9) and filtered using minor-allele frequency $\geq$ 0.05 and missing rate $\leq$ 0.1. After filtering, four high-quality SNPs (one in the coding sequence and three in the 3′ UTR) were retained. Haplotypes were constructed by concatenating alleles across SNP sites for each accession, with genotype codes converted to nucleotide bases based on PLINK.bim annotations. Haplotype frequencies were calculated for the entire population and across subpopulations; haplotypes with frequencies $\geq$ 5% were retained for further analysis. Subpopulation classifications and geographic coordinates of accessions were integrated to assess haplotype distributions. Spatial patterns were visualized on a global map, where circle size represented haplotype frequency and colors indicated haplotype groups. All analyses were performed using custom Python scripts.

RNA extraction and RT–qPCR assays

Total RNA was extracted from leaf tissues using TRIGene reagent (GenStar, Beijing, China; p118-05). First-strand cDNA was synthesized from 1 μg of RNA using EasyScript One-Step gDNA Removal and cDNA Synthesis SuperMix (TransGen, Beijing, China; catalog number AE311). RT−qPCR was performed using Perfect Start Green qPCR SuperMix (TransGen, AQ601) on a CFX Connect real-time PCR system (Bio-Rad, Hercules, CA, USA). Relative expression levels were calculated using the $2^{-\Delta\Delta CT}$ method and normalized to the *OsUBQ* gene. Primer sequences are listed in Supplemental Table 4.

ROS burst assay

ROS production was measured as previously described (Gao et al., 2021) with modifications. Leaf discs from 7-day-old seedlings were excised and suspended in 200 μl water in a 96-well plate overnight. The water was then replaced with 200 μl of reaction solution containing 20 μM luminol (L-012, Fujifilm, catalog number 120-04891), 10 μg/ml horseradish peroxidase, and 800 nM chitin. Luminescence was immediately recorded at 1-min intervals over a 40-min period using a GM2000 luminometer (Promega).

Callose deposition assay

For visualization and quantification of chitin-induced callose deposition, leaves from 7-day-old seedlings were detached and incubated with 800 nM chitin for 16 h. The assay was performed as previously described (Yang et al., 2019). Callose deposits were stained and visualized under UV light (excitation, 405 nm; emission, 498 nm) using a Carl Zeiss LSM800 microscope. Deposits were quantified across all fields of view using ImageJ software (version 1.43U).

DAB staining

H_2O_2 accumulation was detected via DAB staining as previously described (Gao et al., 2021). Rice leaves inoculated with *M. oryzae* were immersed in DAB solution (1 mg/ml DAB, 10 mM 2-(N-morpholino)ethanesulfonic acid [pH 3.8] containing 0.2% Tween 20) at 48 hpi. After vacuum infiltration for 30 min, samples were incubated overnight in darkness with gentle shaking. Leaves were decolorized in ethanol at 95°C and imaged using a charge-coupled device-equipped microscope. Brown staining intensity was quantified using ImageJ software.

Phylogenetic analysis and multiple sequence alignment

For phylogenetic analysis of CRRSP proteins from rice, maize, *Arabidopsis*, and cotton, protein sequences were collected from the Rice Genome Database (http://rice.uga.edu/), CottonGen (https://www.cottongen.org/), NCBI (https://www.ncbi.nlm.nih.gov/), and Phytozome (https://phytozome-next.jgi.doe.gov/). Multiple sequence alignments were performed using ClustalW. The neighbor-joining method implemented in MEGA7.0 was used to generate the phylogenetic tree. Bootstrap values shown at the nodes were based on 1,000 replications. Amino acid sequence alignment was performed with DNAMAN 8 software.

Yeast assay for validating signal peptide-mediated secretion

SP secretion activity was assessed as previously described (Xu et al., 2019). The SP region was predicted using the online tool SignalP 4.1. The SP-encoding sequence was cloned into the pSUC2t7M13ori vector (Jacobs et al., 1997) and transformed into yeast strain YTK12. Transformants were selected on CMD-W (tryptophan-deficient) plates at 30°C for 3 days in darkness. Secretion activity was assessed based on growth on YPRAA plates lacking glucose. Invertase activity was also determined through reduction of TTC to insoluble red 1,3,5-triphenylformazan.

Protein expression in rice protoplasts

For protein expression in rice protoplasts, plasmids were constructed and purified using a Plasmid Maxi Kit. Rice seedlings were grown in darkness for 20 days, and protoplasts were subsequently isolated from the seedlings. Plasmids were introduced into rice protoplasts through polyethylene glycol (PEG4000)/calcium-mediated transformation. After 14 h of incubation, proteins were either extracted using buffer (50 mM Tris [pH 7.5], 150 mM NaCl, 10% [v/v] glycerol, 2 mM EDTA, 0.1% Triton X-100, 1× protease inhibitor [Roche], and 5 mM DTT) or directly observed using a confocal microscope (Carl Zeiss LSM880).

Extraction of rice apoplastic proteins

Apoplastic proteins were extracted using a modified vacuum infiltration–centrifugation method (Kim et al., 2013). Briefly, 2 g of fresh leaves from *OsCRRSP1*-OE rice were placed into a 10 ml syringe barrel (without needle, plunger, or push rod), rinsed twice with pre-chilled double-distilled water, and sealed with aluminum foil. The syringe was placed in a 50 ml centrifuge tube filled with pre-chilled apoplastic protein extraction buffer (0.1 M Tris [pH 7.6], 0.2 M KCl, and 1 mM PMSF). The tube was placed in an ice-filled 500 ml beaker and subjected to vacuum infiltration at 35 kPa for 15 min. Samples were centrifuged at 4°C and 1,000 *g* for 10 min, and the process was repeated once. Extracts from both centrifugation steps were pooled to obtain apoplastic protein extracts. For total protein extraction, 0.2 g of leaf tissue from the syringe was processed with protein extraction buffer (50 mM Tris [pH 7.5], 150 mM NaCl, 10% [v/v] glycerol, 2 mM EDTA, 0.1% Triton X-100, 1× protease inhibitor [Roche], and 5 mM DTT). Immunoblot analysis was performed using anti-GFP and anti-actin antibodies.

Cell fractionation

Rice protoplasts were isolated from enzymatically digested etiolated seedlings of *OsCRRSP1*-OE rice. A plasmid encoding the rice plasma membrane protein OsSOBIR1-mCherry was introduced into rice protoplasts through PEG4000/calcium-mediated transformation. After 14 h of expression, cells were collected; membrane and cytoplasmic proteins were extracted using the Membrane and Cytosol Protein Extraction Kit (Beyotime, catalog number P0033). GFP antibodies (TransGen, catalog number HT801) were used to detect OsCRRSP1 protein, whereas plant actin antibodies (Engibody, catalog number AT0004) and mCherry

antibodies (Biodragon, catalog number B1010) were used to detect cytoplasmic and membrane proteins, respectively.

LC-MS/MS analysis

To identify *M. oryzae*-secreted proteins that interact with OsCRRSP1, IP followed by liquid chromatography–tandem MS (LC–MS/MS) was performed. Recombinant OsCRRSP1 protein fused to a YFP tag was purified by affinity chromatography. The purified protein was incubated with total proteins extracted from the culture supernatant of the Δ*Pokmt6* mutant strain of *M. oryzae* (Xie et al., 2023), which had been pre-induced by rice leaves. OsCRRSP1-YFP and its associated protein complexes were immunoprecipitated using GFP-NanoAb-agarose beads. A 10 μl aliquot from a total elution volume of 70 μl was mixed with 10 μl of 2× loading buffer and analyzed by SDS–PAGE to verify co-precipitated complexes. After visual confirmation by Coomassie staining, the remaining samples were subjected to LC-MS/MS analysis. Tandem mass spectra were searched against the *M. oryzae* strain 70-15 reference proteome to identify putative OsCRRSP1-interacting proteins.

CoIP and immunoblot analysis

For co-IP assays, the p35S:OsCRRSP1-HA and p35S:MoCatB-YFP constructs were co-transformed into *N. benthamiana* leaves through *Agrobacterium*-mediated infiltration. Two days post-infiltration, leaves were harvested and ground in liquid nitrogen. Total proteins were extracted using ice-cold extraction buffer (50 mM Tris [pH 7.5], 150 mM NaCl, 10% [v/v] glycerol, 2 mM EDTA, 0.1% Triton X-100, 1× protease inhibitor [Roche], and 5 mM DTT), in accordance with an established protocol (Zhao et al., 2021). Homogenates were centrifuged at 20,000 *g* for 15 min at 4°C, and supernatants were collected. An aliquot was reserved as the input sample. For IP, each supernatant was incubated with 15 μl of GFP-NanoAb-agarose beads (LabLead, GNA-50-1000) in 1.5 ml tubes for 2 h at 4°C with gentle rotation. Subsequently, beads were washed 5 times with 1× PBS buffer. Bound proteins were eluted in 2× Laemmli buffer, separated by SDS–PAGE, and transferred to a nitrocellulose (NC) membrane for immunoblot analysis. Membranes were probed with anti-GFP antibody (TransGen, catalog number HT801) and anti-HA antibody (Roche, catalog number 11867423001). Protein–antibody complexes were detected using horseradish peroxidase-conjugated secondary antibodies and visualized by enhanced chemiluminescence (Abbkine Scientific, Wuhan, China; goat anti-mouse immunoglobulin G, catalog number A21010; goat anti-rabbit immunoglobulin G, catalog number A21020).

Protein pull-down assay

For GST pull-down assays, the coding sequence of *OsCRRSP1* was cloned into the pET28a-C-YFP vector to generate a C-terminal YFP fusion, and *MoCatB* was cloned into the pGEX-6p-1 vector to generate an N-terminal GST fusion. Recombinant proteins were expressed in *E. coli* and purified according to the manufacturer's protocols. Pull-down assays were performed as previously described (Ling et al., 2022). Briefly, purified GST or GST-MoCatB fusion proteins were immobilized on glutathione-Sepharose beads and incubated with purified OsCRRSP1-YFP in binding buffer. After extensive washing, bound proteins were eluted and analyzed by immunoblotting using an anti-GFP antibody. Input and pull-down samples were also visualized by Coomassie Brilliant Blue staining.

Y2H assay

A Y2H system (Clontech, USA, catalog number 630489) was used to test interactions between OsCRRSP1 and MoCatB. The coding sequence of *OsCRRSP1* was cloned into the pGADT7 vector between the *NdeI* and *EcoRI* restriction sites. The coding sequences of *MoCatB*, *MoCatB-Cc*, and *MoCatB-3′* were cloned into the pGBKT7 vector between the *NdeI* and *EcoRI* restriction sites. Constructs were co-transformed into *Saccharomyces cerevisiae* strain AH109, in accordance with the manufacturer's instructions. Transformants were selected on SD/−Trp−Leu medium (Coolaber, PM2220). Protein interactions were assessed by plating yeast colonies on high-stringency SD/−Trp−Leu−His−Ade medium (Coolaber, PM2110). The pGBKT7-53/pGADT7-T and pGBKT7/pGADT7 pairs served as positive and negative controls, respectively.

Split-LUC complementation assay

Split-LUC complementation assays were performed as previously described (Leng et al., 2023). The coding sequences of *OsCRRSP1* and *OsCRRSP1ΔSP* (lacking signal peptide) were fused to the N-terminal fragment of luciferase (nLUC), whereas that of *MoCatB* was fused to the C-terminal fragment (cLUC). These constructs were transformed into *A. tumefaciens* strain GV3101. Bacterial cultures were adjusted to an optical density at 600 nm of 1.2, mixed at a 1:1 ratio, and incubated in darkness for 2–3 h before infiltration into *N. benthamiana* leaves. After 48 h, infiltrated leaves were injected with 1 mM luciferin and kept in darkness for 5 min to allow substrate diffusion. Luciferase activity was imaged using a cooled charge-coupled device camera system.

Catalase (CAT) activity assay

The CAT activities of MoCatB-YFP and OsCatB-YFP proteins expressed in rice protoplasts were measured using a CAT assay kit (Beyotime Biotechnology, Shanghai, China; catalog number S0051), in accordance with the manufacturer's instructions. Protoplasts expressing the target proteins were collected by centrifugation at 300 *g* for 3 min at 25°C. After removal of the supernatant, total proteins were extracted using ice-cold extraction buffer (50 mM Tris [pH 7.5], 150 mM NaCl, 10% [v/v] glycerol, 2 mM EDTA, 0.1% Triton X-100, 1× protease inhibitor [Roche], and 5 mM DTT). Lysates were incubated on ice for 10 min, then centrifuged at 20,000 *g* for 10 min at 4°C. The resulting supernatants were incubated with anti-GFP agarose beads for 2 h at 4°C to enrich target proteins. Beads were washed 5 times with wash buffer (50 mM Tris [pH 7.5], 150 mM NaCl, 10% [v/v] glycerol, 2 mM EDTA, and 0.1% Triton). CAT activities of the immunoprecipitated proteins were subsequently measured.

For measurement of CAT activity in plant tissues, total proteins were extracted from leaves of ZH11, *Oscrrsp1*, *MoCatB*-OE, and *OsCRRSP1*-OE plants using the CheKine Micro Catalase Activity Assay Kit (Abbkine Scientific, Wuhan, China; catalog number KTB1040) according to the manufacturer's protocol (Lin et al., 2024). Briefly, 0.1 g of leaf tissue was ground into a fine powder in liquid nitrogen and homogenized in 1 ml of ice-cold 1× lysis buffer. The homogenate was centrifuged at 10,000 *g* for 15 min at 4°C, and the supernatant was collected for immediate CAT activity analysis.

Quantification and statistical analysis

Statistical analyses and graph generation for fungal biomass, lesion length, CAT activity, ROS bursts, lesion area, and other quantified parameters were conducted using GraphPad Prism 9 (GraphPad Software, San Diego, CA, USA). All data are presented as mean ± SE. Exact values are indicated in the figures or figure legends. Detailed descriptions of quantification procedures and statistical analyses are provided in the figures, figure legends, or methods section. Statistical significance was assessed by Student's *t*-test, with $p < 0.05$ considered statistically significant and $p < 0.01$ considered highly significant. ImageJ was used for image counting, area measurement, and quantification of western blot band intensities.

Accession numbers

Sequence data from this article are available in GenBank under the following accession numbers: OsCRRSP1, Os04g25060; MoCatB, MGG_06442; PoKmt6, MGG_00152; OsCatB, Os06g51150; OsCatA, Os02g02400; OsCatC, Os03g03910; OsUBQ, Os03g13170; OsRMC, Os04g56430; OsSOBIR1, Os06g18000; OsPR3, Os04g41620; OsPBZ1, Os12g36880; OsPAL1, Os02g41680; JIOsPR10, Os03g18850; GhCRR1, Gbscaffold10424.22; GhCRR2, Gbscaffold10424.17; GhCRR3, Gbscaffold10424.16; GhCRR4, Gbscaffold15923.8.

FUNDING

This work was supported by grants from the Taishan Scholar Project of Shandong Province (tsqn202306149) to L.X., the Natural Science Foundation of Fujian Province Key Program (2023J02011) to H.C., the National Natural Science Foundation of China (32470263), and the "First Class Discipline" Construction Project of Shandong Agricultural University (SKL81105 and SKL81124) to H.C. and L.X.

ACKNOWLEDGMENTS

We thank Prof. Dingzhong Tang (State Key Laboratory of Ecological Control of Fujian-Taiwan Crop Pests, Fujian Agriculture and Forestry University) for kindly providing the pCAMBIA1300-nLUC and pCAMBIA1300-cLUC vectors. We also thank Prof. Zonghua Wang (College of Life Science, Fujian Agriculture and Forestry University) for kindly providing the *M. oryzae* strain Guy11.The authors declare no competing interests.

AUTHOR CONTRIBUTIONS

Conceptualization, Z. Zhong, L.X., and H.C.; methodology, Z. Zhong, Z. Zheng, and H.C.; investigation, Z. Zhong, Z. Zheng, M.Z., G.W., L.Z., and H.Z.; visualization, Z. Zhong and G.W.; data curation, Z. Zhong and H.C.; supervision, Z. Zhong, L.X., and H.C.; writing – original draft, Z. Zhong; writing – review and editing, Z. Zhong, L.X., and H.C.; funding acquisition, L.X. and H.C. All the authors read and approved the final version of the manuscript.

SUPPLEMENTAL INFORMATION

Supplemental information is available at *Plant Communications Online*.

Received: November 3, 2025
Revised: April 5, 2026
Accepted: April 27, 2026
Published: April 30, 2026

REFERENCES

Apostol, I., Heinstein, P.F., and Low, P.S. (1989). Rapid Stimulation of an Oxidative Burst during Elicitation of Cultured Plant Cells : Role in Defense and Signal Transduction. Plant Physiol. **90**:109–116. https://doi.org/10.1104/pp.90.1.109.

Caillaud, M.C., Wirthmueller, L., Sklenar, J., et al. (2014). The plasmodesmal protein PDLP1 localises to haustoria-associated membranes during downy mildew infection and regulates callose deposition. PLoS Pathog. **10**:e1004496. https://doi.org/10.1371/journal.ppat.1004496.

Chen, Z. (2001). A Superfamily of Proteins with Novel Cysteine-Rich Repeats. Plant Physiol. **126**:473–476. https://doi.org/10.1104/pp.126.2.473.

Chern, M., Xu, Q., Bart, R.S., Bai, W., Ruan, D., Sze-To, W.H., Canlas, P.E., Jain, R., Chen, X., and Ronald, P.C. (2016). A Genetic Screen Identifies a Requirement for Cysteine-Rich-Receptor-Like Kinases in Rice NH1 (OsNPR1)-Mediated Immunity. PLoS Genet. **12**:e1006049. https://doi.org/10.1371/journal.pgen.1006049.

Dean, R., Van Kan, J.A.L., Pretorius, Z.A., Hammond-Kosack, K.E., Di Pietro, A., Spanu, P.D., Rudd, J.J., Dickman, M., Kahmann, R., Ellis, J., and Foster, G.D. (2012). The Top 10 fungal pathogens in molecular plant pathology. Mol. Plant Pathol. **13**:414–430. https://doi.org/10.1111/j.1364-3703.2011.00783.x.

Del Corpo, D., Coculo, D., Greco, M., De Lorenzo, G., and Lionetti, V. (2024). Pull the fuzes: Processing protein precursors to generate apoplastic danger signals for triggering plant immunity. Plant Commun. **5**:100931. https://doi.org/10.1016/j.xplc.2024.100931.

Dietz, K.-J. (1997). Functions and Responses of the Leaf Apoplast Under Stress. In Progress in Botany: Structural Botany Physiology Genetics Taxonomy Geobotany/Fortschritte der Botanik Struktur Physiologie Genetik Systematik Geobotanik, H.D. Behnke, U. Lüttge, K. Esser, J.W. Kadereit, and M. Runge, eds. (Springer Berlin Heidelberg), pp. 221–254. https://doi.org/10.1007/978-3-642-60458-4_9.

Doehlemann, G., and Hemetsberger, C. (2013). Apoplastic immunity and its suppression by filamentous plant pathogens. New Phytol. **198**:1001–1016. https://doi.org/10.1111/nph.12277.

Dora, S., Terrett, O.M., and Sánchez-Rodríguez, C. (2022). Plant–microbe interactions in the apoplast: Communication at the plant cell wall. Plant Cell **34**:1532–1550. https://doi.org/10.1093/plcell/koac040.

Gao, M., He, Y., Yin, X., Zhong, X., Yan, B., Wu, Y., Chen, J., Li, X., Zhai, K., Huang, Y., et al. (2021). Ca^{2+} sensor-mediated ROS scavenging suppresses rice immunity and is exploited by a fungal effector. Cell **184**:5391–5404.e17. https://doi.org/10.1016/j.cell.2021.09.009.

Giraldo, M.C., Dagdas, Y.F., Gupta, Y.K., Mentlak, T.A., Yi, M., Martinez-Rocha, A.L., Saitoh, H., Terauchi, R., Talbot, N.J., and Valent, B. (2013). Two distinct secretion systems facilitate tissue invasion by the rice blast fungus *Magnaporthe oryzae*. Nat. Commun. **4**:1996. https://doi.org/10.1038/ncomms2996.

Han, L.B., Li, Y.B., Wang, F.X., Wang, W.Y., Liu, J., Wu, J.H., Zhong, N.Q., Wu, S.J., Jiao, G.L., Wang, H.Y., and Xia, G.X. (2019). The Cotton Apoplastic Protein CRR1 Stabilizes Chitinase 28 to Facilitate Defense against the Fungal Pathogen *Verticillium dahliae*. Plant Cell **31**:520–536. https://doi.org/10.1105/tpc.18.00390.

Idänheimo, N., Gauthier, A., Salojärvi, J., Siligato, R., Brosché, M., Kollist, H., Mähönen, A.P., Kangasjärvi, J., and Wrzaczek, M. (2014). The *Arabidopsis thaliana* cysteine-rich receptor-like kinases CRK6 and CRK7 protect against apoplastic oxidative stress. Biochem. Biophys. Res. Commun. **445**:457–462. https://doi.org/10.1016/j.bbrc.2014.02.013.

Jacobs, K.A., Collins-Racie, L.A., Colbert, M., Duckett, M., Golden-Fleet, M., Kelleher, K., Kriz, R., LaVallie, E.R., Merberg, D., Spaulding, V., et al. (1997). A genetic selection for isolating cDNAs encoding secreted proteins. Gene **198**:289–296. https://doi.org/10.1016/s0378-1119(97)00330-2.

Jiang, J., Li, J., Xu, Y., et al. (2007). RNAi knockdown of *Oryza sativa root meander curling* gene led to altered root development and coiling which were mediated by jasmonic acid signalling in rice. Plant Cell Environ. **30**:690–699. https://doi.org/10.1111/j.1365-3040.2007.01663.x.

Jones, J.D.G., and Dangl, J.L. (2006). The plant immune system. Nature **444**:323–329. https://doi.org/10.1038/nature05286.

Khang, C.H., Berruyer, R., Giraldo, M.C., et al. (2010). Translocation of *Magnaporthe oryzae* effectors into rice cells and their subsequent cell-to-cell movement. Plant Cell **22**:1388–1403. https://doi.org/10.1105/tpc.109.069666.

Kim, S.G., Wang, Y., Lee, K.H., et al. (2013). In-depth insight into in vivo apoplastic secretome of rice-*Magnaporthe oryzae* interaction. J. Proteomics **78**:58–71. https://doi.org/10.1016/j.jprot.2012.10.029.

Kimura, S., Hunter, K., Vaahtera, L., Tran, H.C., Citterico, M., Vaattovaara, A., Rokka, A., Stolze, S.C., Harzen, A., Meißner, L., et al. (2020). CRK2 and C-terminal Phosphorylation of NADPH Oxidase RBOHD Regulate Reactive Oxygen Species Production in Arabidopsis. Plant Cell **32**:1063–1080. https://doi.org/10.1105/tpc.19.00525.

Lee, J.Y., Wang, X., Cui, W., et al. (2011). A plasmodesmata-localized protein mediates crosstalk between cell-to-cell communication and innateImmunity in Arabidopsis. Plant Cell **23**:3353–3373. https://doi.org/10.1105/tpc.111.087742.

Leng, J., Wei, X., Jin, X., Wang, L., Fan, K., Zou, K., Zheng, Z., Saridis, G., Zhao, N., Zhou, D., et al. (2023). ARBUSCULAR MYCORRHIZA-INDUCED KINASES AMK8 and AMK24 associate with the receptor-like kinase KINASE3 to regulate arbuscular mycorrhizal symbiosis in

Lotus japonicus. Plant Cell **35**:2006–2026. https://doi.org/10.1093/plcell/koad050.

Li, G.-B., He, J.-X., Wu, J.-L., Wang, H., Zhang, X., Liu, J., Hu, X.-H., Zhu, Y., Shen, S., Bai, Y.-F., et al. (2022). Overproduction of OsRACK1A, an effector-targeted scaffold protein promoting OsRBOHB-mediated ROS production, confers rice floral resistance to false smut disease without yield penalty. Mol. Plant **15**:1790–1806. https://doi.org/10.1016/j.molp.2022.10.009.

Li, H.J., Lu, J.P., Liu, X.H., Zhang, L.L., and Lin, F.C. (2012). Vectors building and usage for gene knockout, protein expression and fluorescent fusion protein in the rice blast fungus. J. Agric. Biotechnol. **20**:94–104.

Li, W., Deng, Y., Ning, Y., He, Z., and Wang, G.L. (2020). Exploiting Broad-Spectrum Disease Resistance in Crops: From Molecular Dissection to Breeding. Annu. Rev. Plant Biol. **71**:575–603. https://doi.org/10.1146/annurev-arplant-010720-022215.

Lin, Y., Yang, H., Liu, H., Lu, X., Cao, H., Li, B., Chang, Y., Guo, Z., Ding, D., Hu, Y., et al. (2024). A P-type pentatricopeptide repeat protein ZmRF5 promotes 5' region partial cleavages of atp6c transcripts to restore the fertility of CMS-C maize by recruiting a splicing factor. Plant Biotechnol. J. **22**:1269–1281. https://doi.org/10.1111/pbi.14263.

Ling, H., Fu, X., Huang, N., Zhong, Z., Su, W., Lin, W., Cui, H., and Que, Y. (2022). A sugarcane smut fungus effector simulates the host endogenous elicitor peptide to suppress plant immunity. New Phytol. **233**:919–933. https://doi.org/10.1111/nph.17835.

Ma, L.S., Wang, L., Trippel, C., Mendoza-Mendoza, A., Ullmann, S., Moretti, M., Carsten, A., Kahnt, J., Reissmann, S., Zechmann, B., et al. (2018). The *Ustilago maydis* repetitive effector Rsp3 blocks the antifungal activity of mannose-binding maize proteins. Nat. Commun. **9**:1711. https://doi.org/10.1038/s41467-018-04149-0.

Ma, Z., Zhu, L., Song, T., Wang, Y., Zhang, Q., Xia, Y., Qiu, M., Lin, Y., Li, H., Kong, L., et al. (2017). A paralogous decoy protects *Phytophthora sojae* apoplastic effector PsXEG1 from a host inhibitor. Science **355**:710–714. https://doi.org/10.1126/science.aai7919.

Mhamdi, A., Queval, G., Chaouch, S., Vanderauwera, S., Van Breusegem, F., and Noctor, G. (2010). Catalase function in plants: a focus on Arabidopsis mutants as stress-mimic models. J. Exp. Bot. **61**:4197–4220. https://doi.org/10.1093/jxb/erq282.

Mittler, R., Zandalinas, S.I., Fichman, Y., and Van Breusegem, F. (2022). Reactive oxygen species signalling in plant stress responses. Nat. Rev. Mol. Cell Biol. **23**:663–679. https://doi.org/10.1038/s41580-022-00499-2.

Ngou, B.P.M., Ding, P., and Jones, J.D.G. (2022). Thirty years of resistance: Zig-zag through the plant immune system. Plant Cell **34**:1447–1478. https://doi.org/10.1093/plcell/koac041.

Ni, L., Wang, Q., Chen, C., Wang, S., Shen, T., Jiang, J., Cui, Z., Li, K., Yang, Q., and Jiang, M. (2022). OsDMI3-mediated OsUXS3 phosphorylation improves oxidative stress tolerance by modulating OsCATB protein abundance in rice. J. Integr. Plant Biol. **64**:1087–1101. https://doi.org/10.1111/jipb.13255.

Qi, J., Wang, J., Gong, Z., and Zhou, J.M. (2017). Apoplastic ROS signaling in plant immunity. Curr. Opin. Plant Biol. **38**:92–100. https://doi.org/10.1016/j.pbi.2017.04.022.

Sawano, Y., Miyakawa, T., Yamazaki, H., Tanokura, M., and Hatano, K.i. (2007). Purification, characterization, and molecular gene cloning of an antifungal protein from *Ginkgo biloba* seeds. Biol. Chem. **388**:273–280. https://doi.org/10.1515/BC.2007.030.

Skamnioti, P., Henderson, C., Zhang, Z., Robinson, Z., and Gurr, S.J. (2007). A novel role for catalase B in the maintenance of fungal cell-wall integrity during host invasion in the rice blast fungus *Magnaporthe grisea*. Mol. Plant Microbe Interact. **20**:568–580. https://doi.org/10.1094/MPMI-20-5-0568.

Takeda, T., Takahashi, M., Shimizu, M., Sugihara, Y., Yamashita, T., Saitoh, H., Fujisaki, K., Ishikawa, K., Utsushi, H., Kanzaki, E., et al. (2022). Rice apoplastic CBM1-interacting protein counters blast pathogen invasion by binding conserved carbohydrate binding module 1 motif of fungal proteins. PLoS Pathog. **18**:e1010792. https://doi.org/10.1371/journal.ppat.1010792.

Tang, W., Ru, Y., Hong, L., Zhu, Q., Zuo, R., Guo, X., Wang, J., Zhang, H., Zheng, X., Wang, P., and Zhang, Z. (2015). System-wide characterization of bZIP transcription factor proteins involved in infection-related morphogenesis of *Magnaporthe oryzae*. Environ. Microbiol. **17**:1377–1396. https://doi.org/10.1111/1462-2920.12618.

Tian, D., Yang, F., Niu, Y., Lin, Y., Chen, Z., Li, G., Luo, Q., Wang, F., and Wang, M. (2020). Loss function of SL (sekiguchi lesion) in the rice cultivar Minghui 86 leads to enhanced resistance to (hemi)biotrophic pathogens. BMC Plant Biol. **20**:507. https://doi.org/10.1186/s12870-020-02724-6.

Vaattovaara, A., Brandt, B., Rajaraman, S., et al. (2019). Mechanistic insights into the evolution of DUF26-containing proteins in land plants. Commun. Biol. **2**:56. https://doi.org/10.1038/s42003-019-0306-9.

Wang, Y., Pruitt, R.N., Nürnberger, T., and Wang, Y. (2022). Evasion of plant immunity by microbial pathogens. Nat. Rev. Microbiol. **20**:449–464. https://doi.org/10.1038/s41579-022-00710-3.

Wang, Y., Teng, Z., Li, H., Wang, W., Xu, F., Sun, K., Chu, J., Qian, Y., Loake, G.J., Chu, C., and Tang, J. (2023). An activated form of NB-ARC protein RLS1 functions with cysteine-rich receptor-like protein RMC to trigger cell death in rice. Plant Commun. **4**:100459. https://doi.org/10.1016/j.xplc.2022.100459.

Wei, X., Liu, N., Yang, W., Hou, W., Hou, Y., Kang, Z., and Liu, J. (2025). Stripe rust effector Pst9653 interferes with subcellular distributions of catalase TaCAT3 to facilitate susceptibility-involved ROS scavenging in wheat. New Phytol. **247**:2885–2902. https://doi.org/10.1111/nph.70341.

Xie, J.-h., Tang, W., Lu, G.-d., Hong, Y.-h., Zhong, Z.-h., Wang, Z.-h., and Zheng, H.-k. (2023). Histone H3K27me3 methylation regulates the expression of secreted proteins distributed at fast-evolving regions through transcriptional repression of transposable elements. J. Integr. Agric. **22**:3059–3068. https://doi.org/10.1016/j.jia.2023.01.011.

Xu, Q., Tang, C., Wang, X., Sun, S., Zhao, J., Kang, Z., and Wang, X. (2019). An effector protein of the wheat stripe rust fungus targets chloroplasts and suppresses chloroplast function. Nat. Commun. **10**:5571. https://doi.org/10.1038/s41467-019-13487-6.

Yang, C., Yu, Y., Huang, J., Meng, F., Pang, J., Zhao, Q., Islam, M.A., Xu, N., Tian, Y., and Liu, J. (2019). Binding of the *Magnaporthe oryzae* Chitinase MoChia1 by a Rice Tetratricopeptide Repeat Protein Allows Free Chitin to Trigger Immune Responses. Plant Cell **31**:172–188. https://doi.org/10.1105/tpc.18.00382.

Yang, D., Li, S., Xiao, Y., Lu, L., Zheng, Z., Tang, D., and Cui, H. (2021). Transcriptome analysis of rice response to blast fungus identified core genes involved in immunity. Plant Cell Environ. **44**:3103–3121. https://doi.org/10.1111/pce.14098.

You, X., Zhang, F., Liu, Z., Wang, M., Xu, X., He, F., Wang, D., Wang, R., Wang, Y., Wang, G., et al. (2022). Rice catalase OsCATC is degraded by E3 ligase APIP6 to negatively regulate immunity. Plant Physiol. **190**:1095–1099. https://doi.org/10.1093/plphys/kiac317.

Zhang, L., Tian, L.H., Zhao, J.F., Song, Y., Zhang, C.J., and Guo, Y. (2009). Identification of an Apoplastic protein involved in the initial phase of salt stress response in rice root by two-dimensional electrophoresis. Plant Physiol **149**:916–928. https://doi.org/10.1104/pp.108.131144.

Zhao, C., Tang, Y., Wang, J., Zeng, Y., Sun, H., Zheng, Z., Su, R., Schneeberger, K., Parker, J.E., and Cui, H. (2021). A mis-regulated cyclic nucleotide-gated channel mediates cytosolic calcium elevation and activates immunity in Arabidopsis. New Phytol. **230**:1078–1094. https://doi.org/10.1111/nph.17218.

Zhao, Z., Sun, A., Shan, W., Zheng, X., Wang, Y., Bai, L., Xu, Y., An, Z., Wang, X., Wang, Y., and Fan, J. (2024). OsRbohI is the indispensable NADPH oxidase for molecular-patterns-induced reactive oxygen species production in rice. Plant Commun. **5**:101129. https://doi.org/10.1016/j.xplc.2024.101129.

Zheng, H., Chen, S., Chen, X., Liu, S., Dang, X., Yang, C., Giraldo, M.C., Oliveira-Garcia, E., Zhou, J., Wang, Z., and Valent, B. (2016). The Small GTPase MoSec4 Is Involved in Vegetative Development and Pathogenicity by Regulating the Extracellular Protein Secretion in *Magnaporthe oryzae*. Front. Plant Sci. **7**:1458. https://doi.org/10.3389/fpls.2016.01458.

Zhu, Y., Su, H., Liu, X.X., Sun, J.F., Xiang, L., Liu, Y.J., Hu, Z.W., Xiong, X.Y., Yang, X.M., Bhutto, S.H., et al. (2024). Identification of NADPH Oxidase Genes Crucial for Rice Multiple Disease Resistance and Yield Traits. Rice **17**:1. https://doi.org/10.1186/s12284-023-00678-5.

Plant Communications, Volume *7*

Supplemental information

Cysteine-rich secreted protein OsCRRSP1 enhances rice defense through dual inhibition of pathogen and host catalases

Zaofa Zhong, Zichao Zheng, Minghui Zhai, Gan Wang, Lijing Zhong, Huakun Zheng, Li Xue, and Haitao Cui

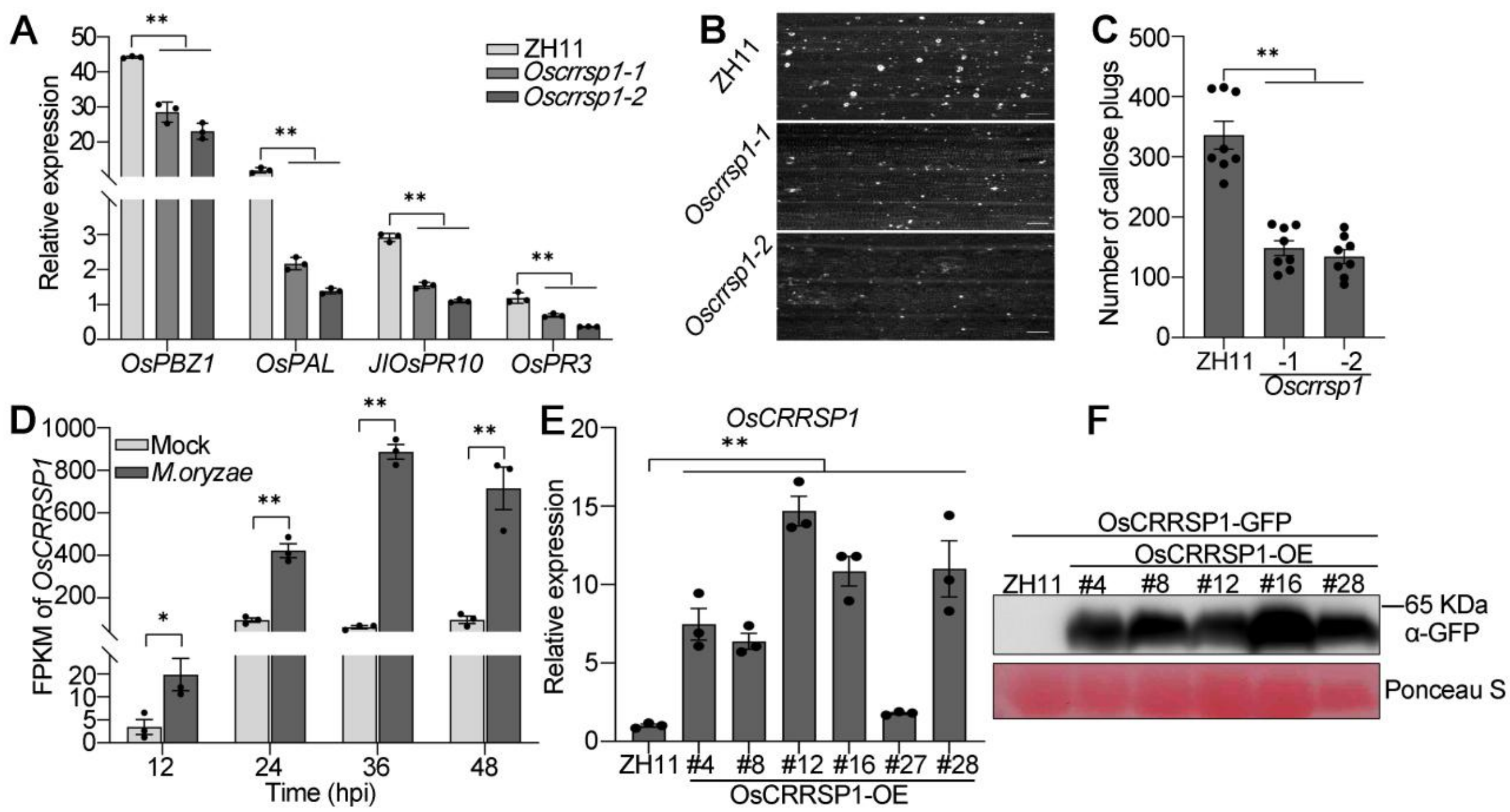

Figure S1. Molecular characterization of *OsCRRSP1* mutants and overexpression lines.

(A) Expression levels of defense-related genes (*OsPBZ1*, *OsPAL*, *JIOsPR10*, *OsPR3*) in ZH11 and *Oscrrsp1* mutants at 48 hpi after inoculation with Guy11, analyzed by qPCR and normalized to *OsUBQ*. Data represent means ± SE ($n = 3$). *$p < 0.05$, **$p < 0.01$ (Student's *t*-test vs. ZH11).

(B) Callose deposition in leaves of ZH11 and *Oscrrsp1* mutants in response to chitin treatment (800 nM), visualized by aniline blue staining under UV fluorescence. Scale bars, 50 μm.

(C) Quantification of callose deposits from (**B**) using ImageJ software. Data represent means ± SE ($n = 8$). *$p < 0.05$, **$p < 0.01$ (Student's *t*-test vs. ZH11).

(D) Transcript levels of *OsCRRSP1* in rice leaves at 12, 24, 36, and 48 hours post-inoculation with *M. oryzae* Guy11 or mock treatment (H_2O), based on RNA-seq data. Data represent mean ± SE ($n = 3$), *$p < 0.05$, **$p < 0.01$ (Student's *t*-test vs. H_2O treatment).

(E) Relative expression of *OsCRRSP1* in leaves of three-week-old ZH11 and OsCRRSP1-OE seedlings, determined by qPCR and normalized to *OsUBQ*. Data represent mean ± SE ($n = 3$); *$p < 0.05$, **$p < 0.01$ (Student's *t*-test vs. ZH11).

(F) Immunoblot analysis of OsCRRSP1-GFP fusion protein in total protein extracts from leaves of four-week-old ZH11 and two independent OsCRRSP1-OE lines, using an anti-GFP antibody. Ponceau S staining of Rubisco large subunit (Rbcl) serves as a loading control.

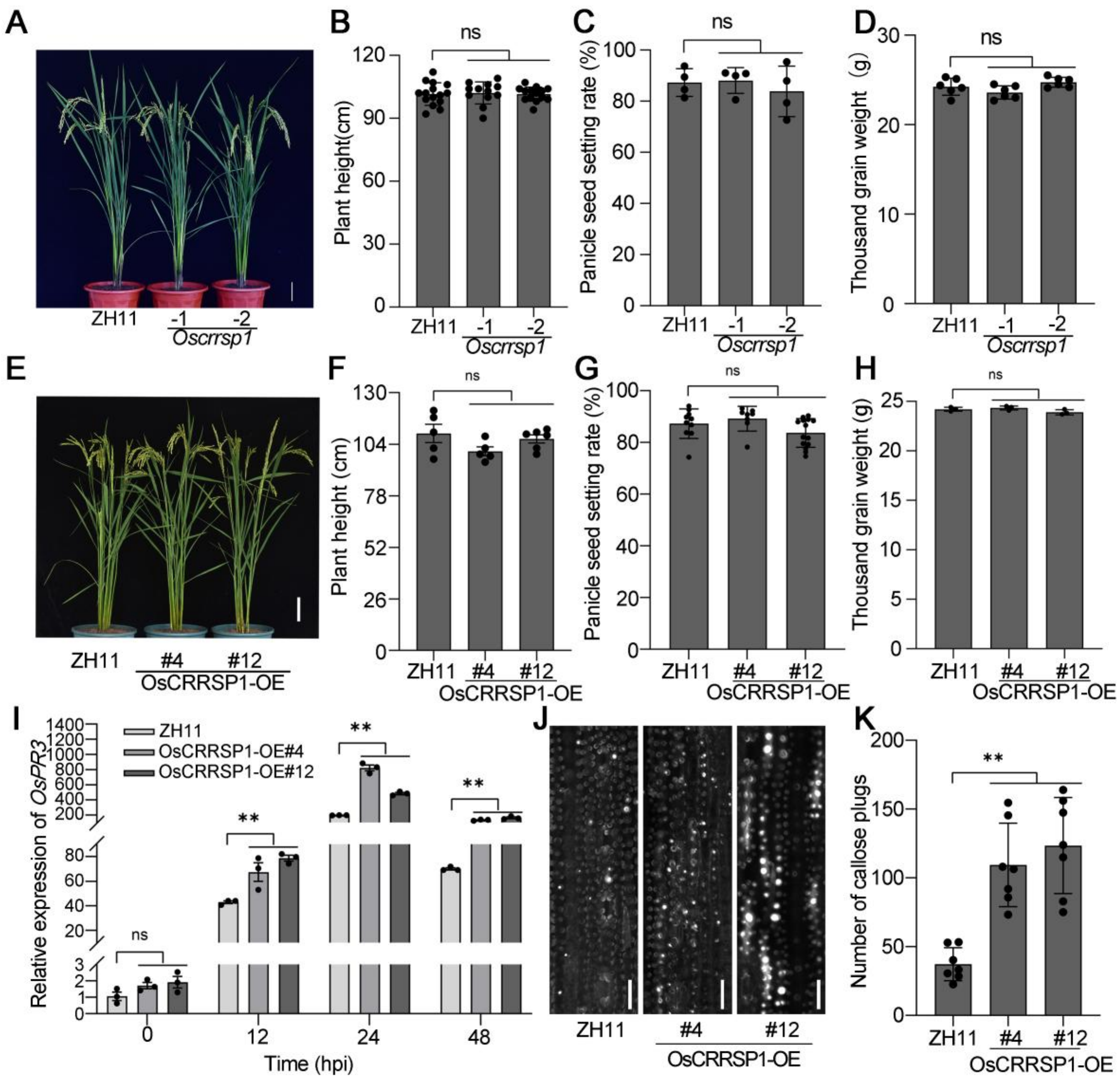

Figure S2. Agronomic traits of OsCRRSP1 mutants and overexpression lines and PTI immune responses in OsCRRSP1-OE plants.

(A) Representative images of ZH11 and *Oscrrsp1* mutant plants at the mature stage under field conditions. Scale bar, 10 cm.

(B) Plant height of ZH11 and *Oscrrsp1* mutants at maturity. Data represent mean ± SE (n = 12); ns, not significant ($p > 0.05$, Student's t-test).

(C) Panicle seed-setting rate of ZH11 and *Oscrrsp1* mutants. Data represent mean ± SE (n = 4); ns, not significant ($p > 0.05$, Student's t-test).

(D) Thousand-grain weight of ZH11 and *Oscrrsp1* mutants. Data represent mean ± SE (n = 6), ns, not significant ($p > 0.05$, Student's t-test).

(E) Field growth phenotypes of ZH11 and OsCRRSP1-OE plants at maturity. Scale bar, 10 cm.

(F) Plant height of ZH11 and OsCRRSP1-OE plants. Data represent mean ± SE (*n* = 12); ns, not significant ($p > 0.05$, Student's *t*-test).

(G) Panicle seed-setting rate of ZH11 and OsCRRSP1-OE plants. Data represent mean ± SE (*n* = 4), ns, not significant ($p > 0.05$, Student's *t*-test).

(H) Thousand-grain weight of ZH11 and OsCRRSP1-OE plants. Data represent mean ± SE (*n* = 6), ns, not significant ($p > 0.05$, Student's *t*-test).

(I) Expression levels of OsPR3 genes in ZH11 and OsCRRSP1-OE plants at indicated time points after *M. oryzae* infection, analyzed by RT–qPCR and normalized to OsUBQ. Data represent means ± SE (*n* = 3). *$p < 0.05$, **$p < 0.01$ (Student's *t*-test vs. ZH11).

(J) Callose deposition in leaves of ZH11 and OsCRRSP1-OE plants in response to chitin elicitation (800 nM), visualized by aniline blue staining under UV epifluorescence microscopy. Scale bars, 50 μm.

(K) Quantification of callose deposits from (**J**) using ImageJ software. Data represent means ± SE (*n* = 7). *$p < 0.05$, **$p < 0.01$(Student's *t*-test vs. ZH11).

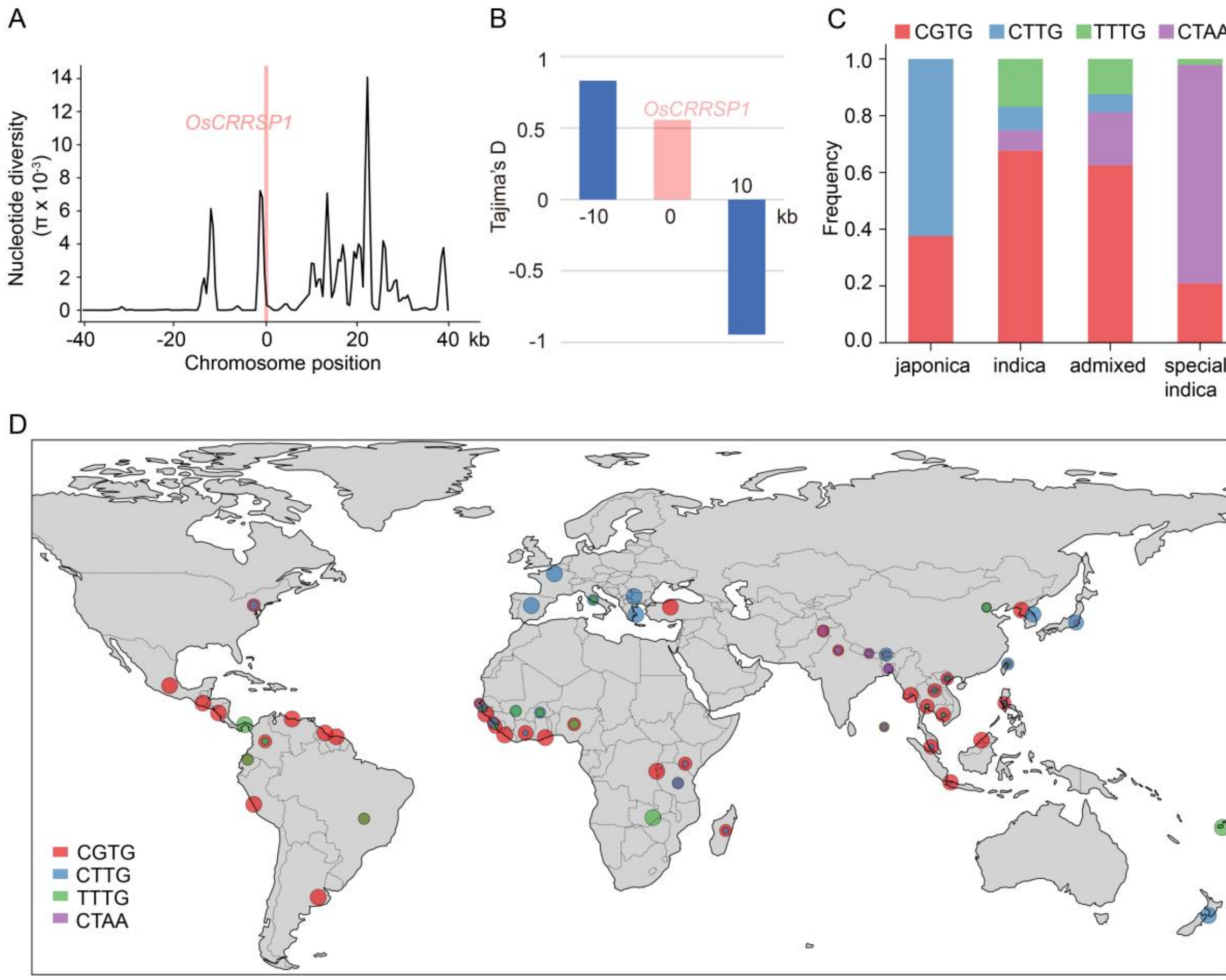

Supplemental Figure 3

Figure S3. Natural variation and geographic distribution of *OsCRRSP1* haplotypes in rice.

(A) Sliding-window analysis of nucleotide diversity (π) across a 40-kb genomic region surrounding *OsCRRSP1* in rice accessions. π was calculated using 1-kb windows with a 500-bp step size. The coding region of *OsCRRSP1* is indicated by a shaded box.

(B) Tajima's D values across *OsCRRSP1* genomic region.

(C) Haplotype composition of *OsCRRSP1* based on four polymorphic sites across rice subpopulations. The frequency of each haplotype (CGTG, CTAA, CTTG, and TTTG) is shown for indica, japonica, and other subgroups.

(D) Geographic distribution of major *OsCRRSP1* haplotypes across global rice accessions. Different colors indicate distinct haplotypes.

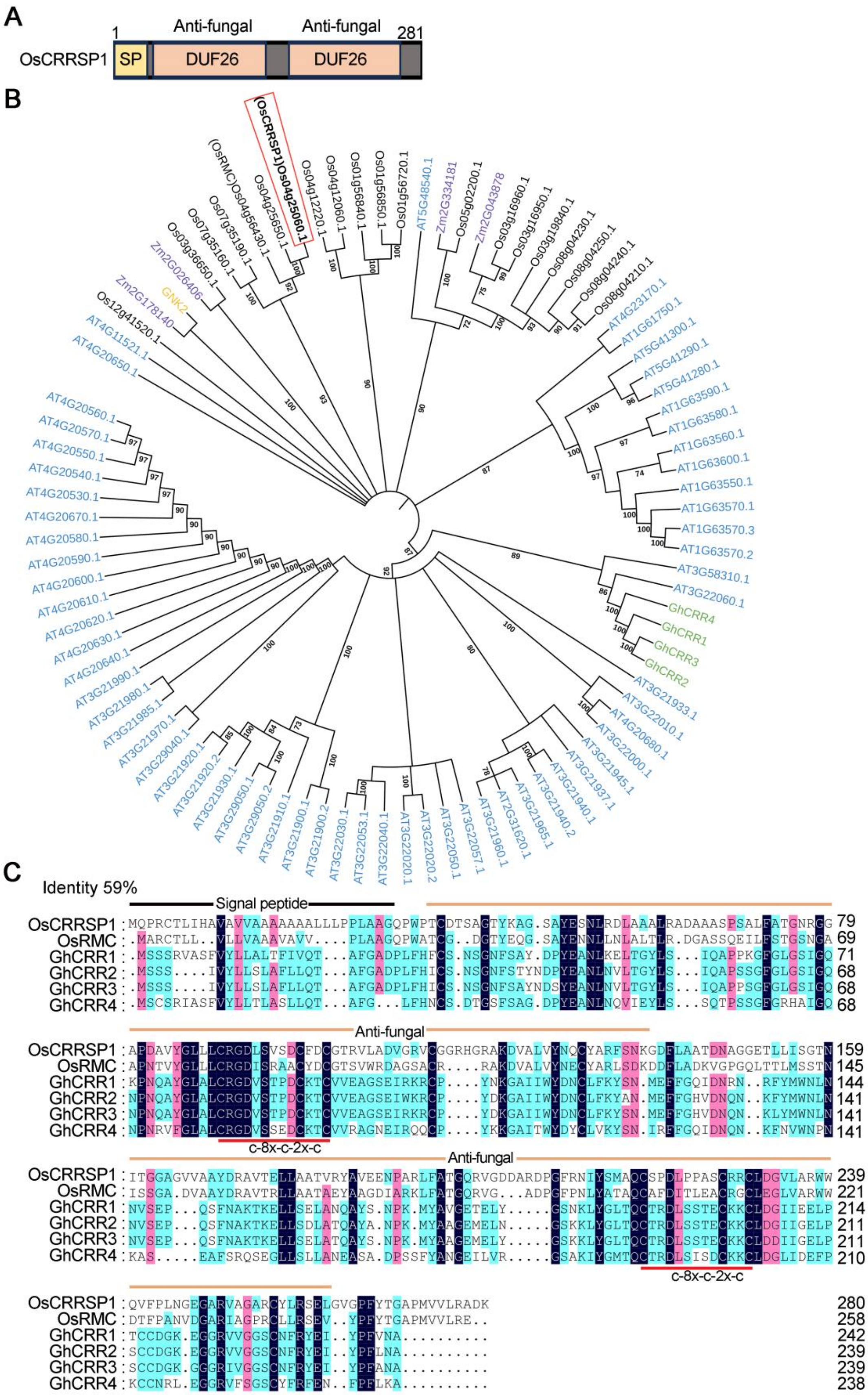
A
OsCRRSP1
1
SP
Anti-fungal
DUF26
Anti-fungal
DUF26
281
B
(OsCRRSP1)Os04g25060.1
(OsRMC)Os04g56430.1
GNK2
GhCRR1
GhCRR2
GhCRR3
GhCRR4
C
Identity 59%
Signal peptide
Anti-fungal
Anti-fungal
c-8x-c-2x-c
c-8x-c-2x-c
OsCRRSP1
OsRMC
GhCRR1
GhCRR2
GhCRR3
GhCRR4

Figure S4. Protein domain, evolutionary tree analysis, and Sequence alignment of OsCRRSP1.

(A) Schematic diagram of the OsCRRSP1 protein structure, showing the N-terminal signal peptide (SP) and two domains of unknown function (DUF26). Numbers indicate amino acid (aa) positions.

(B) Phylogenetic tree of CRRSP proteins in rice. The phylogenetic tree was generated using MEGA 7.0. In the phylogenetic tree, CRRSP members from rice are highlighted in black. The gene IDs of CRRSP members from *Arabidopsis thaliana* are marked in blue. Four CRR members from cotton are indicated in green, four CRRSP members from maize are shown in purple, and Gnk2 from *Ginkgo biloba* is labeled in yellow.

(C) Multiple sequence alignment of OsCRRSP1 with its homologs from rice (OsRMC) and cotton (GhCRR1, GhCRR2, GhCRR3, GhCRR4). The conserved cysteine residues forming the characteristic C-8X-C-2X-C motifs in the DUF26 domains are indicated underlined in red. Sequence identities were calculated using Clustal Omega.

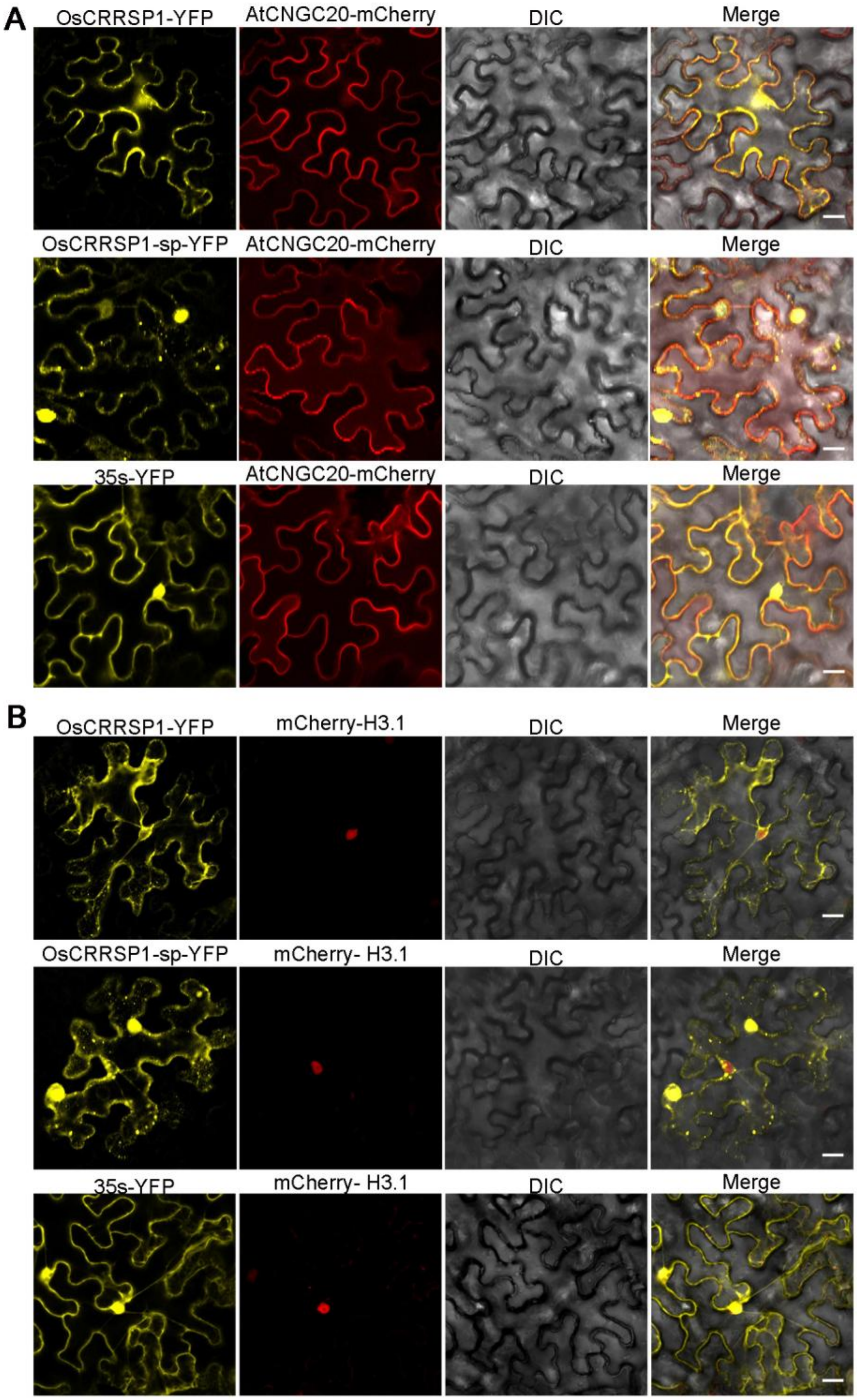

Figure S5 Subcellular localization analysis of OsCRRSP1.

(A) Subcellular localization of full-length OsCRRSP1-YFP and signal peptide-deleted $OsCRRSP1_{\Delta SP}$-YFP (yellow) co-expressed with the plasma membrane marker AtCNGC20-mCherry (red) in *N. benthamiana* epidermal cells. 35S-YFP was used as a cytosolic control. Images were acquired by confocal microscopy 48 hours after agroinfiltration. Scale bar, 20 μm.

(B) Subcellular localization of OsCRRSP1-YFP and OsCRRSP1$_{\Delta SP}$-YFP (yellow) co-expressed with the nuclear marker mCherry-Histone H3.1 (red) in *N. benthamiana* epidermal cells. 35S-YFP was used as a cytosolic control. Images were acquired by confocal microscopy 48 hours after agroinfiltration. Scale bar, 20 μm.

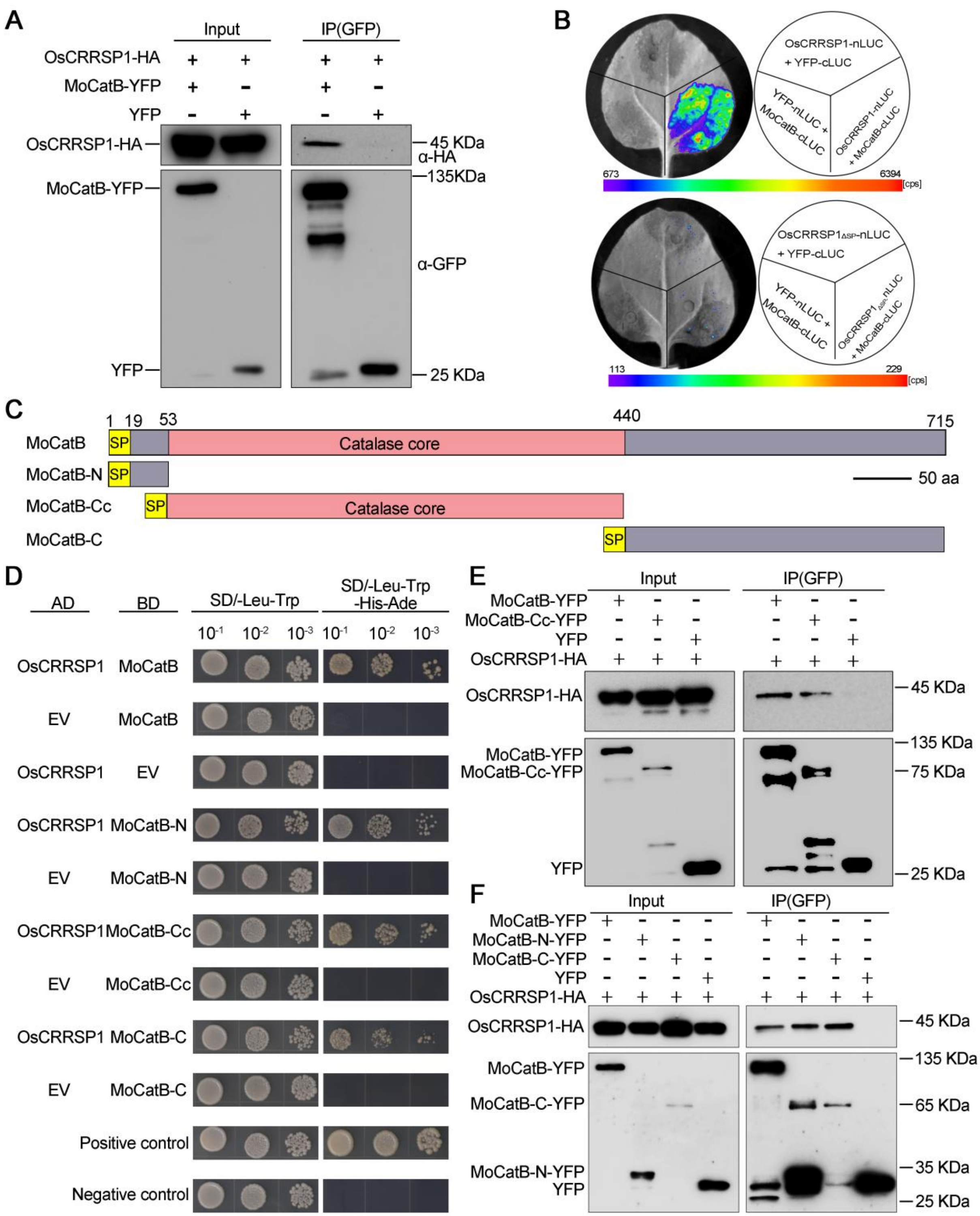

Figure S6. OsCRRSP1 interacts with MoCatB through multiple regions.

(A) Co-immunoprecipitation (co-IP) analysis of the interaction between MoCatB and OsCRRSP1 *in planta*. Total proteins were extracted from *N. benthamiana* leaves co-expressing MoCatB-GFP and OsCRRSP1-HA, or the control (YFP and OsCRRSP1-HA), at 48 hours post-agroinfiltration. Proteins were immunoprecipitated (IP)

using GFP-Trap beads and subsequently immunoblotted with the indicated antibodies. "Input" represents 2.5% of the total protein extract used for the IP. Molecular mass markers are indicated in kilodaltons (kD).

(B) Split-luciferase complementation assay testing the interaction between signal peptide-deleted OsCRRSP1 (OsCRRSP1$_{\Delta SP}$-nLUC) and MoCatB-cLUC in *N. benthamiana* leaves. The combinations OsCRRSP1$_{\Delta SP}$-nLUC + cLUC and nLUC + MoCatB-cLUC served as negative controls. Luciferase activity was imaged 48 hours post-infiltration.

(C) Schematic diagram of the MoCatB protein structure and construction of truncated vector. The yellow box represents the predicted N-terminal signal peptide (SP), the pink box represents the predicted Catalase core (Cc) domain. The SP shown in this diagram were all removed in the Y2H experiment, while they were retained in the Co-IP experiment. Numbers indicate amino acid (aa) positions.

(D) Yeast two-hybrid assays examining interactions of OsCRRSP1 with MoCatB-N, MoCatB-Cc, and MoCatB-C. Yeast strain AH109 co-transformed with the indicated bait (BD) and prey (AD) constructs was grown on synthetic defined medium lacking Leu and Trp (SD/-Leu/-Trp) or selective medium lacking Leu, Trp, His, and Ade (SD/-Ade/-His/-Leu/-Trp). The AD-T + BD-53 pair served as a positive control, while AD + BD, AD + BD-MoCatB-N, AD + BD-MoCatB-Cc, AD + BD-MoCatB-C and AD-OsCRRSP1 + BD were negative controls. Plates were incubated at 30°C for 3 days.

(E) Co-immunoprecipitation (Co-IP) analysis of the interaction between OsCRRSP1 and the catalase core domain of MoCatB (MoCatB-Cc) in planta. Total proteins were extracted from *N. benthamiana* leaves co-expressing OsCRRSP1-HA and MoCatB-Cc -GFP, or the control (YFP and OsCRRSP1-HA), at 48 hours post-agroinfiltration. Proteins were immunoprecipitated (IP) using GFP-Trap beads and subsequently immunoblotted with the indicated antibodies. "Input" represents 2.5% of the total protein extract used for the IP.

(F) Co-immunoprecipitation (Co-IP) analysis of the interactions of MoCatB-N and MoCatB-C with OsCRRSP1 in planta. Total proteins were extracted from rice protoplasts co-expressing MoCatB-GFP and OsCRRSP1-HA, MoCatB-N-GFP and OsCRRSP1-HA, MoCatB-C-GFP and OsCRRSP1-HA or the control (YFP and OsCRRSP1-HA). Proteins were immunoprecipitated (IP) using GFP-Trap beads and subsequently immunoblotted with the indicated antibodies. "Input" represents 2.5% of the total protein extract used for the IP.

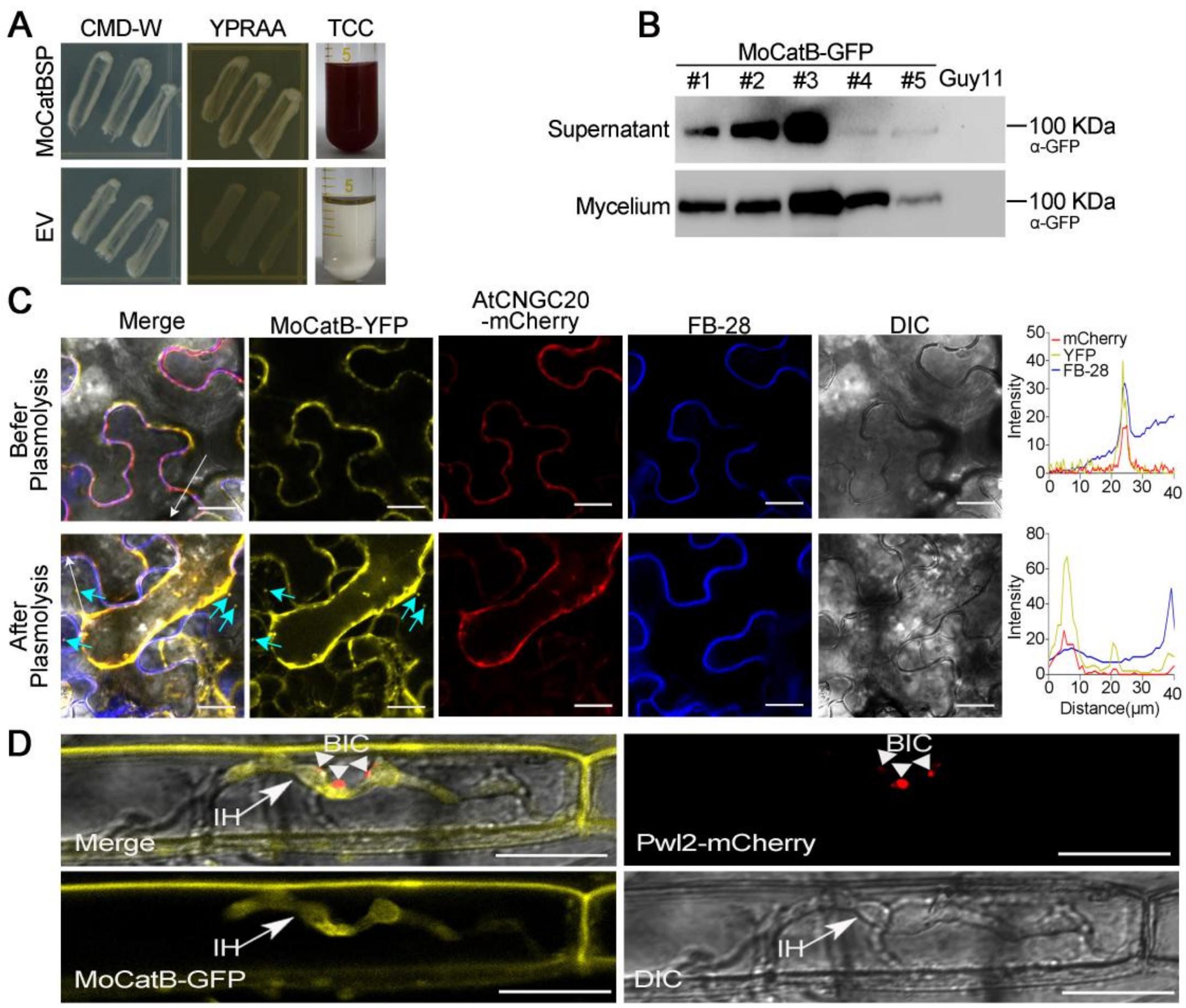

Figure S7 *M. oryzae* MoCatB is a secreted protein.

(A) Functional analysis of the MoCatB signal peptide (MoCatBSP) using the yeast invertase secretion assay. Yeast strain YTK12 expressing pSUC2-MoCatBSP or the empty pSUC2 vector (negative control) were grown on CMD-W (tryptophan-deficient) or YPRAA (raffinose-based) medium. Invertase secretion was confirmed by the reduction of triphenyltetrazolium chloride (TTC) to red formazan.

(B) Immunoblot analysis detecting MoCatB-GFP secretion in *M. oryzae*. Proteins from mycelial extracts (Mycelium) and culture supernatant (Supernatant) of these MoCatB-GFP strains (#1-5) were probed with an anti-GFP antibody. The wild-type Guy11 strain served as a negative control.

(C) MoCatB is located in the plant apoplast. Subcellular localization of MoCatB-YFP (yellow) co-expressed with the plasma membrane marker AtCNGC20-mCherry (red) in *N. benthamiana* epidermal cells before and after plasmolysis. Cell walls were stained with Fluorescent Brightener 28 (FB-28; blue). Images were captured by confocal microscopy before and after plasmolysis induced by 1.5 M sorbitol. Blue arrows indicate MoCatB-YFP signal in the apoplastic space post-plasmolysis. The fluorescence curves were obtained following the direction of the white arrows. Scale bars, 20 μm.

(D) Confocal microscopy images of rice sheath cells infected with an *M. oryzae* strain co-expressing MoCatB-GFP (green) and the cytoplasmic effector Pwl2-mCherry (red) at 24 hours post-inoculation (hpi). The biotrophic

interfacial complex (BIC) is indicated by a white arrowhead. Scale bars, 20 μm.

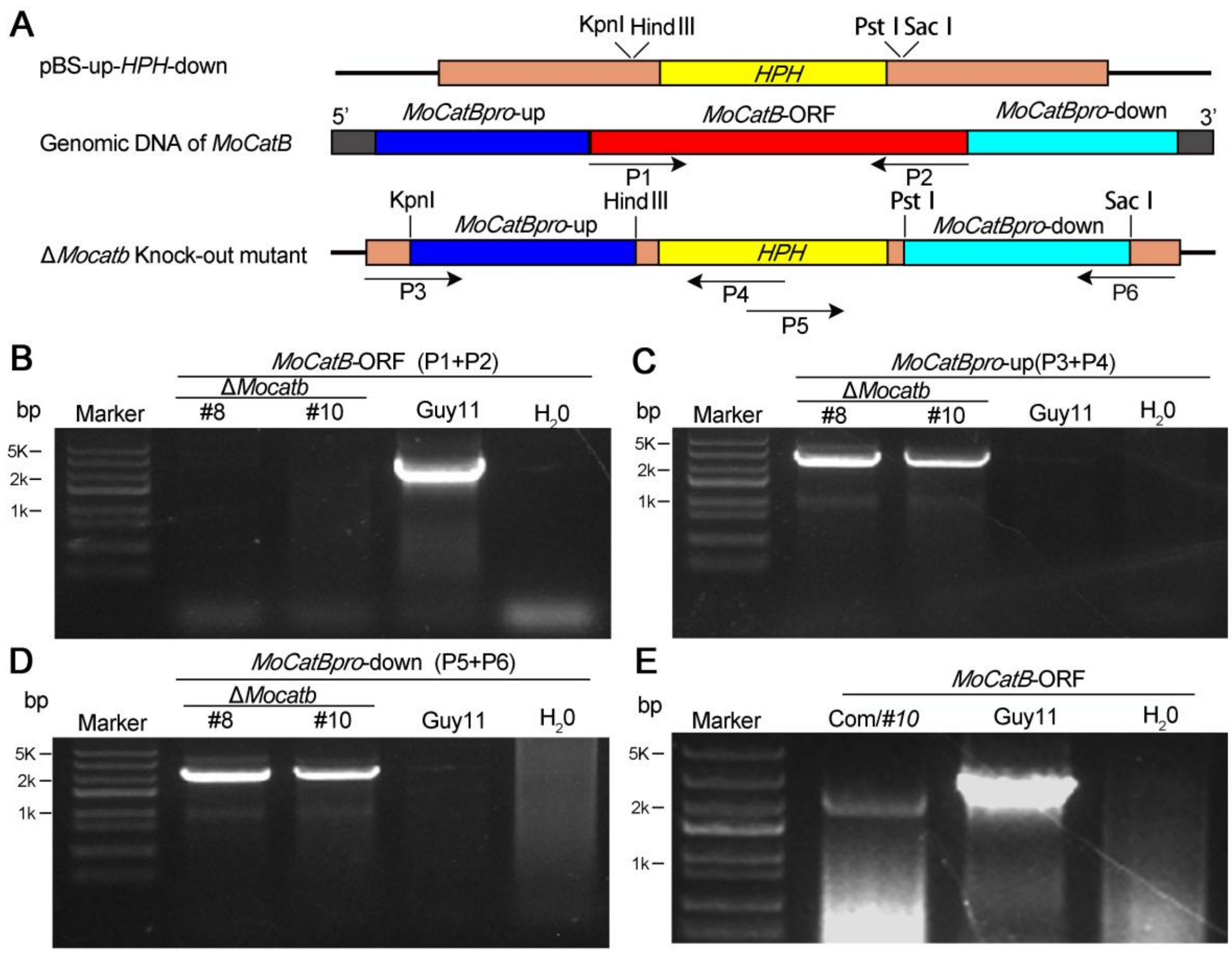

Figure S8. Generation and molecular characterization of *MoCatB* knockout and complementation strains.

(A) Strategy for targeted deletion of *MoCatB* in *M. oryzae* strain Guy11 via homologous recombination. The hygromycin phosphotransferase (*HPH*) cassette replaces the *MoCatB* coding sequence. Arrows indicate primer positions (P1-P4) used for mutant verification. Schematic not drawn to scale.

(B) PCR verification of *ΔMocatb* mutants using primers spanning the full-length *MoCatB* open reading frame (ORF). Genomic DNA from Guy11 produces the expected amplicon, while no product is amplified from the *ΔMocatb* mutants (#8 and #10).

(C) PCR verification of *ΔMocatb* mutants using primers specific to the upstream flanking region of *MoCatB*. The expected fragment is present in both Guy11 and the mutants.

(D) PCR verification of *ΔMocatb* mutants using primers specific to the downstream flanking region of *MoCatB*. The expected fragment is present in both Guy11 and the mutants.

(E) PCR verification of the complemented strain (Com/#10) using primers specific to the *MoCatB* ORF. Guy11 and Com/#10 show the expected amplification, while no product is detected in the *ΔMocatb*#10 mutant. H_2O serves as a no-template control.

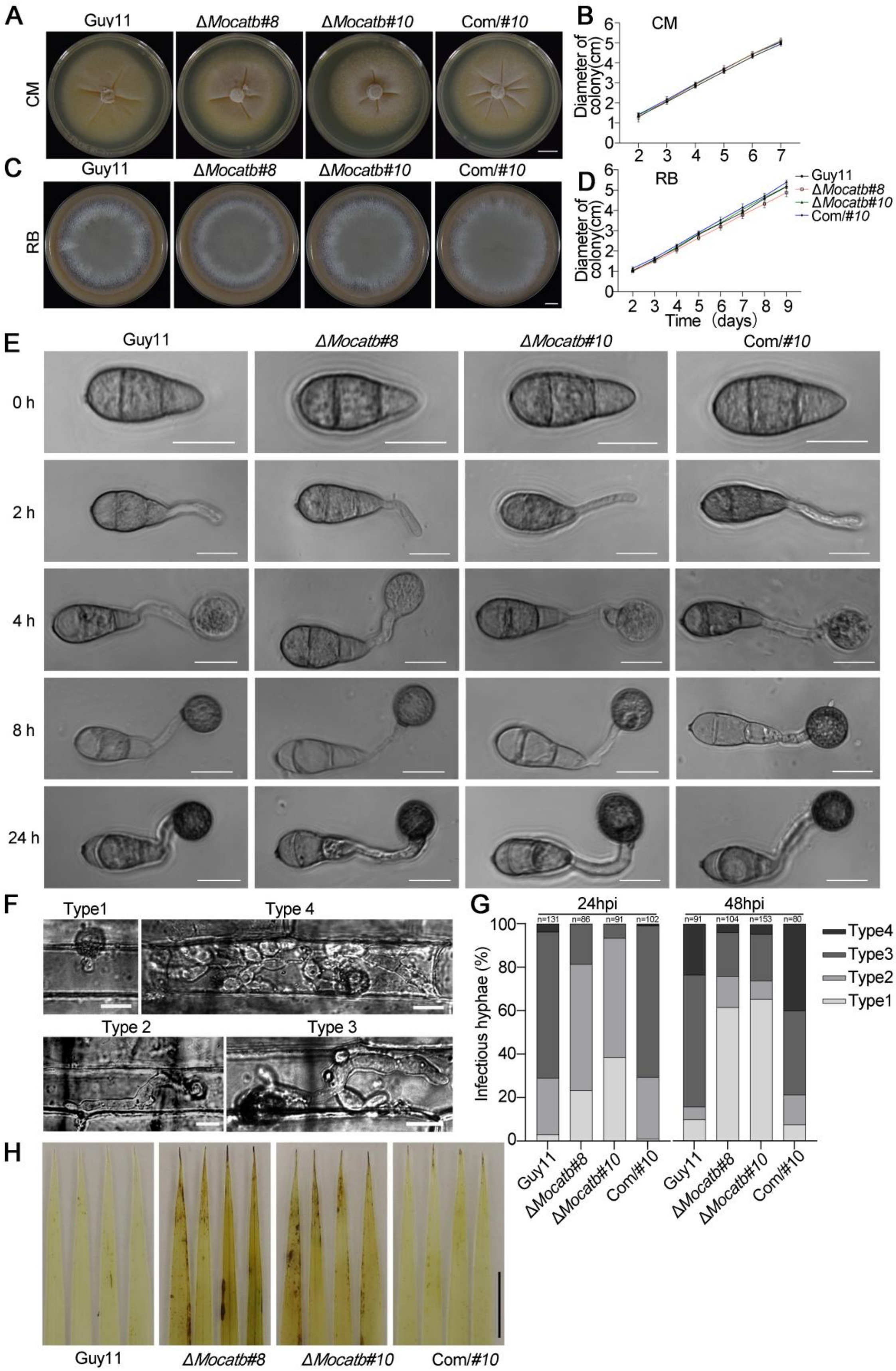
A
Guy11
ΔMocatb#8
ΔMocatb#10
Com/#10
CM
B
CM
Diameter of colony(cm)
C
RB
D
RB
Guy11
ΔMocatb#8
ΔMocatb#10
Com/#10
Time (days)
E
0 h
2 h
4 h
8 h
24 h
F
Type1
Type 4
Type 2
Type 3
G
24hpi
48hpi
n=131
n=86
n=91
n=102
n=91
n=104
n=153
n=80
Infectious hyphae (%)
Type4
Type3
Type2
Type1
H

149

Figure S9. *MoCatB* deletion does not affect *in vitro* growth but impairs invasive hyphal development and host ROS suppression.

(A) Colony morphology of Guy11, *ΔMocatb* mutants (#8, #10), and complemented strain (Com/#10) after 7 days of growth on complete medium (CM) at 28°C.

(B) Mycelial growth rates of strains in (**A**). Data represent mean ± SE (n = 6).

(C) Colony morphology of indicated strains after 9 days of growth on rice bran (RB) medium at 28°C.

(D) Mycelial growth rates of strains in (**C**). Data represent mean ± SE (n = 6).

(E) Conidial germination and appressorium formation of indicated strains on hydrophobic surfaces at 2, 4, and 8 hours post-incubation. Scale bar, 10 µm.

(F) Classification of invasive hyphal (IH) types in rice sheath cells: Type 1, filamentous primary IH; Type 2, single bulbous IH; Type 3, branched bulbous IH; Type 4, IH occupying entire plant cell and penetrating neighboring cells. Scale bar, 10 µm.

(G) Percentage distribution of IH types at 24 and 48 hours post-inoculation (hpi) with indicated strains. At least 80 infection sites were examined per strain per time point across three independent experiments.

(H) DAB staining detecting H_2O_2 accumulation in ZH11 leaves at 48 hpi with Guy11, *ΔMocatb* mutants (#8, #10), or Com/#10. Scale bar, 1 cm.

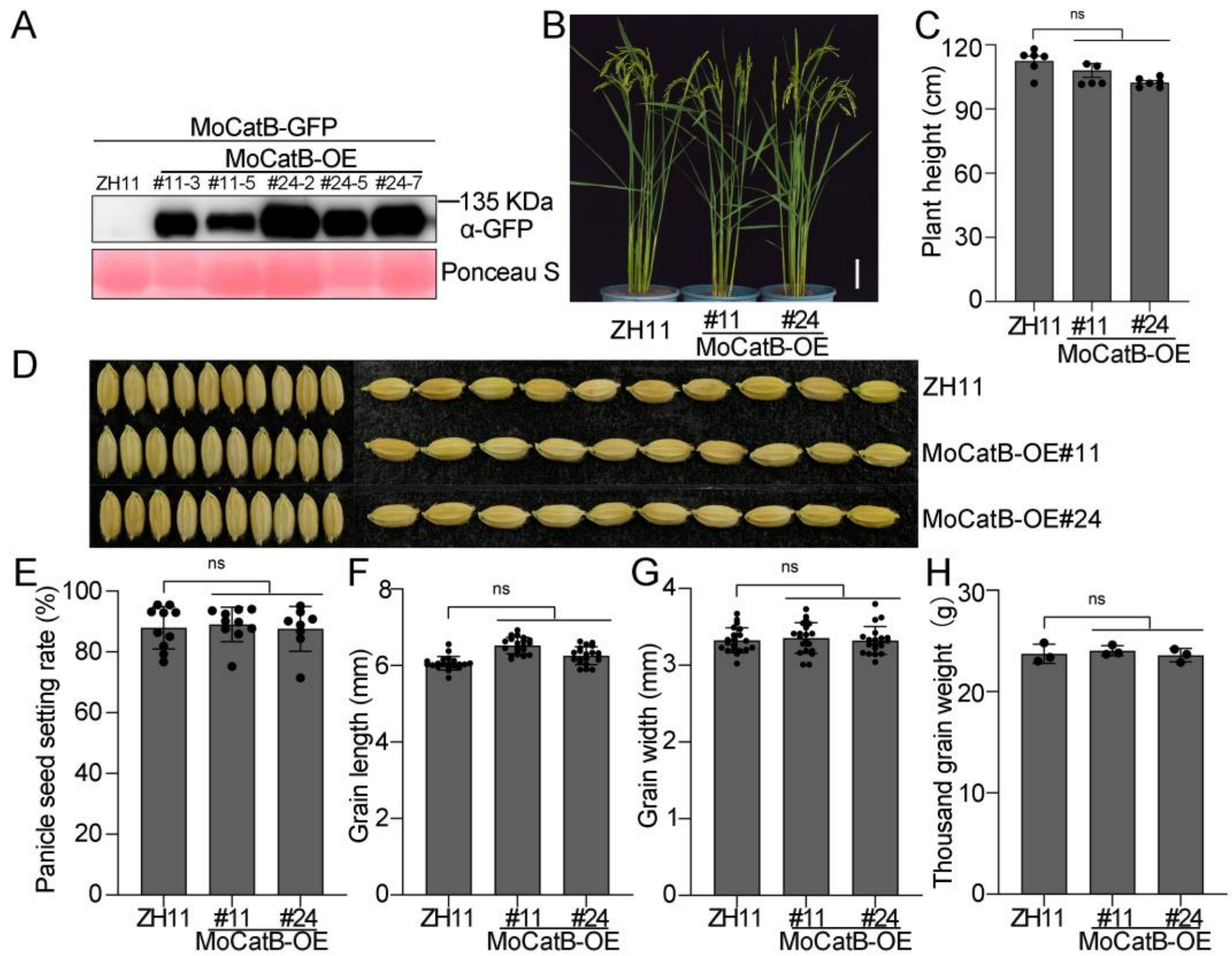

Figure S10. Overexpression of *MoCatB* does not affect major agronomic traits in rice.

(A) Immunoblot analysis of MoCatB-GFP fusion protein in total protein extracts from leaves of three-week-old ZH11 and two independent MoCatB-OE lines using an anti-GFP antibody. Ponceau S staining of Rubisco large subunit (Rbcl) serves as a loading control.

(B) Representative images of ZH11 and MoCatB-OE plants at the mature stage under field conditions. Scale bar, 10 cm.

(C) Plant height of ZH11 and MoCatB-OE plants at maturity. Data represent mean ± SE (n = 12). ns, not significant.

(D) Mature seeds of ZH11 and MoCatB-OE plants. Scale bar, 1 cm.

(E) Panicle seed-setting rate of ZH11 and MoCatB-OE plants. Data represent mean ±SE (n = 4). ns, not significant. *$p < 0.05$ (Student's t -test).

(F) Grain length of ZH11 and MoCatB-OE plants. Data represent mean ± SE (n = 20). ns, not significant. *$p < 0.05$ (Student's t -test)

(G) Grain width of ZH11 and MoCatB-OE plants. Data represent mean ± SE (n = 20). ns, not significant. *$p < 0.05$ (Student's t -test)

(H) Thousand-grain weight of ZH11 and MoCatB-OE plants. Data represent mean ± SE (n = 6). ns, not significant. *$p < 0.05$ (Student's t -test).

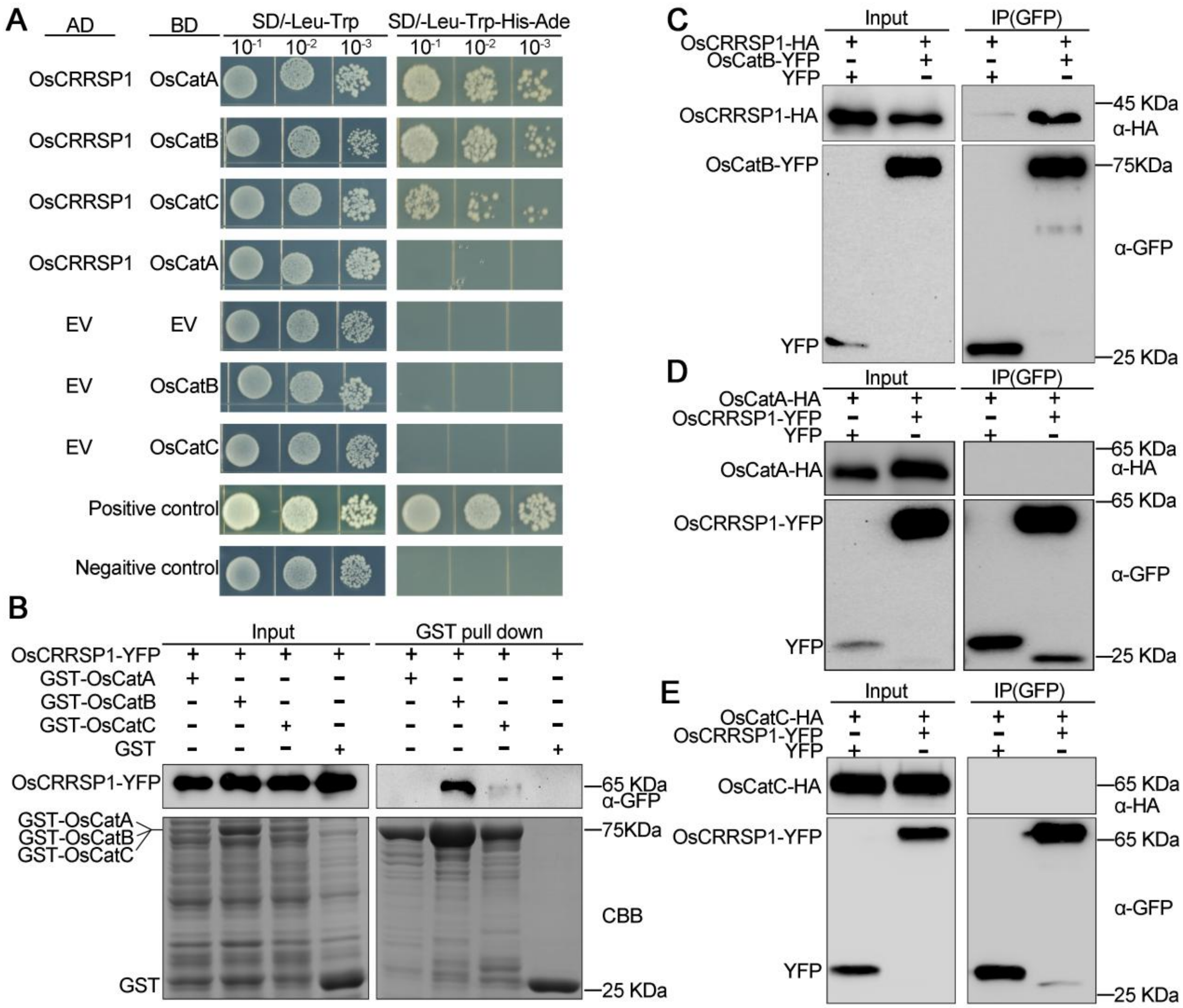

Figure S11. Specificity analysis of OsCRRSP1 interaction with rice catalase family members.

(A) Yeast two-hybrid assay screening the interaction between OsCRRSP1 and three rice catalases (OsCatA, OsCatB, OsCatC). Yeast strain AH109 co-expressing the indicated bait (BD) and prey (AD) constructs was grown on synthetic defined medium lacking Leu and Trp (SD/-Leu/-Trp) or selective medium lacking Leu, Trp, His, and Ade (SD/-Ade/-His/-Leu/-Trp). The AD-T + BD-53 pair served as a positive control, while the combinations AD + BD, AD + BD-OsCatA/B/C, and AD-OsCRRSP1 + BD served as negative controls. Plates were incubated at 30°C for 3 days.

(B) GST pull-down assays assessing direct interactions between OsCRRSP1 and rice catalases. Recombinant GST-OsCatA, GST-OsCatB, GST-OsCatC or GST (control) immobilized on glutathione-sepharose beads were incubated with purified OsCRRSP1-YFP. Bound proteins were analyzed by immunoblotting with an anti-GFP antibody (top panels). The bottom panels show Coomassie Brilliant Blue (CBB) staining of the bait proteins.

(C) Co-IP analysis of the interaction between OsCatB and OsCRRSP1 in planta. Total proteins were extracted from *N. benthamiana* leaves co-expressing OsCatB-YFP and OsCRRSP1-HA, or the control (YFP and

OsCRRSP1-HA), at 48 hours post-agroinfiltration. Proteins were immunoprecipitated (IP) using GFP-Trap beads and subsequently immunoblotted with the indicated antibodies. "Input" represents 2.5% of the total protein extract used for the IP.

(D) Co-IP assay testing the interaction between OsCRRSP1 and OsCatA *in planta*. Total proteins were extracted from *N. benthamiana* leaves co-expressing OsCRRSP1-YFP and OsCatA-HA, or the control (YFP and OsCatA-HA), at 48 hours post-agroinfiltration. Proteins were immunoprecipitated using GFP-Trap beads and immunoblotted with the indicated antibodies. "Input" represents 2.5% of the total protein extract.

(E) Co-IP assay testing the interaction between OsCRRSP1 and OsCatC *in planta*. Experimental conditions and controls are as described in panel (**D**).

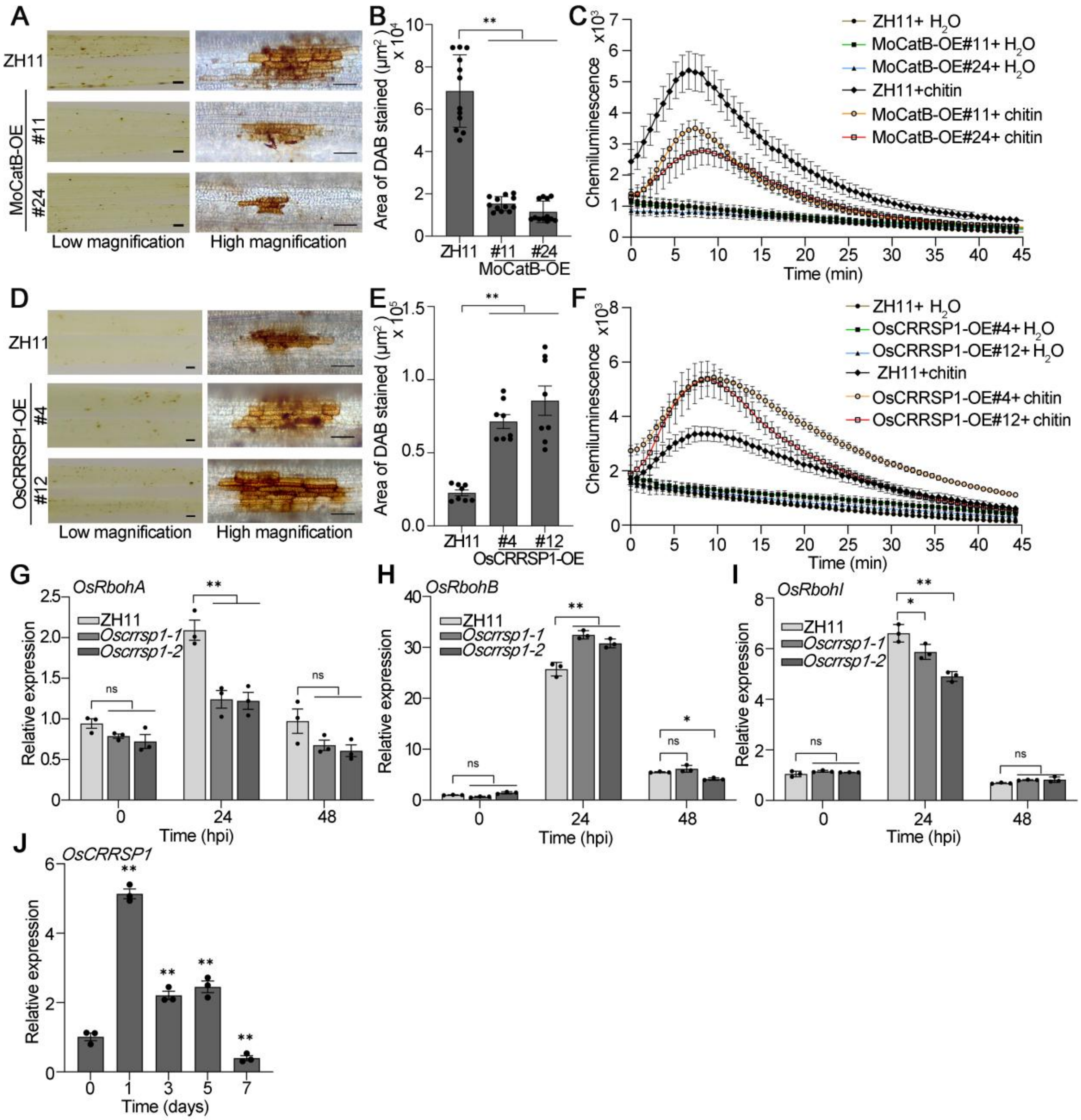

Figure S12 OsCRRSP1 promotes pathogen-induced ROS accumulation.

(A) Detection of H_2O_2 accumulation by 3,3'-diaminobenzidine (DAB) staining in leaves of ZH11 and *MoCatB-OE* plants at 48 hours post-inoculation with *M. oryzae* Guy11. Low-magnification (top) and high-magnification (bottom) images are shown. Scale bars, 1 mm (top) or 100 μm (bottom).

(B) Quantification of DAB-stained area from (**A**) using ImageJ. Data represent mean ± SE (n = 8). *$p < 0.05$, **$p < 0.01$(Student's t-test vs. ZH11).

(C) Chitin-induced ROS burst in leaf discs of ZH11 and *MoCatB-OE* plants treated with 800 nM chitin. Luminescence was measured over 45 minutes. Data represent mean ± SE (n = 12).

(D) DAB staining detecting H_2O_2 accumulation in ZH11 and OsCRRSP1-OE plants at 48 hpi with Guy11. Scale

bars, 1 mm (top) or 100 μm (bottom).

(E) Quantification of DAB-stained area from (**D**) by ImageJ. Data represent mean ± SE (n = 8). *p < 0.05, **p < 0.01 (Student's t-test vs. ZH11).

(F) Chitin-induced ROS burst in ZH11 and OsCRRSP1-OE leaf discs treated with 800 nM chitin. Data represent mean ± SE (n=8).

(G) Expression levels of *OsRbohA* in ZH11 and *Oscrrsp1* mutants after inoculation with Guy11, analyzed by RT-qPCR and normalized to *OsUBQ*. Data represent means ± SE (n = 3). *p < 0.05, **p < 0.01 (Student's t-test vs. ZH11).

(H) Expression levels of *OsRbohB* in ZH11 and *Oscrrsp1* mutants after inoculation with Guy11, analyzed by RT-qPCR and normalized to *OsUBQ*. Data represent means ± SE (n = 3). *p < 0.05, **p < 0.01 (Student's t-test vs. ZH11).

(I) Expression levels of *OsRbohI* in ZH11 and *Oscrrsp1* mutants after inoculation with Guy11, analyzed by RT-qPCR and normalized to *OsUBQ*. Data represent means ± SE (n = 3). *p < 0.05, **p < 0.01 (Student's t-test vs. ZH11).

(J) Expression analysis of *OsCRRSP1* in rice leaves inoculated with *Xanthomonas oryzae* pv. *Oryzae*. Samples were collected at the indicated time points and analyzed by RT-qPCR normalized to *OsUBQ*. The data represent mean ± SE (n = 3). **p < 0.01 (Student's t-test vs. 0 days).

Table S1. (separate file)

Haplotype composition of *OsCRRSP1* based on four polymorphic sites across rice subpopulations.

Table S2. (separate file)

Putative OsCRRSP1-interacting proteins identified by IP-MS in *M. oryzae*.

Table S3. (separate file)

The candidate proteins revealed by the Y2H screen.

Table S4. (separate file)

Primers used in this study.
